# Biofilm‐Binding Phages Enhance Biofilm Eradication by Synergistic Photothermal and Photodynamic Therapy

**DOI:** 10.1002/advs.202523904

**Published:** 2026-07-08

**Authors:** Ying Cao, Tao Yang, Rui Wang, Hui‐Da Li, Xiao‐Yu Zhang, Feng Cheng, Ying Liu, Jian‐Hua Wang, Ting Yang, Chuanbin Mao

**Affiliations:** ^1^ Research Center for Analytical Sciences Department of Chemistry College of Sciences Northeastern University Shenyang China; ^2^ Department of Biomedical Engineering The Chinese University of Hong Kong Hong Kong SAR China; ^3^ CAS Key Laboratory of Separation Science for Analytical Chemistry Dalian Institute of Chemical Physics Chinese Academy of Sciences Dalian China; ^4^ Department of Emergency General Hospital of Northern Theater Command Shenyang China

**Keywords:** biofilm, M13 phage, photodynamic therapy, photothermal therapy

## Abstract

Biofilms, a major cause of chronic bacterial infections, present significant treatment challenges due to their protective extracellular matrix that shields bacteria from both antibiotics and host immune defenses. To treat biofilms more effectively, here we discovered a biofilm‐binding peptide from a phage library and verified that it selectively bound the polysaccharides on the biofilm. We then engineered M13 phage into trifunctional nanofibers displaying the biofilm‐binding peptide at the tip and carrying gold nanoparticles (AuNPs, as photothermal agents) and tetrakis(4‐carboxyphenyl) porphyrin (TCPP, as a photosensitizer) on the sidewall for combined phototherapy. This design enhances binding/anchoring and eradication of biofilms by leveraging the unique properties of each component. The phage nanofibers efficiently bound biofilms and promoted the transfer of heat and penetration of reactive oxygen species (ROS) into the biofilm, leading to cell death. Therefore, under light irradiation, the engineered phage nanofibers effectively eradicated the biofilms by AuNP‐induced photothermal therapy (PTT) and TCPP‐assisted photodynamic therapy (PDT) in a biofilm‐associated skin wound model. Transcriptomic profiling suggests stress‐response signatures consistent with oxidative/thermal injury. This study presents a promising ternary synergistic strategy for eradicating biofilms, potentially improving clinical outcomes in wound infection management.

## Graphical abstract

Engineered phage‐based trifunctional nanofibers are developed for synergistic biofilm eradication. Biofilm‐binding phages, armed with AuNPs and TCPP, serve as both warheads and scaffolds, endowing the phototherapy agents with biofilm‐anchoring ability. Upon light irradiation, AuNPs induce photothermal therapy, while TCPP generates ^1^O_2_ for photodynamic therapy. Mild hyperthermia disrupts the biofilm and enhances ^1^O_2_ penetration, leading to efficient biofilm elimination.

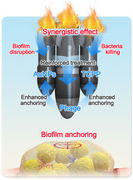

## 1 Introduction

An estimated 80% of chronic bacterial infections involve biofilm formation at the wound site [1, 2]. Bacterial biofilms are microbial communities that adhere to surfaces and produce a protective matrix, known as extracellular polymeric substances (EPS) [3]. EPS is a complex blend of diverse biopolymers, including polysaccharides, proteins, lipids, and extracellular DNA (eDNA), which make up the majority of the biomass within the biofilm, providing a suitable environment for microorganisms and serving as a source of active substances [4, 5, 6]. EPS also impedes the diffusion of antibiotics, making bacteria within biofilms 10–1000 times more resistant to conventional treatments compared to planktonic bacteria [7, 8]. This resistance complicates the treatment of wound infections, highlighting the urgent need for developing new anti‐biofilm agents to disrupt biofilms and eliminate infections.

Phage therapy is a promising alternative to traditional antibiotics, using virulent phages that specifically target and kill bacteria without affecting the body's normal microbiota [9, 10, 11]. However, concerns remain regarding its biosafety. The self‐replicating nature of phages can lead to unpredictable pharmacokinetics and pharmacodynamics, and may cause excessive immune activation or abnormal inflammation [12, 13]. Non‐lytic phages offer a safer option, as they do not induce bacterial lysis. Furthermore, loading therapeutic nanomaterials onto phages can enhance their efficacy [14, 15, 16]. However, so far, none of the reported studies screened biofilm‐binding peptides for biofilm eradication. Moreover, in biofilm infections, bacteria are often shielded within thick EPS layers [17, 18], limiting the ability of phages to effectively target and treat biofilm infections. To fill this gap, we proposed to discover biofilm‐binding phages (BBP) for eradicating biofilms.

**SCHEME 1:**
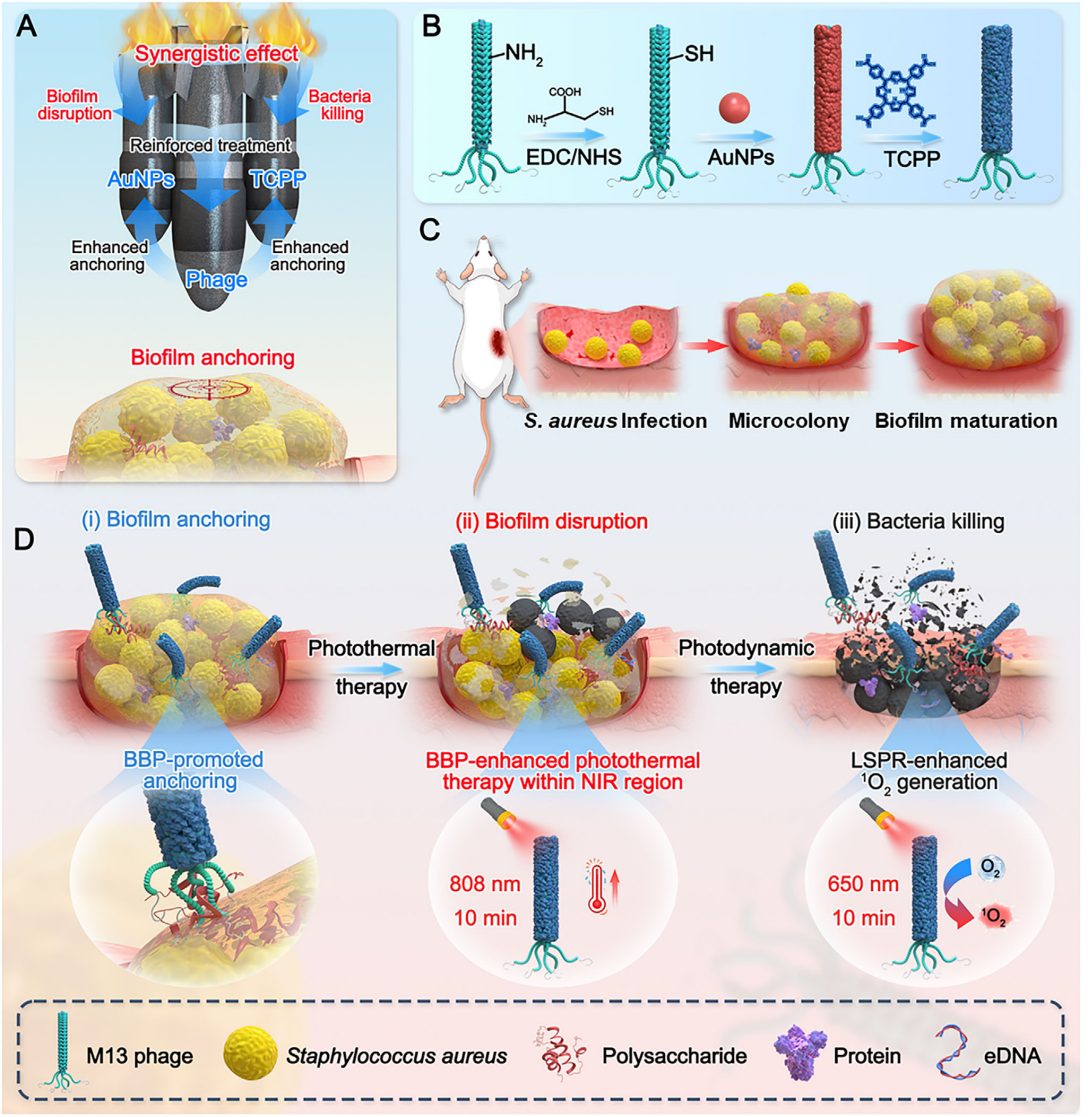
Schematic illustration of the assembly of the ternary synergetic system and the process of biofilm binding/anchoring, disassembly, and eradication. (A) The process of each element reinforces each other in a ternary synergetic system. (B) Fabrication of the ternary synergetic system. The BBP were first conjugated with cysteine through EDC‐NHS coupling, resulting in the formation of BBP‐SH. BBP‐SH were further loaded with AuNPs through Au─S bond, resulting in BBP/AuNPs. The latter were then decorated with TCPP through coordination interaction and hydrogen bonding, generating BBP/AuNPs/TCPP. (C) The formation of biofilm in mice wounds. (D) The process of biofilm anchoring, disassembly, and eradication. (i) BBPs efficiently bind to and anchor themselves within biofilms (mainly polysaccharides), allowing the trifunctional nanofibers to anchor onto the surface of biofilms. (ii) The aggregation of AuNPs on the BBP surface enables PTT within the NIR region. Upon irradiation at 808 nm for 10 min, the biofilms were disrupted via AuNPs‐induced PTT. (iii) Following PTT, the LSPR effect of AuNPs enhanced the ROS generation by TCPP through PDT. Under irradiation at 650 nm for 10 min, the ROS were generated and penetrated the disrupted biofilm, leading to further bacterial death.

**FIGURE 1:**
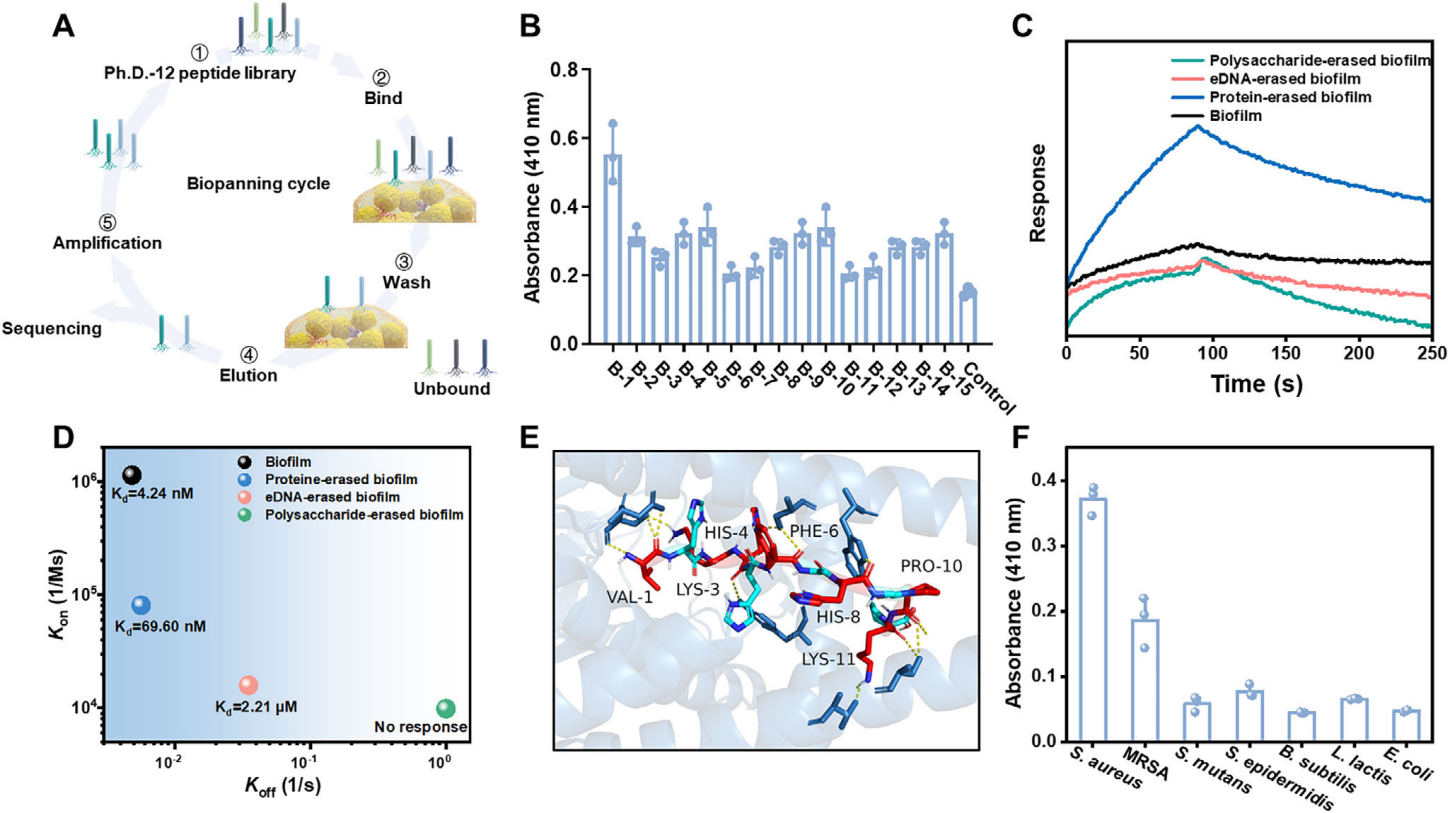
Selection for the BBP. (A) Biopanning procedure for the selection of *S. aureus* biofilm binding phages from Ph.D.‐12 peptide library. (B) Biofilm binding affinity of the screened phages via phage ELISA. (C) The association and dissociation kinetics of phage B‐1 (BBP) on the *S. aureus* biofilm modified BLI sensor surfaces before and after the biofilms were treated to eliminate eDNA, polysaccharides, or proteins. (D) The binding affinity of BBP against *S. aureus* biofilm before and after elimination of eDNA, polysaccharides, or proteins. (E) A possible binding model of BBP with PIA via molecular semi‐flexible docking. (F) The binding selectivity of BBP toward different types of biofilms.

In this work, we developed a ternary synergetic strategy for achieving reinforced ability of anchoring, disassembly, and eradication of biofilms. M13 phages were used both as warheads to bind and destroy biofilms and as scaffolds to arm two types of phototherapy agents for efficient biofilm elimination (Scheme 1A,B). In order to enhance the anchoring ability toward biofilm, M13 phages specifically binding *Staphylococcus* *aureus* (*S. aureus*) biofilm instead of planktonic *S. aureus* were screened by biopanning (Figure 1A). These phages were then armed with AuNPs and the photosensitizer TCPP (tetrakis(4‐carboxyphenyl) porphyrin). Under 808 nm irradiation, AuNP aggregates on the phages induced photothermal therapy (PTT), while TCPP generated singlet oxygen (^1^O_2_) under 650 nm light to trigger photodynamic therapy (PDT). The mild hyperthermia from AuNPs disrupted the biofilm architecture and enhanced ^1^O_2_ penetration. This synergy between the photothermal and photodynamic effects ultimately led to biofilm disassembly and bacterial killing (Scheme 1D).

In this ternary synergetic system, each two of the elements promote each other in terms of bacterial eradication and anti‐biofilm ability (Scheme 1A). (1) The phage endows the two types of phototherapy agents with the biofilm binding/anchoring ability, whereas the latter in turn reinforces the bacterial eradication and anti‐biofilm ability of phages; (2) The phages provide scaffolds for assembling AuNPs into fibrous structures, leading to a new optical absorption peak in the near‐infrared region (NIR), thereby enhancing the photothermal efficiency within the NIR window; (3) The localized surface plasmon resonances (LSPR) effect of AuNPs induces increase in the excitation efficiency of the accompanying TCPP, leading to a significant enhancement in the ^1^O_2_ generation. (4) The local hyperthermia produced by AuNPs promotes the cracking of biofilm and facilitates the subsequent photodynamic sterilization. The mild hyperthermia and sufficient ^1^O_2_ act synergistically to impart excellent antibiofilm efficiency.

## 2 Results and Discussion

### 2.1 Selection for the BBP

Naturally evolved phages exhibit stringent specificity toward its host bacteria. However, these phages may not be optimal candidates for treating bacterial biofilm infections, as the dense EPS coating can hinder the phages’ ability to directly attack the bacteria [18, 19]. To address this, we aimed to screen phages that specifically bind to biofilms from a Ph.D.‐12 peptide library (Figure 1A). After three rounds of biopanning against *S. aureus* biofilm, the phage recovery ratio increased, indicating valid selection (Figure S1). Sequence analysis of 20 randomly picked monoclonal phages revealed no matches with known proteins in the BLAST database (Table S1). The analysis also showed a significant enrichment of histidine residues in the peptide sequences of the phages selected against *S. aureus* biofilms (Figure S2). The high frequency of histidine may stem from its unique advantages in molecular interactions, as it exists in both neutral and protonated forms, facilitating a variety of interactions—such as cation–‐π, π–π stacking, and hydrogen bonding [20, 21]. The biofilm binding affinity of these monoclonal phages was further evaluated using phage enzyme‐linked immunosorbent assay (Phage‐ELISA). As shown in Figure 1B, phage B‐1, which carries a 12‐mer peptide at the N‐terminal of the pIII protein (amino acid sequence: VHKHHFGHGPKP), exhibited the highest binding capacity toward biofilms. Additionally, we screened phages that bind to planktonic *S. aureus* from the same peptide library and identified several *S. aureus*‐binding phages. Phage‐ELISA results revealed that phage B‐1 demonstrated a significantly higher biofilm binding ability compared to those *S. aureus*‐binding phages (Figure S3), thereby confirming our hypothesis that the phage B‐1, termed BBP, can be employed as a biofilm‐binding and anchoring agent.

Next, we investigated the binding sites between *S. aureus* biofilm and phage B‐1 using biolayer interferometry (BLI). BBP demonstrated an apparent dissociation constant (*K*_d_) of 4.24 nM against *S. aureus* biofilm (Figures 1C,D), indicating a high binding affinity. To identify the key components involved in phage B‐1 binding, we compared the apparent *K*_d_ values of BBP against biofilms with erased extracellular DNA (eDNA), polysaccharides, and proteins [22]. The results showed that the binding affinity was completely lost after removing polysaccharides, suggesting that polysaccharides in the biofilms may be the primary biofilm binding/anchoring sites for BBP. (Figure 1C,D; Figures S4 and S5). In addition, the removal of eDNA significantly reduced the binding affinity, suggesting a contributing role of eDNA in biofilm binding. To further explore the interaction between the BBP and polysaccharides, molecular semi‐flexible docking was employed to validate the potential binding model. Poly‐*N*‐acetylglucosamine (PIA, polysaccharide intercellular adhesin), the most prevalent polysaccharide in *S. aureus* biofilms [23, 24], was chosen for the modeling. As illustrated in Figure 1E and Table S2, multiple amino acids in the peptide were found to bind PIA through several hydrogen bonds, suggesting a potential strong and specific interaction. A comparison of binding affinities toward different types of biofilms (Figure 1F) showed that BBP exhibits good selectivity for *S. aureus* biofilm, with some binding ability also observed for methicillin‐resistant *S. aureus* (MRSA) biofilm. These observations demonstrated that BBP holds great potential as a tool for selective biofilm binding and anchoring.

### 2.2 Fabrication and Characterization of BBP‐Based Trifunctional Nanofibers

**FIGURE 2:**
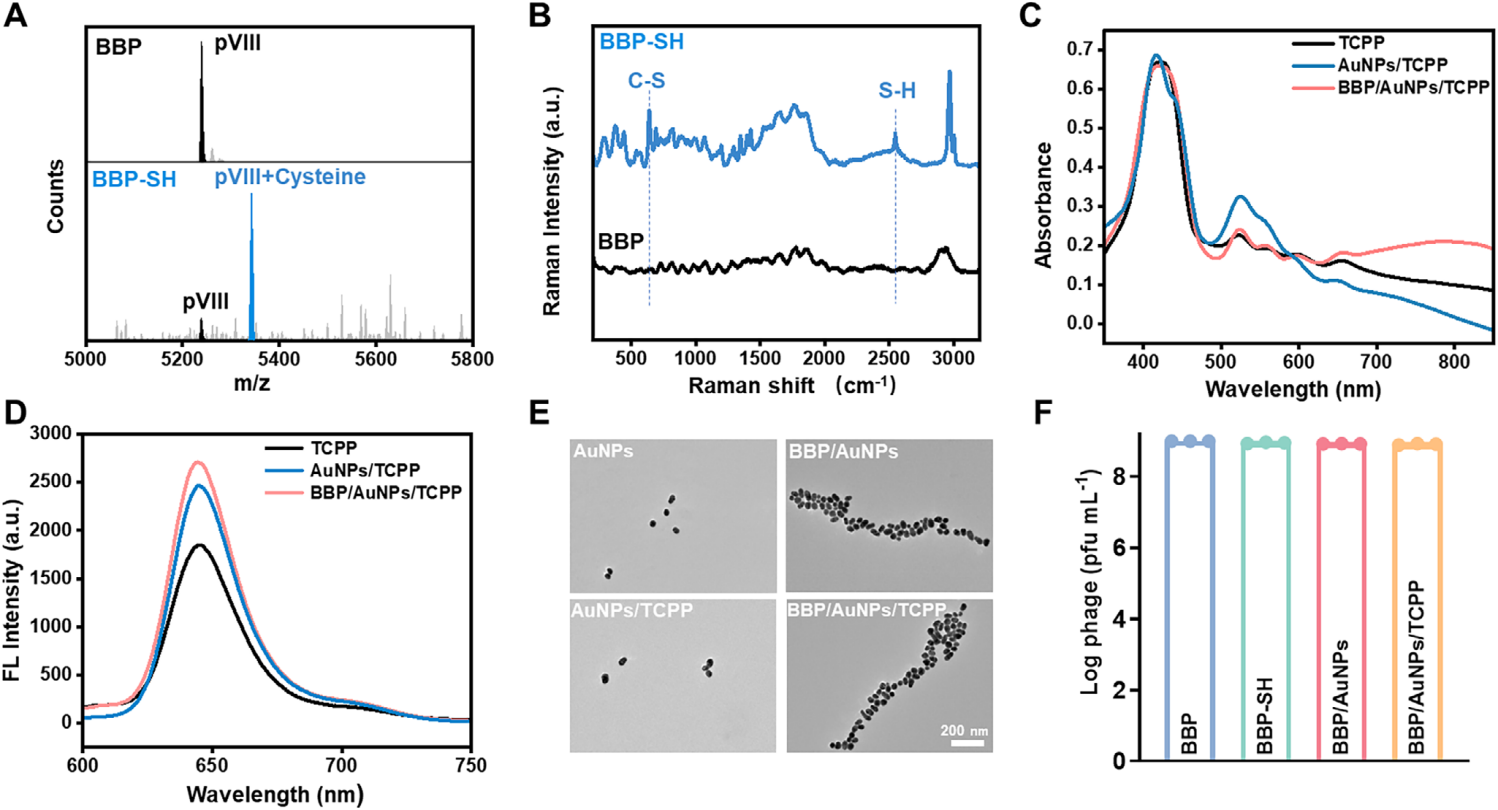
Characterization of BBP‐based trifunctional nanofibers. (A) The Q‐TOF LC/MS spectra of BBP and BBP‐SH. (B) The Raman spectra of BBP and BBP‐SH. (C) The UV–vis absorption spectra and (D) the fluorescence spectra of TCPP, AuNPs/TCPP, and BBP/AuNPs/TCPP. (E) TEM image of AuNPs, BBP‐SH loaded with AuNPs (BBP/AuNPs), AuNPs/TCPP, and BBP/AuNPs/TCPP. (F) The biofilm binding ability of BBP, BBP‐SH, BBP/AuNPs, and BBP/AuNPs/TCPP indicated by the retained phages on the *S. aureus* biofilm.

With BBP demonstrating the best binding affinity, we further assembled AuNPs onto BBP, followed by conjugation of TCPP to construct trifunctional nanofibers, BBP/AuNPs/TCPP (Scheme 1B). Briefly, the BBP were first conjugated with cysteine through EDC‐NHS coupling, resulting in the formation of BBP‐SH [25]. Q‐TOF LC/MS analysis confirmed the conversion of the major coat protein (pVIII) of BBP (5238 *m*/*z*) to pVIII‐cysteine (5341 *m*/*z*) after cysteine functionalization (Figure 2A). Raman spectroscopy further verified the successful construction of BBP‐SH, showing the presence of S─H stretching band at 2550 cm^−1^ and the C─S stretching vibration at 640 cm^−1^(Figure 2B). 30‐nm AuNPs, prepared by the sodium citrate reduction method, were subsequently attached to BBP‐SH through Au─S bonds, leading to the emergence of a new optical absorption peak in the NIR region (Figures S6 and S7). TCPP was further decorated onto the surface of AuNPs through coordination interactions and hydrogen bonding, resulting in an additional absorption band at 415 nm (Figure 2C). The presence of AuNPs also enhanced the fluorescence emission of TCPP at 645 nm (Figure 2D), further confirming the coupling of TCPP [26]. The change in zeta potential also indicated the successful fabrication of the trifunctional nanofibers, BBP/AuNPs/TCPP (Figures S8 and S9). TEM images of the BBP/AuNPs/TCPP revealed that the AuNPs were aligned along the fibrous structures of the phages (Figures 2E and S10). When comparing the biofilm binding ability of BBP/AuNPs/TCPP with that of BBP or BBP‐SH, we confirmed that the decoration of AuNPs/TCPP did not affect the biofilm binding ability of the phage (Figure 2F).

### 2.3 Performance of the BBP‐Based Trifunctional Nanofibers

**FIGURE 3:**
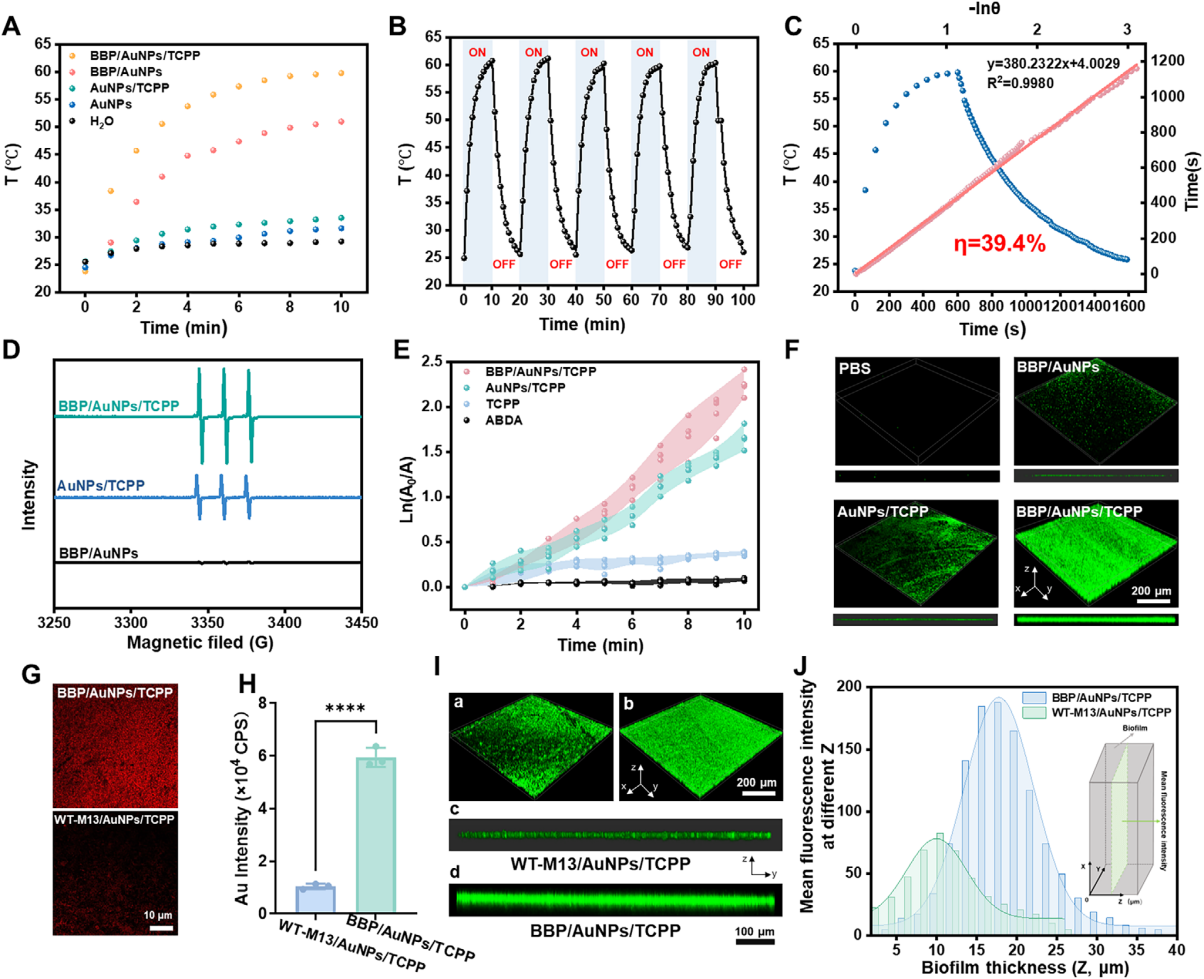
Performance of BBP‐based trifunctional nanofibers. (A) Temperature changes of AuNPs, AuNPs/TCPP, BBP/AuNPs, and BBP/AuNPs/TCPP under 808 nm irradiation with an intensity of 1.0 W cm^−2^ for 10 min. (B) The photothermal stability of BBP/AuNPs/TCPP within five heating/cooling cycles under 808 nm irradiation (1.0 W cm^−2^, 10 min). (C) Heating and cooling curves of BBP/AuNPs/TCPP upon 808 nm irradiation (1.0 W cm^−2^, 10 min) and the linear relationship between cooling time and [−ln (A_0_/A)] calculated from the cooling stage of BBP/AuNPs/TCPP. (D) EPR spectra of AuNPs/TCPP, BBP/AuNPs, and BBP/AuNPs/TCPP under 650 nm irradiation with an intensity of 0.5 W cm^−2^ for 10 min. (E) Photo‐oxidation rates of ABDA in the presence of TCPP, AuNPs/TCPP, and BBP/AuNPs/TCPP under light irradiation (808 nm for PTT+650 nm for PDT). (F) 3D CLSM images of *S. aureus* biofilms stained by DCFH‐DA probe after various treatments. (G) Fluorescence images of *S. aureus* biofilms after being treated with WT‐M13/AuNPs/TCPP and BBP/AuNPs/TCPP. The fluorescence is emitted from TCPP. (H) Amount of Au adhered to *S. aureus* biofilms treated with WT‐M13/AuNPs/TCPP and BBP/AuNPs/TCPP. (I) The x‐y‐z 3D CLSM images of *S. aureus* biofilms stained by DCFH‐DA probe after being treated with (a) WT‐M13/AuNPs/TCPP and (b) BBP/AuNPs/TCPP. The 2D CLSM projections of y‐z planes of the biofilms treated with (c) WT‐M13/AuNPs/TCPP and (d) BBP/AuNPs/TCPP are also shown to highlight the fluorescence signal along the thickness direction (z). (J) The average fluorescence intensity of the biofilms at different z values derived from the CLSM data in I, showing the diffusion distance of ^1^O_2_ within the biofilms.

We first evaluated the photothermal performance of BBP/AuNPs/TCPP. While AuNP aggregates exhibit good photothermal properties, external UV light irradiation is typically required, which may cause phototoxicity to cells and tissues. The incorporation of AuNPs along the phage structure introduces a new optical absorption band in the NIR region, thereby enhancing photothermal efficiency within the NIR window and minimizing phototoxicity. As shown in Figures 3A and S11, under 808 nm irradiation, both BBP/AuNPs/TCPP and BBP/AuNPs exhibited a rapid increase in temperature, whereas AuNPs or AuNPs/TCPP alone showed minimal photothermal ability under NIR irradiation. As porphyrin also possesses photothermal properties [27, 28], the photothermal performance of BBP/AuNPs/TCPP is relatively better than that of BBP/AuNPs. Additionally, BBP/AuNPs/TCPP demonstrated excellent photothermal reversibility and stability, as evidenced by the unaltered performance after multiple irradiation cycles (Figures 3B and S12). Based on the heating‐cooling curve and the corresponding thermal time constant (τ_S_) (Figure 3C), the photothermal conversion efficiency (*η*) of BBP/AuNPs/TCPP was ascertained to be 39.4%, confirming the effectiveness of BBP/AuNPs/TCPP in converting NIR light energy into heat.

We then assessed the ROS generation of BBP/AuNPs/TCPP using electron paramagnetic resonance (EPR). As shown in Figures 3D and S13, both BBP/AuNPs/TCPP and AuNPs/TCPP generated singlet oxygen (^1^O_2_) under 650 nm irradiation, but no hydroxyl radicals (•OH) or superoxide anions (•O_2_^−^) were detected. We also quantitatively evaluated the ^1^O_2_ generation capability of the BBP/AuNPs/TCPP using the 9,10‐anthracenediyl‐bis(methylene)dimalonic acid (ABDA) assay. As shown in Figures 3E and S14, the ^1^O_2_ generation capability of free TCPP was the lowest, likely due to its weak photoexcitation efficiency. When TCPP was attached to AuNPs, the LSPR effect of the AuNPs enhanced the excitation efficiency of the TCPP [26, 29], resulting in a significant increase in ^1^O_2_ generation. Specifically, AuNPs/TCPP exhibited a 3.6‐fold higher ^1^O_2_ generation capability compared to free TCPP. Furthermore, the assembly of AuNPs/TCPP onto BBP further increased the particle size, and BBP/AuNPs/TCPP demonstrated a 4.2‐fold higher ^1^O_2_ generation capability compared to free TCPP [30]. This observation highlights the potential of BBP/AuNPs/TCPP for PDT. The ^1^O_2_ produced in *S. aureus* biofilm after treatment with BBP/AuNPs/TCPP was further visualized using the fluorescent probe DCFH‐DA. As shown in Figures 3F and S15, after treatment with BBP/AuNPs/TCPP under light irradiation (808 nm for PTT and 650 nm for PDT), the *S. aureus* biofilm exhibited intense green fluorescence, confirming the substantial generation of ^1^O_2_. In contrast, treatment with AuNPs/TCPP or BBP/AuNPs resulted in only a small amount of ^1^O_2_ generation.

We further confirmed the biofilm‐binding ability of BBP/AuNPs/TCPP by incubating *S. aureus* biofilm with either BBP/AuNPs/TCPP or WT‐M13/AuNPs/TCPP (WT‐M13 refers to the wild‐type M13 phage, which lacks biofilm‐binding ability), followed by rinsing to remove any loosely bound materials from the biofilm surface. As shown in Figure 3G, due to the lack of biofilm‐binding capability, WT‐M13/AuNPs/TCPP showed minimal retention on the biofilm. In contrast, the BBP/AuNPs/TCPP‐treated biofilm exhibited bright fluorescence from TCPP, indicating that a significant amount of the nanofiber remained on the biofilm surface, owing to the biofilm‐binding ability of the BBP. We also quantified the gold (Au) intensity in the biofilms using ICP‐MS, and found that the Au intensity in the BBP/AuNPs/TCPP‐treated biofilm was 6 times higher than that in the WT‐M13/AuNPs/TCPP‐treated biofilm, further confirming the contribution of the BBP in binding to the biofilms (Figure 3H). The biofilm‐binding ability of the BBP also enhanced the biofilm penetration efficiency of ^1^O_2_, as evidenced by the 3D CLSM images (Figures 3I,J), which showed significantly deeper penetration of ^1^O_2_ in the biofilm treated with BBP/AuNPs/TCPP than in the biofilm treated with WT‐M13/AuNPs/TCPP. This observation underscores the critical role of the phages in promoting biofilm interaction and enhancing the permeability of ^1^O_2_. Therefore, BBP‐based trifunctional nanofibers are expected to effectively eliminate bacteria and disrupt biofilms by a combination of biofilm‐anchored PTT and PDT.

### 2.4 Antibacterial Activity of the BBP‐Based Trifunctional Nanofibers In Vitro

**FIGURE 4:**
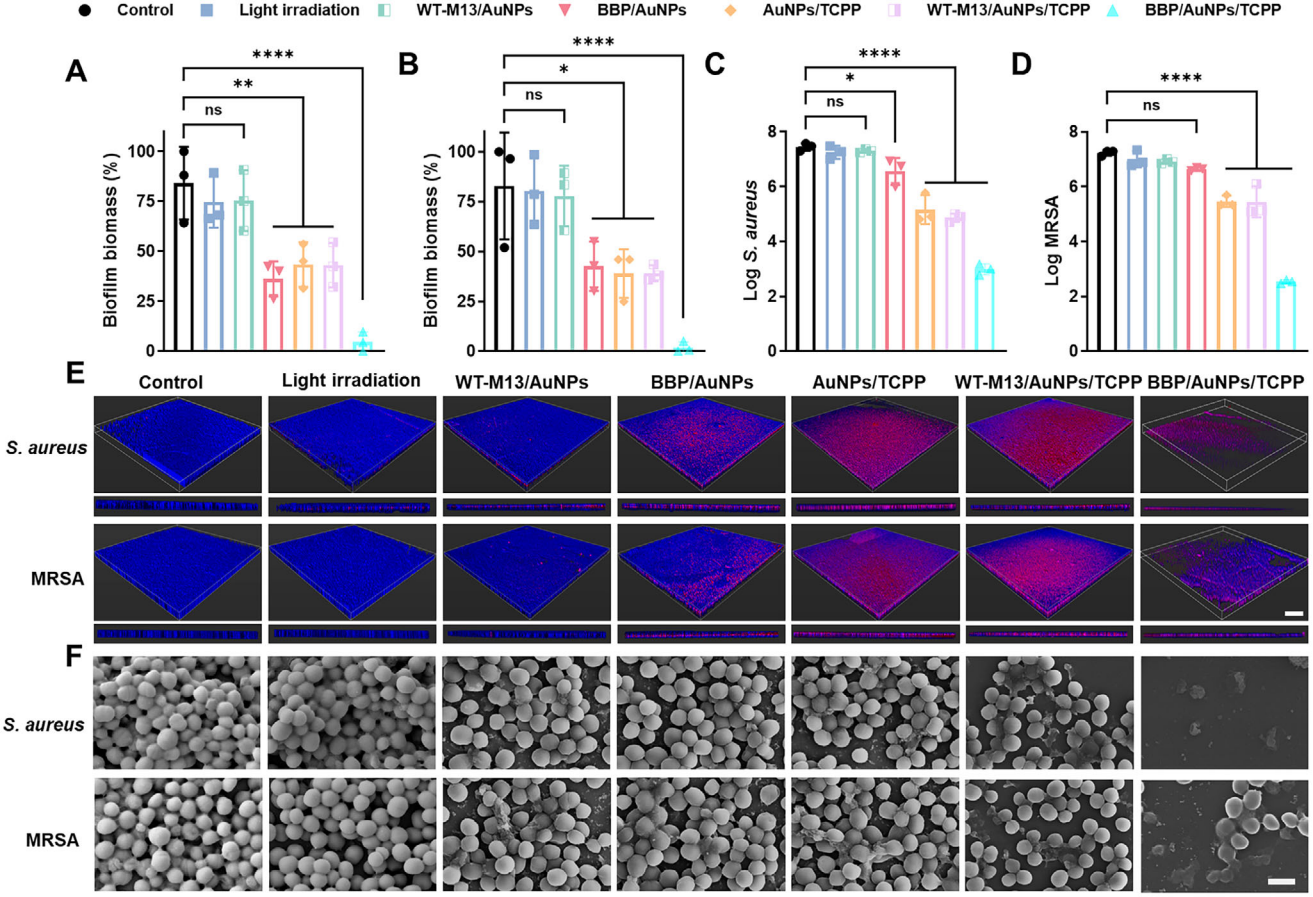
In vitro evaluation of antibiofilm activity. (A,B) Biomass of *S. aureus* and MRSA biofilms after various treatments. (C,D) Numbers of viable bacteria cells within *S. aureus* and MRSA biofilms after various treatments. (E) 3D CLSM images of *S. aureus* biofilms and MRSA biofilms stained by Hoechst 33342 /PI after various treatments. Scale bar is 200 µm. (F) SEM images of *S. aureus* biofilms and MRSA biofilms after various treatments. Scale bar is 1 µm.

We first assessed the biomass amount of *S. aureus* biofilm and MRSA biofilm after different treatments using a crystal violet staining assay. As shown in Figures 4A,B and S16, both PTT (BBP/AuNPs) and PDT (AuNPs/TCPP) induced a moderate reduction in biofilm biomass; the WT‐M13/AuNPs/TCPP showed limited efficacy due to its lack of binding, whereas the ternary synergistic system (BBP/AuNPs/TCPP) resulted in the most significant decrease in biofilm biomass due to a combination of PTT and PDT. The remaining viable bacteria in the biofilm after different treatments were quantified using the plate counting method. As shown in Figures 4C,D and S17, the bactericidal effect of PTT (BBP/AuNPs) alone was not significant, with only a one‐order magnitude reduction in bacterial count. This limited efficacy can be attributed to the increased heat tolerance of bacteria due to the protective bacterial cell wall and the dense structure of the biofilm, which hindered the bactericidal effect. In contrast, AuNPs/TCPP and WT‐M13/AuNPs/TCPP showed a moderate bactericidal effect against *S. aureus* biofilm, but exhibited poor performance against MRSA biofilm. After treatment with BBP/AuNPs/TCPP, the bacterial count in both *S. aureus* and MRSA biofilms was reduced by four orders of magnitude, demonstrating the excellent antibiofilm activity of BBP/AuNPs/TCPP.

Furthermore, the treatment of BBP/AuNPs/TCPP led to a significant increase in extracellular protein, indicative of severe membrane disruption and cytoplasmic leakage. Correspondingly, extracellular ATP levels dropped markedly under the same conditions (Figure S18). For comparison, we also evaluated the bactericidal performance of several traditional antibiotics (Vancomycin and Oxacillin). As expected, these antibiotics showed limited or no effect on *S. aureus* or MRSA biofilms (Figure S19), primarily due to the protective thick EPS layer, which shields the bacteria within the biofilm. This highlights the urgent need for the development of more effective antibiofilm agents. Additionally, the antibiofilm effect was visualized using live/dead staining (Hoechst 33342/PI). As shown in Figure 4E, only blue fluorescence from Hoechst 33342 was observed in the Control (no treatment), light irradiation, and WT‐M13/AuNPs groups, indicating that the bacteria were alive. However, after treatment with BBP/AuNPs/TCPP, significant damage to the biofilm structure was observed, with a noticeable reduction in biofilm thickness. Furthermore, bright red fluorescence was also detected, indicating that nearly all the bacteria within the biofilm were killed.

To further understand how BBP/AuNPs/TCPP eradicates biofilms, the morphologies of biofilms subjected to different treatments were investigated via scanning electron microscopy (SEM). As shown in Figure 4F, the control and light irradiation group displayed dense 3D biofilm morphology with preserved morphology. When treated with AuNPs/TCPP and BBP/AuNPs, biofilm exhibited slightly looser, with most bacteria maintaining the structural integrity. In contrast, after treatment with BBP/AuNPs/TCPP, there was a significant reduction in the number of bacteria in both *S. aureus* and MRSA biofilms, and the structure of the biofilm completely disappeared. (Figure 4F).

These results quantitatively validate the synergistic antibiofilm activity between PTT and PDT when spatially organized by the biofilm‑binding phage scaffold. Using the Bliss independence model, we confirmed that the observed sterilization rate of the ordered BBP/AuNPs/TCPP assembly was significantly higher than the theoretically expected additive effect (Figure S20A), with a positive interaction value (Δ = 19.34%) indicating a genuine synergistic interaction rather than a simple superposition of individual effects. Control experiments further demonstrated that the ordered assembly exhibited a markedly superior therapeutic efficacy compared with both the non‑assembled physical mixture and the simple mixture of functional units (Figure S20B), confirming that the enhanced antibacterial activity was driven by BBP‑mediated spatial organization rather than mere co‑delivery of the therapeutic components. Collectively, it highlights the critical role of the biofilm‑binding phage as a versatile scaffold to precisely arrange phototherapeutic agents, thereby maximizing the cooperative effects of PTT and PDT and achieving significantly improved antibacterial performance.

### 2.5 Treatment of Biofilm‐Infected Wounds

Before carrying out the in vivo antibacterial study, the cytocompatibility of BBP‐based trifunctional nanofibers at different concentrations was evaluated in vitro by incubating RAW264.7 cells with BBP/AuNPs, AuNPs/TCPP, and BBP/AuNPs/TCPP, with or without light irradiation. Cell viability in RAW264.7 cells remained above 90% at concentrations below 0.5 mg mL^−1^ (Figure S21), and light irradiation did not affect cell viability. Hemolysis assays performed on peripheral blood showed less than 5% hemolysis for the BBP/AuNPs and BBP/AuNPs/TCPP groups at 0.5 mg mL^−1^, confirming their biosafety (Figure S22). To further evaluate the antibiofilm ability of BBP/AuNPs/TCPP in vivo, we established a skin wound model (1.0 cm in diameter) with *S. aureus* infection on the dorsal side of BALB/c mice. As shown in Figure S23A,B, the wound was covered with yellow purulent secretions, and bacterial colonies in the wound reached 10^8^ CFU mL^−1^ on Day 3. Wound blotting and H&E staining confirmed the successful establishment of a biofilm infection model [32] (Figure S23A,C).

**FIGURE 5:**
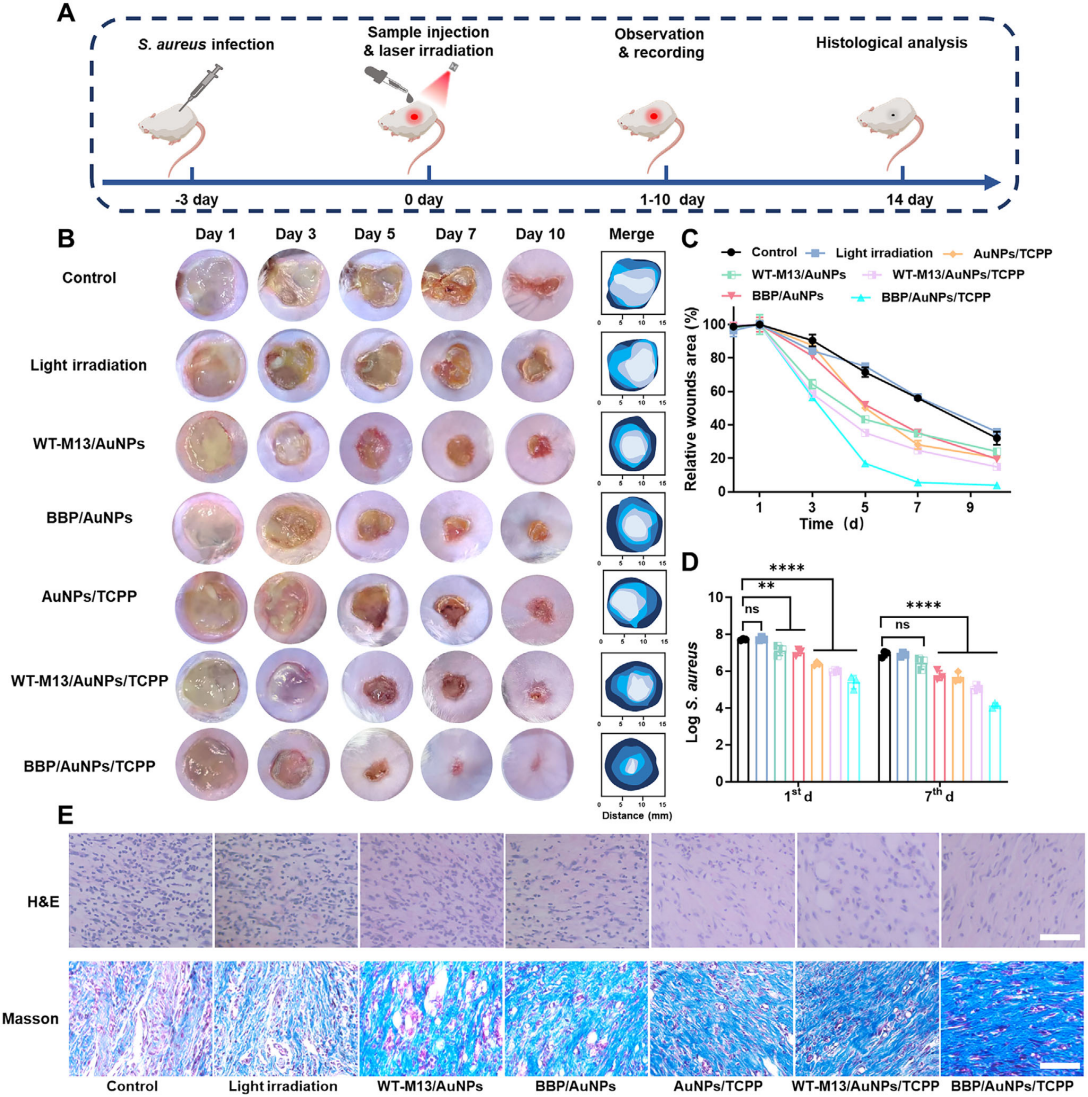
The therapeutic effects of BBP/AuNPs/TCPP to achieve the in vivo treatment of the *S. aureus* biofilm. (A) Schematic illustration of the in vivo treatment of the *S. aureus* biofilm. (B) Photographs of wound healing conditions during 10‐day treatment by different ways, along with the overlaid images. (C) Corresponding quantitative evaluation of the relative wound area over time. (D) Statistical analysis of bacterial colonies obtained from the wound tissues after various treatments. (E) H&E and Masson staining of the peripheral tissue after various treatments on the 14th day. Scale bar is 50 µm.

The biofilm‐infected wounds were then randomly divided into seven treatment groups, following the therapeutic procedure outlined in Figure 5A. Photographs of the wounds and the corresponding quantitative analysis of the wound area are shown in Figure 5B. The infected wounds in all treatment groups gradually healed over time, but the extent of infection and the healing rate varied significantly (Figure 5C). With BBP/AuNPs/TCPP treatment (Day 0), the wound temperature rapidly increased to ∼46°C during laser irradiation (Figure S24). The mild hyperthermia potentiated the efficacy of the ^1^O_2_ by enhancing its penetration into the biofilm and compromising bacterial viability. Furthermore, the localized heating might promote the innate immune response by increasing lymphocyte export, blood flow, and local oxygen tension [33, 34]. On Day 7, a pronounced reduction in wound area was observed in the BBP/AuNPs/TCPP group, followed by essentially complete healing by Day 10. Comparing the colony counts of wound tissue on Day 1 and Day 7, the BBP/AuNPs/TCPP group exhibited the highest antibacterial efficacy, with bacterial numbers reduced by three orders of magnitude (Figures 5D and S25). This improved efficacy is consistent with the strong biofilm binding and enhanced local retention observed in vitro (rinse‐off assay and Au quantification), together with synergistic PTT/PDT. To compare the efficacy of BBP/AuNPs/TCPP treatment with standard therapy, we assessed the topical antibiotic mupirocin ointment using the same wound infection model. As shown in Figure S26, the standard therapy exhibited similar bactericidal efficacy to BBP/AuNPs/TCPP treatment, but wound healing was slower compared to the BBP/AuNPs/TCPP group.

Hematoxylin and eosin (H&E) staining was further used to assess histopathological changes on Day 14 (Figure 5E). In the control group, a few neutrophils were still present, indicating ongoing inflammation at the wound site. In contrast, the experimental groups exhibited fewer inflammatory cells, with increased collagen deposition that was evenly distributed and dense, indicating that the infection was nearly cured. This reduced inflammatory infiltration is consistent with inflammation resolution following effective bacterial clearance. Furthermore, H&E staining of major organs (heart, liver, spleen, lung, and kidney) showed no signs of lesions (Figures S27 and S28), and the body weights of the mice remained similar across different treatment groups (Figure S29), demonstrating the excellent histocompatibility of the BBP‐based trifunctional nanofibers.

### 2.6 Antibacterial Mechanism of the BBP‐Based Trifunctional Nanofibers

ROS and thermal effects have been shown to disrupt biofilms by inactivating key components of the biofilm matrix, such as eDNA and proteins [35]. As a major component of the EPS, eDNA acts as a “glue,” integrating the various elements of the bacteria into the cohesive structure of the biofilm, thus playing a crucial role in bacterial adhesion and biofilm stabilization [3, 36]. To further explore the biofilm disruption mechanism of BBP/AuNPs/TCPP, its ability to degrade eDNA was assessed using agarose gel electrophoresis (AGE). As demonstrated in Figure S34A, after treatment with AuNPs/TCPP or BBP/AuNPs/TCPP, the prominent DNA bands corresponding to eDNA disappeared, which was consistent with the quantitative results shown in Figure S34B. These results suggest that the ROS/thermal microenvironment generated by the phototherapeutic system can impair eDNA, which is expected to weaken EPS cohesion and facilitate biofilm destabilization.

**FIGURE 6:**
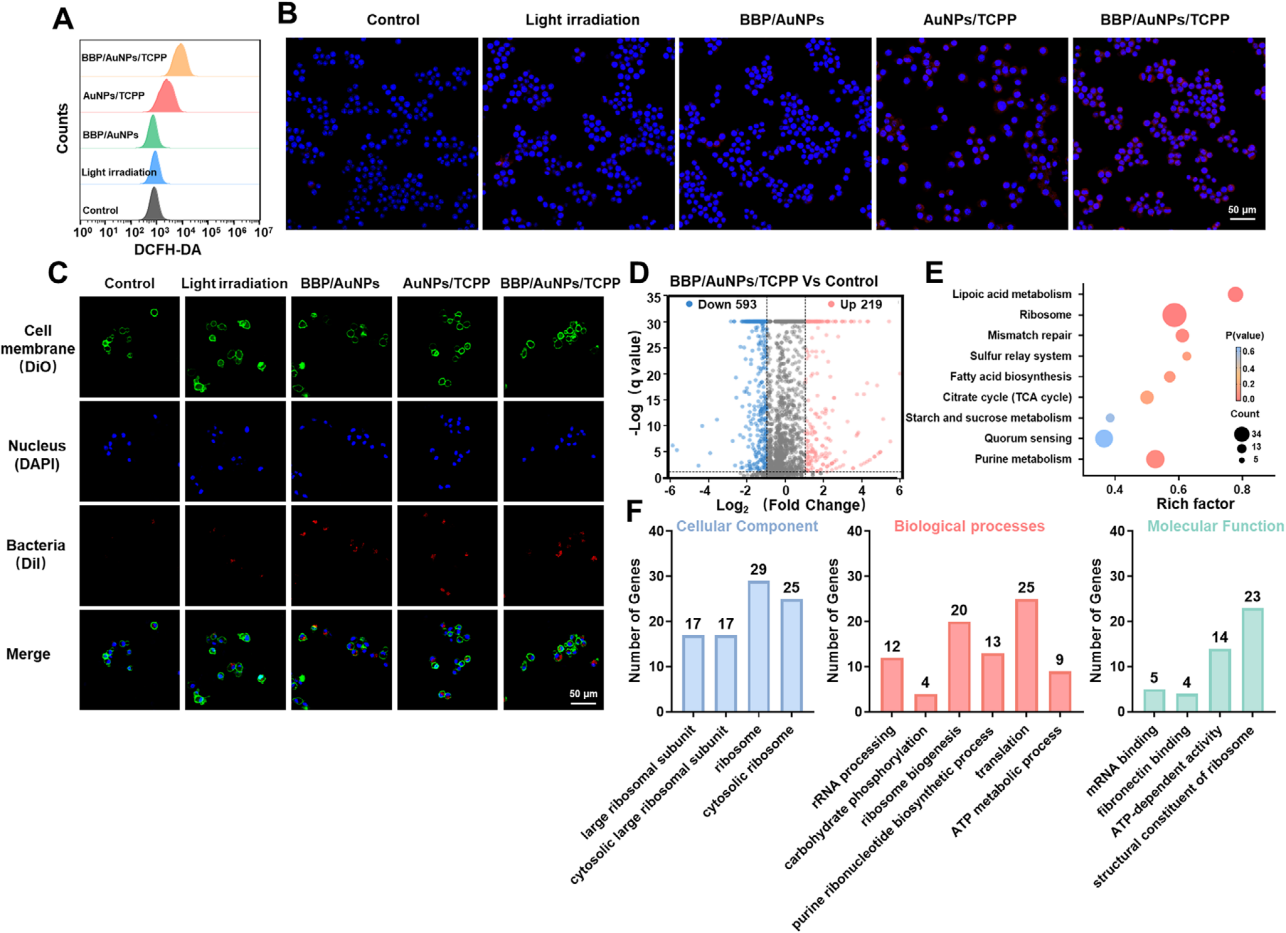
In vitro macrophage assays and bacterial transcriptomic analysis after BBP/AuNPs/TCPP treatment. (A) The intracellular ROS level of RAW264.7 cells after various treatments. (B) CD86 expression on RAW264.7 cells after various treatments. Blue fluorescence represents RAW264.7 cell nuclei, and red fluorescence indicates CD86 protein. (C) CLSM images showing macrophage phagocytosis of *S. aureus* after the indicated treatments (green: RAW264.7 membrane; blue: nuclei; red: *S. aureus*). Scale bar: 50 µm. (D) Volcano plot of differentially expressed genes (DEGs) in *S. aureus* biofilms treated with BBP/AuNPs/TCPP compared to the control. The vertical dashed lines indicate the threshold of |log_2_(fold change)| ≥ 1, and the horizontal dashed line corresponds to the threshold: −log_10_(*q* value) ≥ −log_10_ (0.05). (blue: downregulated; red: upregulated; grey: non‐DEGs). (E) KEGG pathway enrichment/classification of DEGs identified in (D). (F) GO enrichment/classification of DEGs identified in (D) (Cellular component, biological process, and molecular function).

Beyond direct bactericidal action, phototherapies have also been increasingly recognized to modulate innate immune responses in the local microenvironment. In particular, transient ROS bursts and mild hyperthermia can serve as danger‐associated cues that enhance macrophage bactericidal functions and debris clearance, potentially complementing the initial phototherapeutic killing [37, 38, 39]. Motivated by this emerging framework, we further assessed macrophage responses to BBP/AuNPs/TCPP at the cellular level. We first quantified intracellular ROS in RAW 264.7 cells by flow cytometry. As shown in Figure 6A, AuNPs/TCPP and BBP/AuNPs/TCPP produced elevated ROS signals, consistent with the photosensitizer‐dependent ROS generation under 650 nm irradiation. We next evaluated whether this ROS microenvironment was associated with macrophage phenotype changes. As shown in Figures 6B and S35, compared to AuNPs/TCPP, BBP/AuNPs/TCPP induced a greater increase in CD86 expression, indicating enhanced polarization of macrophages toward the M1 phenotype. We further examined macrophage phagocytosis by CLSM. As shown in Figure 6C, macrophages treated with AuNPs/TCPP or BBP/AuNPs/TCPP exhibited higher uptake of *S. aureus* than other groups, and quantification of intracellular live bacteria supported this observation (Figures S37 and S38). Collectively, these data suggest that, in addition to direct PTT/PDT‐mediated killing, BBP/AuNPs/TCPP may enhance macrophage bactericidal functions in vitro, which could contribute to bacterial clearance and reduce the likelihood of biofilm re‐establishment.

To further analyze the molecular response mechanism of bacteria, the transcriptome analysis was conducted on *S. aureus* with PBS (Control) and [BBP/AuNPs/TCPP] treatment. As shown in Figure 6D, of nearly 3,000 genes, 219 genes were up‐regulated, and 593 genes were down‐regulated in the [BBP/AuNPs/TCPP] group compared with the control group, indicating a predominantly repressive effect of [BBP/AuNPs/TCPP] on the bacterial transcriptome. The gene ontology (GO) analysis indicated differential regulation of gene sets associated with ribosome assembly, protein translation, ATP‐energy metabolism, and adhesion‐related functions (Figure 6F). Furthermore, the Kyoto Encyclopedia of Genes and Genomes (KEGG) functional enrichment analysis further highlighted pathway‐level changes involving quorum sensing, ribosome‐related processes, membrane/transport functions, energy metabolism, and DNA repair‐associated programs (Figure 6E). These transcriptomic signatures are interpreted as a stress‐response profile consistent with severe oxidative/thermal injury, and align with the observed phenotypes of biofilm disruption and bacterial loss of viability.

## 3 Conclusion

This study introduces a synergistic nanofiber that combines M13 phage nanocarriers with PTT and PDT to effectively anchor and disrupt bacterial biofilms, which are often resistant to conventional treatments. The engineered M13 phage provides biofilm‐matrix anchoring and enhanced local retention at the biofilm interface under rinse‐off conditions, helping maintain an effective local dose for therapy. By co‐localizing AuNP‐based photothermal conversion (808 nm NIR) and TCPP‐based photodynamic action (650 nm red light) on the same nanofiber scaffold, the platform enables cooperative PTT/PDT antibiofilm activity, leading to matrix disruption and pronounced bacterial killing that cannot be explained by heating alone. The nanosystem demonstrates robust antibiofilm efficacy against *S. aureus* and MRSA in vitro and promotes recovery in a biofilm‐associated skin wound model with favorable biocompatibility and minimal systemic exposure after local administration. These results highlight matrix‐anchored, phage‐templated co‐localized dual phototherapy as a promising strategy for combating biofilm‐associated infections and provide a modular framework for further optimization and mechanistic refinement.

## 4 Experimental Section

### 4.1 Materials

Standard strains of *Escherichia coli* (*E. coli* O157:H7) and *Staphylococcus epidermidis* (*S. epidermidis*, CICC 116091) were purchased from the China Center of Industrial Culture Collection (Beijing, China). *Staphylococcus aureus* (*S. aureus*, CMCC 26003) and methicillin‐resistant *S. aureus* (MRSA, ATCC 43300) were received from BeNa Culture Collection (Beijing, China). Ph.D.‐12TM phage display peptide library kit, tetracycline‐resistant *Escherichia coli* (*E. coli ER2738*), and HRP‐conjugated anti‐M13 monoclonal antibody were obtained from NEB (New England Bio‐Labs, US). TIANprep Mini Plasmid Kit was purchased from Beijing Yunpeptide Biotechnology Co., Ltd. (Beijing, China). Primary antibodies (anti‐CD86, anti‐CD206) and secondary antibodies (Cy5‐IgG, FITC‐IgG) were purchased from Beijing Biosynthesis Biotechnology Co., Ltd. (Beijing, China). L‐cysteine, NaCl, KCl, NaOH, Na_2_HPO_4_·12H_2_O, NaH_2_PO_4,_ and HAuCl_4_·4H_2_O were provided by Sinopharm Chemical Reagent Co., Ltd. (Shanghai, China). Trisodium citrate dihydrate was bought from Yongda Chemical Reagent Co., Ltd. (Tianjin, China). BSA, 2‐morpholinoethanesulfonic acid (MES), 1‐ethyl‐3‐[3‐(dimethylamino)‐propyl] carbodiimide hydrochloride (EDC), N‐hydroxysuccinimide (NHS), tris (hydroxymethyl) aminomethane (Tris), oxacillin sodium, vancomycin hydrochloride, and 5,10,15,20 tetris (4‐carboxyphenyl) porphyrins (TCPP) were purchased from Aladdin Chemical Reagent Co., Ltd. (Shanghai, China). Methylthiazolyldiphenyl‐tetrazolium bromide (MTT), Hoechst 33342, propidium iodide (PI), hematoxylin‐eosin (HE) stain kit, and modified Masson's trichrome stain kit were bought from Solaibao Technology Co., Ltd. (Beijing, China). RAW264.7 cells (a mouse mononuclear macrophage leukemia cell line) were the products of Procell Life Science &Technology Co., Ltd. (Wuhan, China). BALB/c mice (8 weeks old, male) were bought from Liaoning Changsheng Biotechnology Technology Co., Ltd. (Benxi, China). Deionized water (DI water, 25°C) was used throughout.

### 4.2 Preparation of Mature Biofilms

The bacteria models (*E. coli O157:H7*, *E. coli ER2738*, and *S. epidermidis*) were inoculated in the LB medium. *S. aureus* was inoculated in the Broth medium. MRSA was inoculated in TSB medium (tryptic soy broth). The above bacteria were cultured at 37°C for 12 h, and the solutions were stored at 4°C in a refrigerator for further use. Afterward, 100 µL bacteria suspension (1 × 10^7^ CFU mL^−1^) was added into 96‐well plates. Then, the bacteria were cultured at 37°C for 48 h, and the culture media were refreshed every 24 h. Finally, the culture medium was removed, and the free bacteria were washed with PBS, the mature biofilm formed on the 96‐well plates was harvested for further analysis.

### 4.3 Phage Screening

Phage screening was performed using the Ph.D.‐12 peptide library kit. The mature *S. aureus* biofilm was washed three times by 1 × PBS. The 96‐well plate with mature biofilm was blocked with 5 mg mL^−1^ BSA and incubated at 4°C for 60 min. After washed by TBST (0.1% Tween‐20), the phage peptide library (10^11^ pfu mL^−1^, 100 µL) was added, incubated at room temperature for 60 min. After being washed by TBST, the phage was eluted with 0.2 M glycopine‐HCl, 1 mg mL^−1^ BSA. The elution was collected and neutralized with Tris‐HCl (1 M, pH 9.0). The eluted phages were propagated by infecting *E. coli ER2738* cell culture. The phages were then purified, concentrated, and quantified to proceed with the next round of biopanning. A total of three rounds of biopanning were conducted against the biofilm to isolate phages with high binding affinity. Finally, the negative biopanning against BSA was performed to further increase the specificity of the screened phages toward biofilm.

After that, well‐separated plaques were randomly selected for amplification, and the plasmid was extracted by TIANprep Mini Plasmid Kit for DNA sequencing by Sangon Biotech Co., Ltd. (Shanghai, China).

### 4.4 Phage‐Biofilm Interactions by Bio‐Layer Interferometry (BLI)

The binding affinity between phage and biofilm was measured by bio‐layer interferometry on an Octet K2 System (FortéBio, USA). Briefly, we employed an optimized static adsorption‐growth protocol. The sensor tip was immersed in a suspension of *S. aureus* (10^7^ CFU mL^−1^) for 24 h to allow for initial bacterial adhesion and fixation onto the sensor surface. Subsequently, the sensor with adhered bacteria was vertically transferred into fresh tryptic soy broth medium and incubated under static conditions at 37°C for 24 h to facilitate the development of the mature biofilm.

The biofilm‐sensors were exposed to phage solutions of various concentrations in TBS, followed by dissociation in TBS. Association rate constants (*K*_on_), dissociation rate constants (*K*_off_), and equilibrium dissociation constants (*K*_d_) were determined using the software provided by FortéBio. In detail, kinetics data were obtained by monitoring the binding response signal of the peptide‐modified biosensors at various phage concentrations (from 0.625 to 10 nM) in real time, to obtain on (*K*_on_) and off (*K*_off_) rates. The equilibrium binding constant (*K*_d_) was then calculated as *K*_off_ / *K*_on_ using a 1:1 binding stoichiometric model. A reference sensor was run with a blank assay buffer without phage for the association and dissociation steps, and reference subtraction was performed before data analysis, so that the background dissociation would be subtracted out in order to calculate accurate kinetic constants. All the binding constants were calculated automatically by the Octet Data Analysis software (Data Analysis 11.0, Octet) according to the binding kinetics data.

### 4.5 Preparation of BBP‐SH

The amine groups on the surface of M13 phages are highly reactive and can be chemically modified by EDC/NHS chemistry. Briefly, 0.1 mol L^−1^ cysteine, 1.1 mol L^−1^ EDC, and 2.5 mol L^−1^ NHS were dissolved in 0.1 mol L^−1^ MES buffer (pH 6.0) and incubated for 30 min at 37°C to activate cysteine. Afterward, 500 µL of activated cysteine solution was added to 100 µL BBP suspension (10^13^ pfu mL^−1^ in TBS). After 18‐h incubation at 4°C, the excessive reactants were removed through Amicon Ultra‐4 Centrifugal Filter Devices (100 KDa), the resultant BBP‐SH was collected and stored at 4°C for future use.

### 4.6 Fabrication of BBP‐Based Trifunctional Nanofibers

The synthesis of AuNPs was carried out by the citrate reduction method [40]. Briefly, 25 mL of 0.01% HAuCl_4_ (m/v) was heated to boiling along with vigorous stirring. Then, 350 µL sodium citrate (1%) was rapidly added into the boiled solution and continued heated for 10 min. 100 µL 1×10^12^ pfu mL^−1^ BBP‐SH was then added in 1 mL of the freshly prepared AuNPs solution and incubated at 37°C for 1 h, followed by 12000 rpm centrifugation for 5 min. The sediments were re‐suspended in sterile water, and then added with 10 µL 1 mM TCPP solution for overnight incubation at 37°C. After three cycles of centrifugation (12,000 rpm, 5 min) and washing, the precipitations were collected and re‐suspended in sterile water, and stored at 4°C for future use.

### 4.7 In Vitro Photothermal Effect Evaluation

In a typical experiment, 1 mL BBP/AuNPs/TCPP aqueous solution (0.5 mg mL^−1^) was irradiated under 808 nm laser (1.0 W cm^−2^) for 10 min. After that, the solution was allowed to naturally cool down to room temperature. The temperature of the solutions was continuously monitored with a temperature sensor. The photothermal conversion efficiency (*η*) was calculated by the following equation [37]:

(1) $$\eta =\frac{hA\left(T_{max}-T_{s}\right)-Q_{0}}{I\left(1-10^{-A_{808}}\right)}\;$$

where *h* is the heat transfer coefficient, *A* is the surface area of the container, *T*_max_ is the maximum temperature achieved, *T*_s_ is the surrounding temperature, *Q*_0_ represents the heat input due to light absorption by the solvent, *I* is the power density of the laser (1.0 W cm^−2^), and *A*_808_ is the absorbance of the sample at 808 nm (0.741).

*Q*_0_ and *hA* can be calculated by the following equation:

(2) $$\;Q_{0}=\frac{m_{H_{2}O}C_{H_{2}O}\left(T_{max,H_{2}O\;}-T_{s}\right)}{\tau_{H_{2}O}}$$

(3) $$\mathrm{hA}=\frac{\sum_{i}m_{i}C_{p,\;i}}{\tau_{s}}\;\approx \frac{m_{H_{2}O}C_{H_{2}O}}{\tau_{s}}$$

where $m_{H_{2}O}$ is the solution mass (1 g), $C_{H_{2}O}$ represents the heat capacity of water (4.2 J/g). During the natural cooling period, *τ_s_* can be derived from the linear regression curve of the following equation:

(4) $$t=-\tau_{s}\ln\theta =-\tau_{s}\ln\frac{T-T_{s}}{T_{\max}-T_{s}}$$

### 4.8 Detection of Singlet Oxygen (^1^O_2_)

^1^O_2_ was detected by using a molecular probe, 9,10‐anthracenediyl‐bis(methylene)dimalonic acid (ABDA), which selectively reacts with ^1^O_2_ in solution. The sample solution was prepared by mixing BBP/AuNPs/TCPP with ABDA (50 µg mL^−1^). The solutions were irradiated at 650 nm with a power density of 0.5 W cm^−2^. The absorbance of the solution at 400 nm was recorded at intervals of 1 min using a UV‐vis spectrometer.

We employed a 2′,7′‐dichlorodihydrofluorescein diacetate (DCFH‐DA) assay to detect the diffusion distance of ^1^O_2_ under irradiation. DCFH was generated by reacting DCFH‐DA with 0.1 M NaOH at ambient temperature for 30 min [41]. Briefly, 100 µL as‐prepared *S. aureus* suspension (∼1 × 10^7^ CFU mL^−1^) was inoculated on 9 mm round coverslip in a 48‐well plate at 37°C for 48 h to form *S. aureus* biofilms. Then, the culture medium was removed, and the biofilm was divided into different groups. The mature biofilm was incubated with PBS, BBP/AuNPs, AuNPs/TCPP, and BBP/AuNPs/TCPP (0.5 mg mL^−1^), separately, for 1 h, and the excessive solution was washed by PBS. Then, the biofilm was exposed to 808 nm irradiation at an intensity of 1.0 W cm^−2^ for 10 min, followed by 650 nm irradiation at 0.5 W cm^−2^ for an additional 10 min. DCFH (10 µM) was added and incubated at 37°C for 30 min. Then the ^1^O_2_ fluorescent images could be photographed by confocal laser scanning microscope (CLSM).

### 4.9 Antibiofilm Activity Evaluation In Vitro

The mature *S. aureus* biofilm was incubated with PBS, BBP/AuNPs, AuNPs/TCPP, and BBP/AuNPs/TCPP (0.5 mg mL^−1^) for 1 h, and the excessive solution was washed by PBS. After incubation, the biofilm was first subjected to an 808 nm irradiation (1.0 W cm^−2^, 10 min), followed by exposure to a 650 nm irradiation (0.5 W cm^−2^, 10 min). The biofilm suspension was collected by vortex and ultrasonic, and the live bacteria were counted by plate counting method. The bacterial biofilms that underwent the above treatments were then stained with Hoechst 33342 and PI mixture for 30 min in dark, and the fluorescence images were recorded by CLSM. We also observed the morphology of the bacteria in the biofilm using SEM. Briefly, the biofilm was fixed with 2.5% glutaraldehyde and then dehydrated with ethanol of different gradients. dried and coated with platinum sputtering, then the sample was observed.

### 4.10 Biofilm Quantitation

The  biofilm biomass was stained and quantified by crystal violet staining according to previous literature [42]. The biofilm was stained with 0.1% crystal violet solution for 15 min. After removing excess dye, the biofilms were allowed to dry upside down overnight at room temperature. After incubation with 30% acetic acid for 15 min, the absorbance at 590 nm was recorded and the biomass was quantified.

### 4.11 Animal Experiment Ethics

All animal experiments in this study were approved by the Animal Ethical and Welfare Committee of the General Hospital of Northern Theater Command (2025‐01). All procedures and surgical operations were conducted according to current guidelines.

### 4.12 Mouse Biofilm‐Associated Infection Model

The infection model was constructed using BALB/c mice (male, 20–22 g). Briefly, the circular wound with a diameter of 1.0 cm was formed on the back of the mice. Then, 100 µL *S. aureus* or MRSA suspension (10^7^ CFU mL^−1^) was injected on the surface of the wound. After a 3‐day infection, the successfully infected mice were divided into seven groups with 10 mice in each group and treated with PBS, light irradiation, WT‐M13/AuNPs, BBP/AuNPs, AuNPs/TCPP, WT‐M13/AuNPs/TCPP, and BBP/AuNPs/TCPP, respectively. The body weight and wound area of mice were monitored. The wound was collected, and the infected skin was ground into a 10% tissue homogenate. The tissue homogenates were continuously diluted in PBS and counted by plate count. On the 14th day, the skin and organs were collected for H&E staining and Masson staining.

### 4.13 Biosafety Study

MTT assay was used to determine the cytotoxicity of BBP/AuNPs, AuNPs/TCPP, and BBP/AuNPs/TCPP. Briefly, RAW264.7 cells were incubated with various concentrations of BBP/AuNPs, AuNPs/TCPP, and BBP/AuNPs/TCPP in a 96‐well plate for 24 h. Then, 50 µL of 1 × MTT solution was added to each well. After 4 h‐incubation, DMSO was added and incubated for 10 min, and the absorbance at 490 nm of the solution was recorded with a microplate reader. Cell viability was calculated using the following equation:

(5) $$\mathrm{Cell}\;\mathrm{viab}\mathrm{ility}\;\;\left(\%\right)=\frac{A_{\mathrm{trea}\mathrm{ted}}-A_{\mathrm{blank}}}{A_{\mathrm{cont}\mathrm{rol}}-A_{\mathrm{blank}}}\;\times 100\%$$

where *A*_treated_ is the absorbance obtained in the presence of AuNPs/TCPP, BBP/AuNPs, or BBP/AuNPs/TCPP; *A*_control_ is the absorbance of the cell‐only control group cultured in DMEM medium; *A*_blank_ is the absorbance of cell‐free blank wells.

The cytotoxicity of AuNPs/TCPP, BBP/AuNPs, and BBP/AuNPs/TCPP under light irradiation (808 nm for PTT + 650 nm for PDT) was estimated. Finally, the MTT assay was used to estimate the cytotoxicity.

To investigate the hemocompatibility of AuNPs/TCPP, BBP/AuNPs, and BBP/AuNPs/TCPP, the hemolysis assay was performed using mouse blood samples. Briefly, 1 mL of fresh blood was collected from healthy mice. Pure red blood cells (RBCs) were obtained by centrifugation at 3000 rpm for 15 min and washed three times. The pure RBCs were diluted with PBS to obtain an RBC suspension with a final concentration of 5%. Then, 100 µL RBCs suspension was mixed with AuNPs/TCPP, BBP/AuNPs, and BBP/AuNPs/TCPP solution (0.5 mg mL^−1^) and incubated for 1.5 h at 37°C. Similarly, PBS and 2% Triton X‐100 were mixed with the RBC suspension as the negative and positive controls, respectively. After incubation, all samples were centrifuged at 1000 rpm, and the absorbance of the released hemoglobin in the supernatant was measured at 540 nm. The hemolysis ratio was calculated using the following equation:

(6) $$Hemolysis\;ratio\;\left(\%\right)=\frac{A-A_{n}}{A_{p-}A_{n}}\;\times 100\%$$

where *A*, *A*_n_, and *A*_p_ represent the absorbance of the samples, the negative control group, and the positive control group, respectively.

To assess the biosafety of light irradiated BBP/AuNPs/TCPP, major organs of mice (heart, lungs, liver, spleen, and kidneys) were collected and excised for pathological analysis. All tissue samples were fixed in 4% paraformaldehyde, dehydrated, embedded in paraffin for slices fabrication. Finally, H&E staining was performed for the biosafety evaluation.

### 4.14 Statistical Analysis

All experiments were conducted with biological replicates and repeated at least three times. Data were expressed as means ± SD (*n* ≥ 3). Statistical analyses were performed using GraphPad Prism 10 software (GraphPad Software, San Diego, CA, USA). For comparisons between two groups, an unpaired two‐tailed Student's t‐test was used. For comparisons among multiple groups, one‐way analysis of variance (ANOVA) was performed, followed by Tukey's post hoc test for pairwise comparisons. *p < 0.05, **p < 0.01, ***p < 0.001, ****p < 0.0001, n.s means no significance.

## Conflicts of Interest

The authors declare no conflicts of interest.

## Supporting information




**Supporting File 1**: advs76405‐sup‐0001‐SuppMat.docx.

**Supporting File 2**: advs76405‐sup‐0002‐DataFile.pdf.

## Data Availability

The data that support the findings of this study are available from the corresponding author upon reasonable request.
