# Supplementary material for: Biofilm‐Binding Phages Enhance Biofilm Eradication by Synergistic Photothermal and Photodynamic Therapy
Source: Adv Sci (Weinh). 2026 Jul 8:e23904. Online ahead of print. doi: 10.1002/advs.202523904 (PMC13345313; doi:10.1002/advs.202523904)
Supplement: Supplementary file 1 — Supporting File 1: advs76405‐sup‐0001‐SuppMat.docx. [file ADVS-9999-e23904-s001.docx]

**Biofilm-binding Phages Enhance Biofilm Eradication by Synergistic Photothermal and Photodynamic Therapy**

*Ying Cao ^a#^, Tao Yang ^b#^, Rui Wang ^a^，Hui-Da Li ^a^, Xiao-Yu Zhang ^c^, Feng Cheng ^d^,* *Ying Liu ^d*^, Jian-Hua Wang ^a^, Ting Yang ^a*^ and Chuanbin Mao ^b*^*

a, Research Center for Analytical Sciences, Department of Chemistry, College of Sciences, Northeastern University, Shenyang 110819, China

b, Department of Biomedical Engineering, The Chinese University of Hong Kong, Hong Kong SAR, China.

c, CAS Key Laboratory of Separation Science for Analytical Chemistry, Dalian Institute of Chemical Physics Chinese Academy of Sciences, Dalian 116023, China

d, Department of Emergency, General Hospital of Northern Theater Command, Shenyang 110016, China

^#^Ying Cao and Tao Yang contributed equally to this work.

*Correspondence should be addressed to Chuanbin Mao ([cmao@cuhk.edu.hk](mailto:cmao@cuhk.edu.hk)), Ting Yang ([yangting@mail.neu.edu.cn](mailto:yangting@mail.neu.edu.cn)) or Ying Liu ([cszx_ly@163.com](mailto:cszx_ly@163.com))


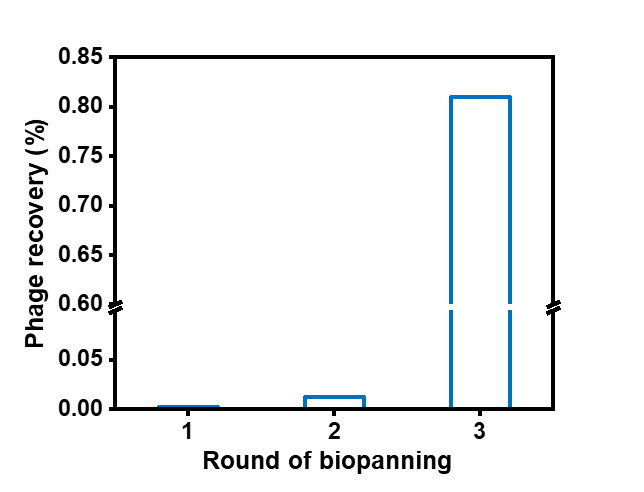


## Figure S1. Phage recovery rate after each round of biopanning.


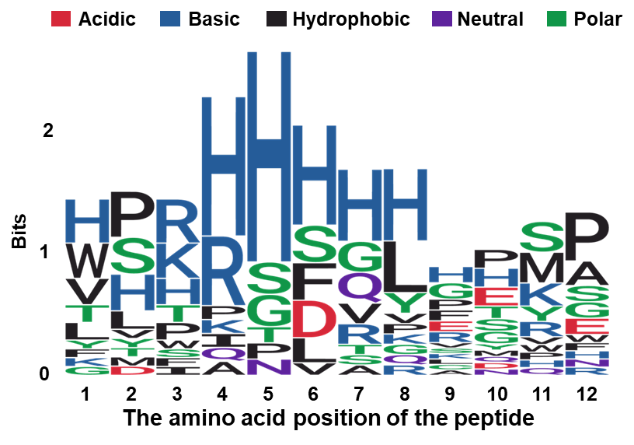


## Figure S2. Peptide sequence analysis of the selected biofilm-binding phages.


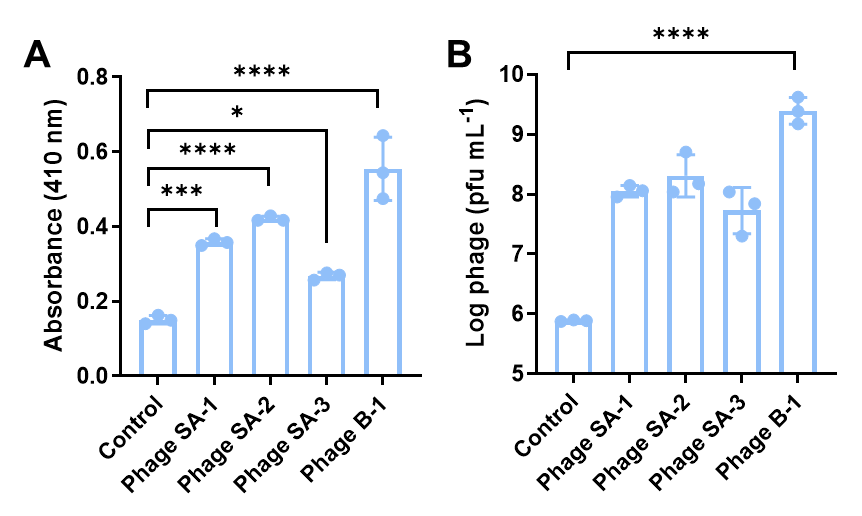


## Figure S3. (A) The binding affinity of the selected phages to biofilms via phage ELISA. (B) The amount of the phages binding on the biofilm. Phage SA-1, SA-2, and SA-3 refer to those obtained by the biopanning against free *S. aureus*, whereas Phage B-1 refers to the phages screened by the biopanning against *S. aureus* biofilms.


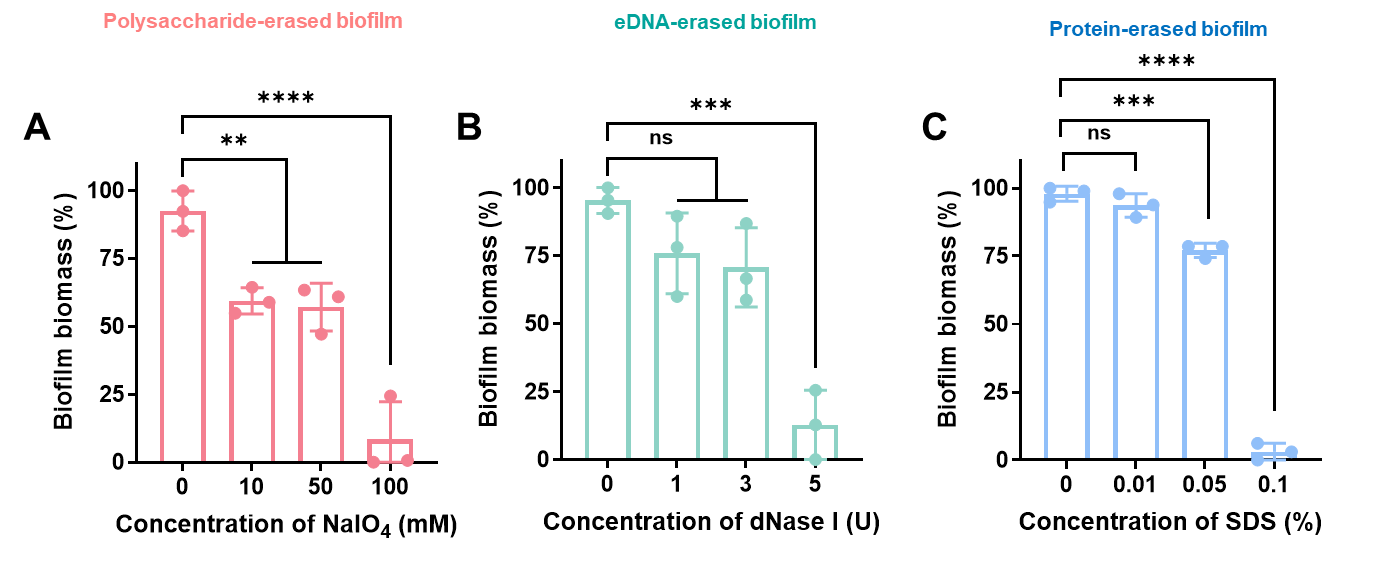


## Figure S4. Biomass of *S. aureus* biofilms after various treatments. (A)The biofilms were treated with 10~100 mM of NaIO_4_ to remove polysaccharides; (B) The biofilms were treated with 1~5 U of dNase I to remove eDNA; (C) The biofilms were treated with 0.01%~0.1% of SDS to remove protein.


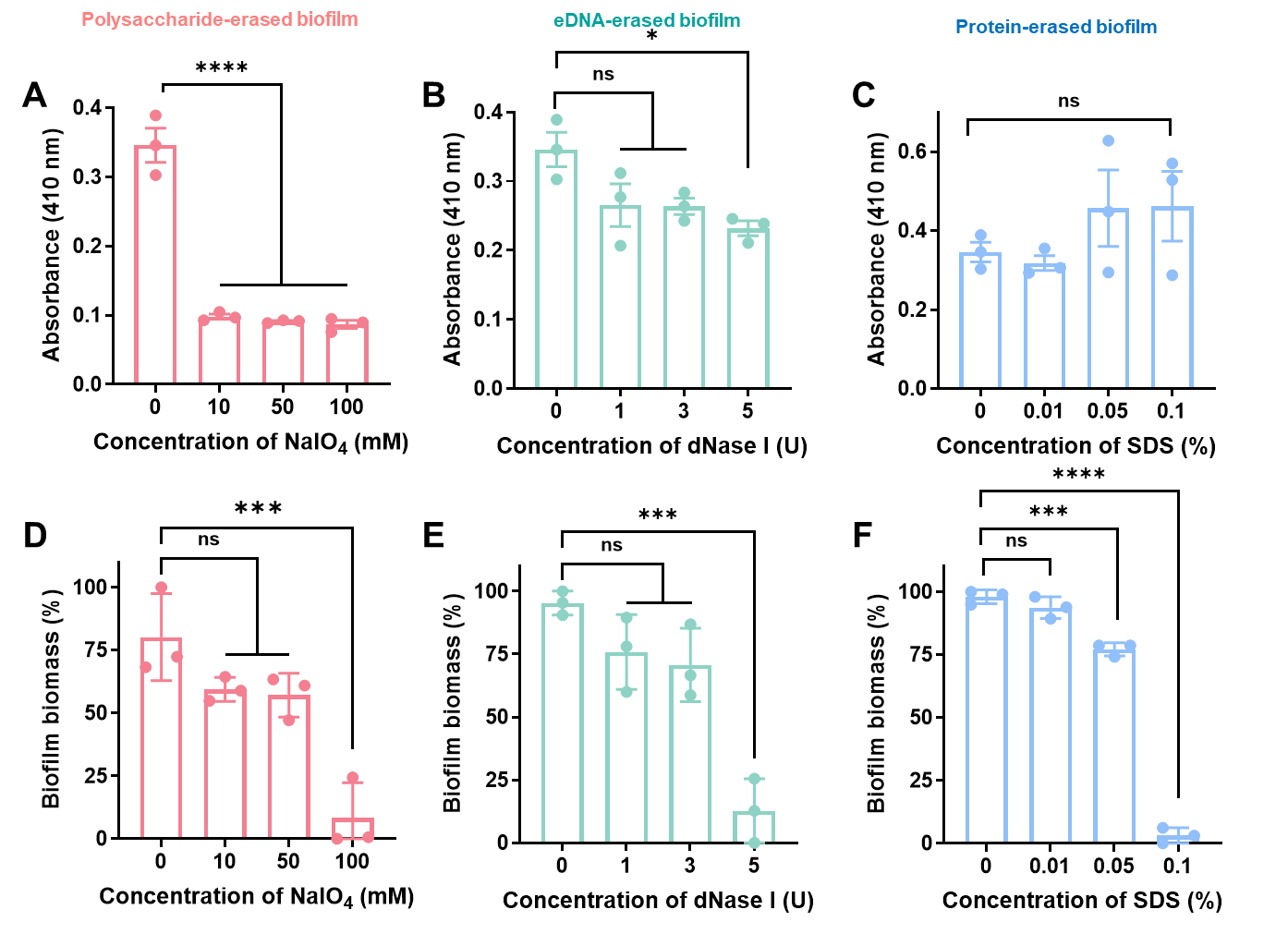


## Figure S5. Binding affinity of BBP to *S. aureus* biofilms via phage ELISA. (A)The biofilms were treated with 10~100 mM of NaIO_4_ to remove polysaccharides; (B) The biofilms were treated with 1~5 U of dNase I to remove eDNA; (C) The biofilms were treated with 0.01%~0.1 % of SDS to remove protein.


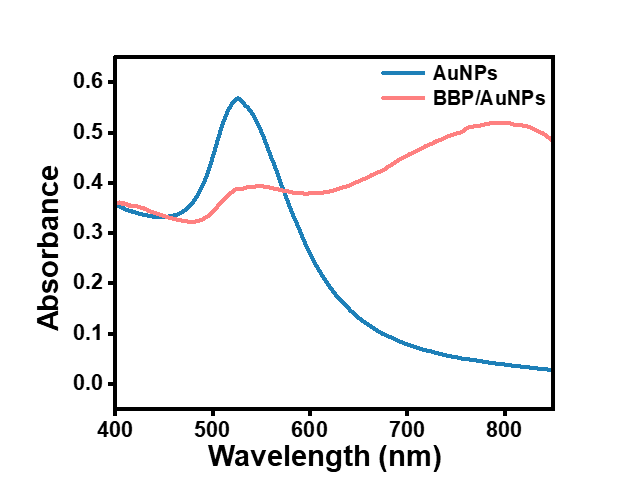


## Figure S6. The UV-vis spectra of AuNPs and BBP/AuNPs.


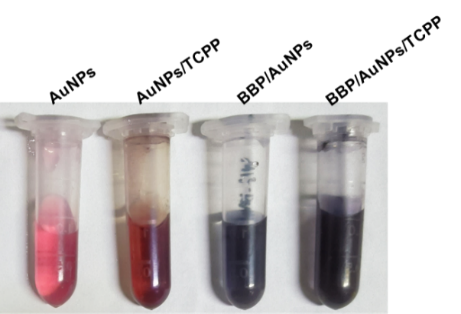


## Figure S7. The photographs of AuNPs, AuNPs/TCPP, BBP/AuNPs and BBP/AuNPs/TCPP.


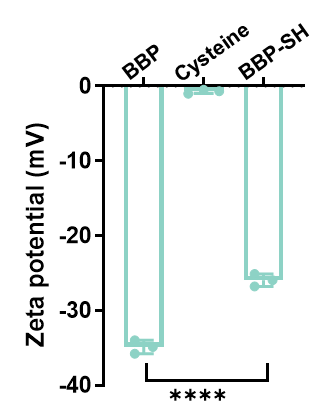


## Figure S8. The zeta potential of BBP, cysteine and BBP-SH.


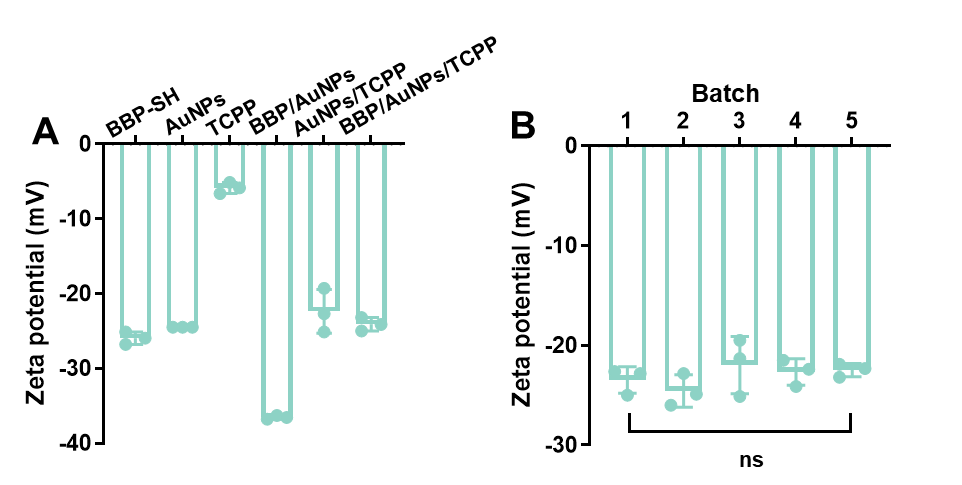


## Figure S9. (A)The zeta potential of BBP-SH, AuNPs, TCPP, AuNPs/TCPP, BBP/AuNPs and BBP/AuNPs/TCPP; (B) The zeta potential of BBP/AuNPs/TCPP prepared from different batches.


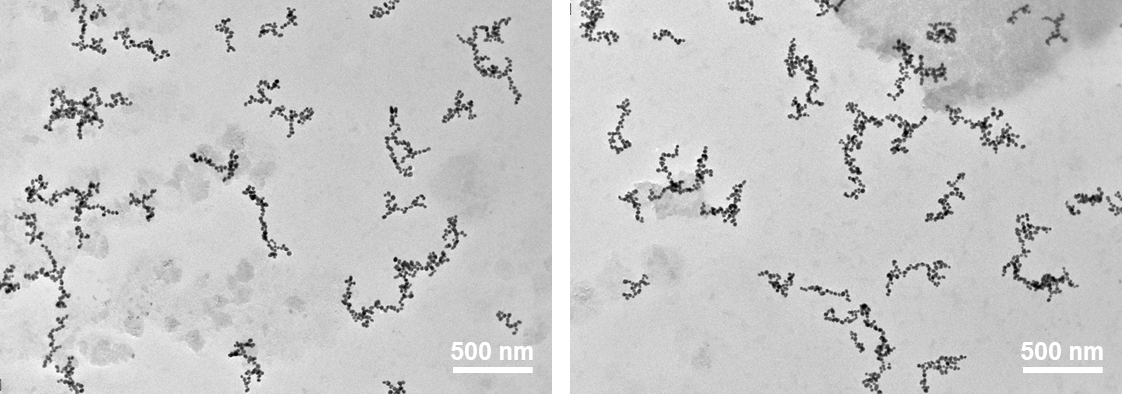


## Figure S10. The larger field TEM images of BBP/AuNPs/TCPP.


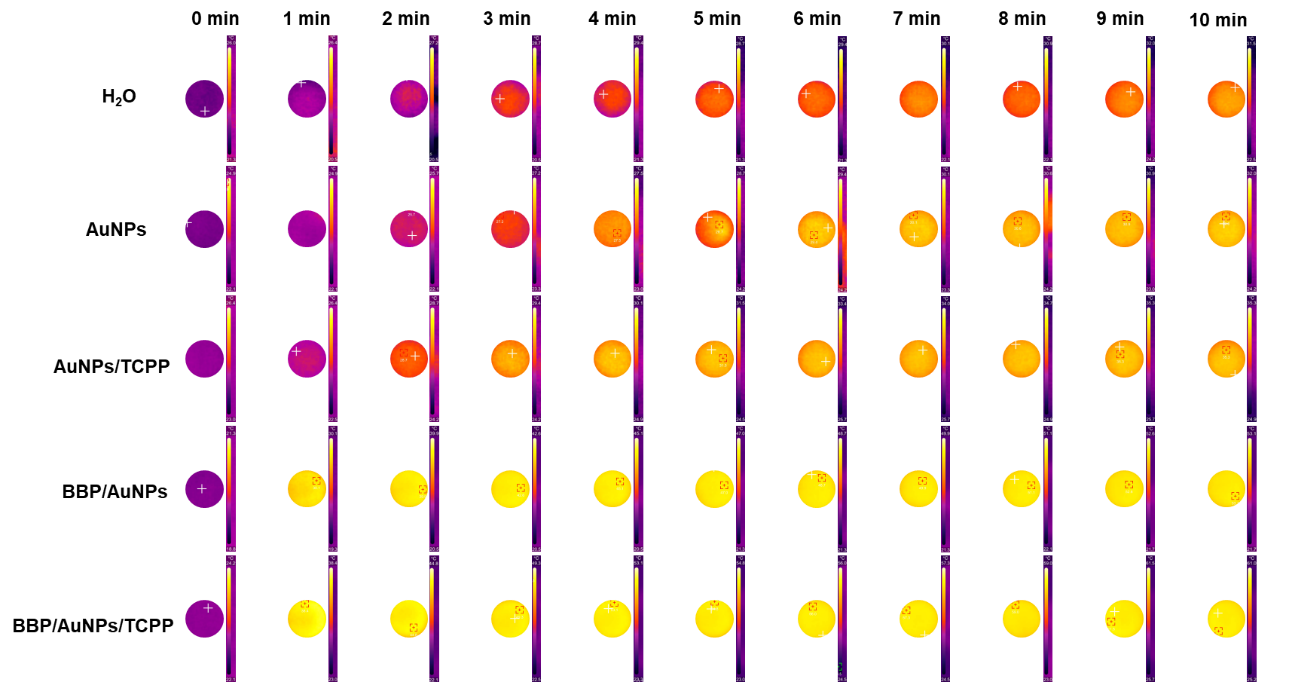


## Figure S11. Photographs reflecting the temperature changes of AuNPs, AuNPs/TCPP, BBP/AuNPs, and BBP/AuNPs/TCPP under 808 nm irradiation with an intensity of 1.0 W cm^-2^.


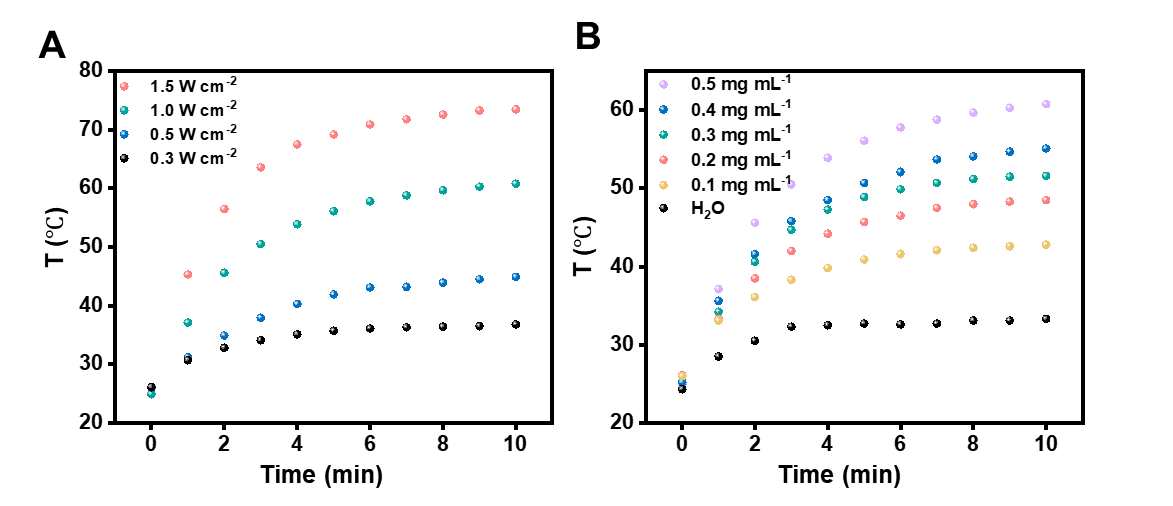


## Figure S12. Temperature change of the BBP/AuNPs/TCPP (A) upon 808 nm irradiation with varying irradiation intensities, and (B) at different concentrations upon 808 nm irradiation (1.0 W cm^-2^).


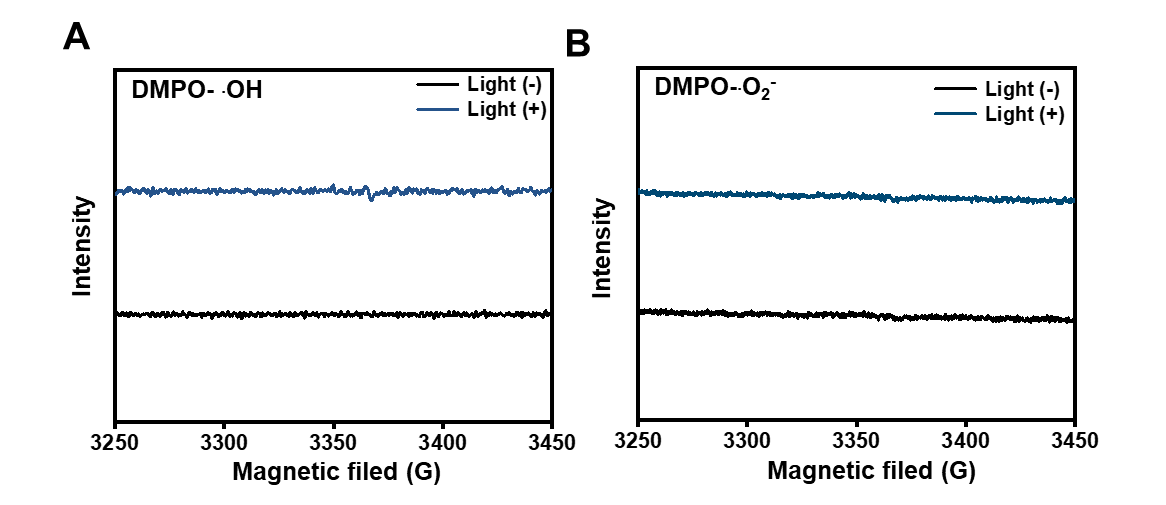


## Figure S13. EPR spectra of BBP/AuNPs/TCPP under 650 nm laser irradiation of DMPO/•OH spin adduct (A) and DMPO/•O^2−^ spin adduct (B).


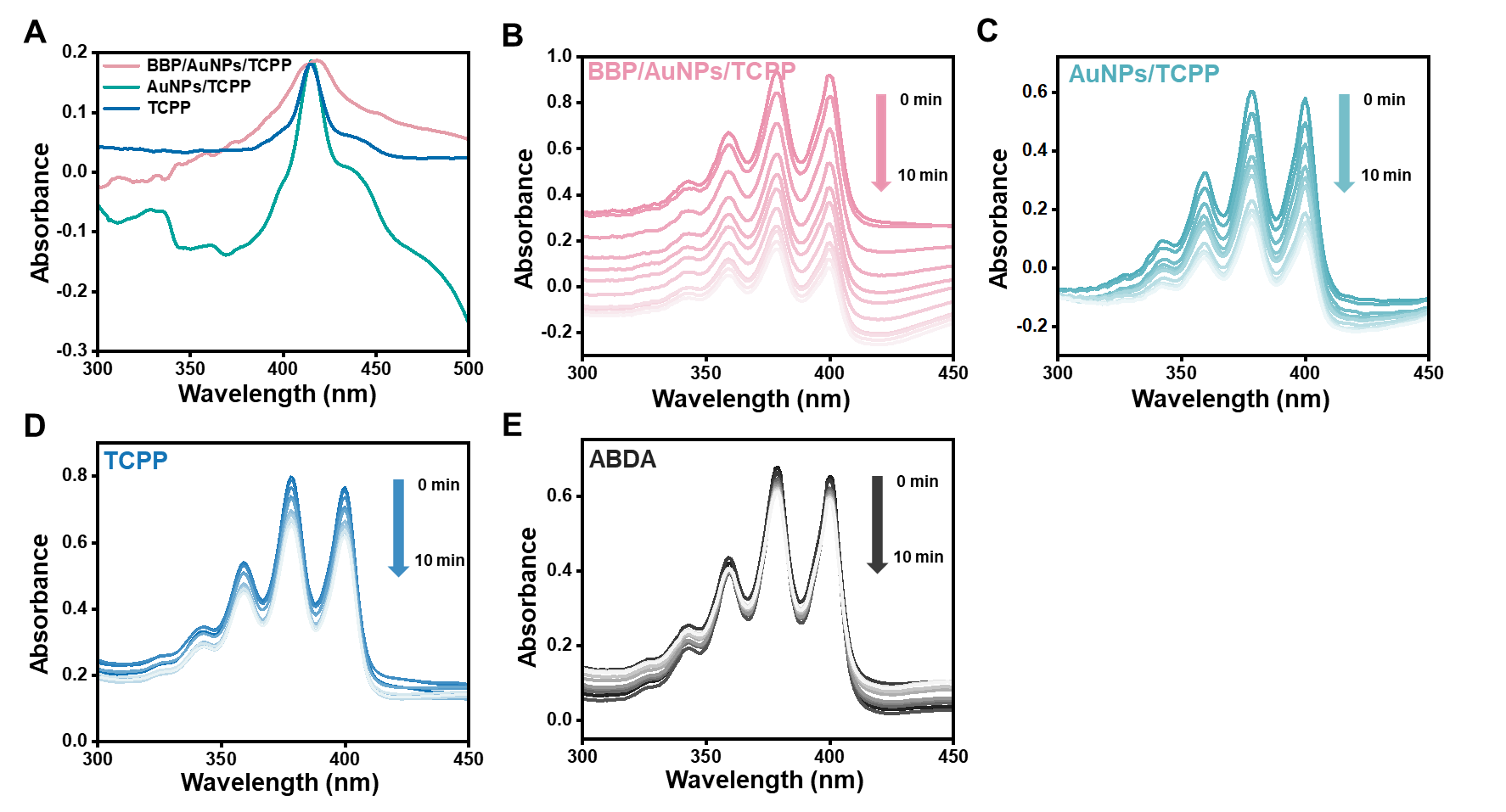


## Figure S14. (A) The UV-vis absorption spectra of TCPP, AuNPs/TCPP and BBP/AuNPs/TCPP. The UV-vis absorption spectra of ABDA in the presence of (B) BBP/AuNPs/TCPP, (C) AuNPs/TCPP, (D) TCPP, and (E) H_2_O under 650 nm irradiation with an intensity of 0.5 W cm^-2^.

## Figure S15. The diffusion distance of ^1^O_2_ within the biofilm by DCFH-DA probe after various treatments.


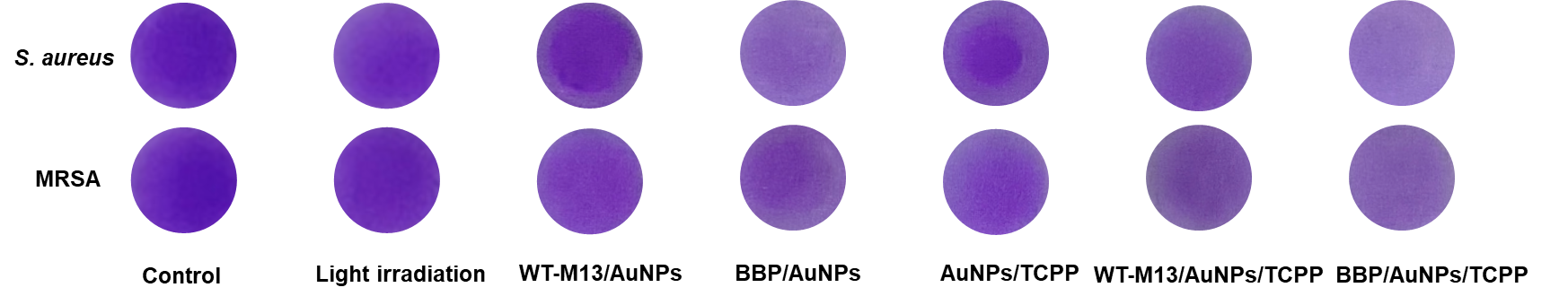


## Figure S16. Crystal violet staining of the biofilms after different treatments.


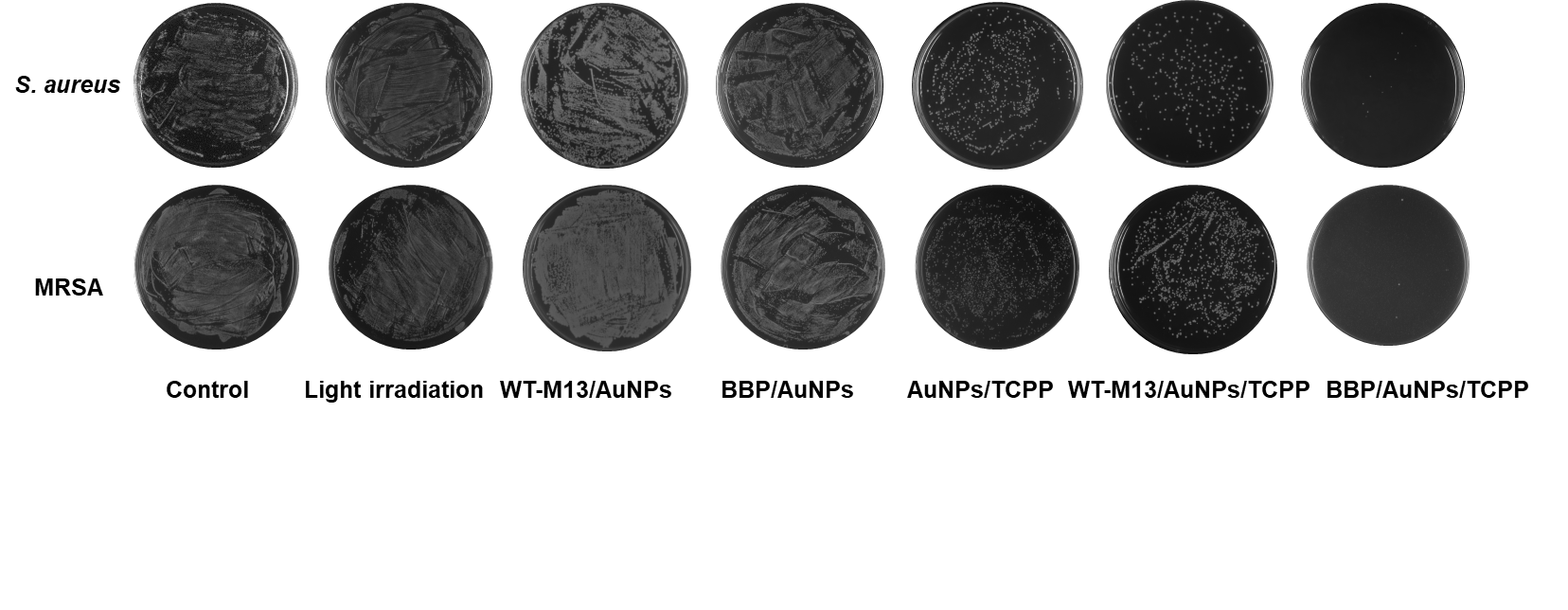


## Figure S17. Photographs of colonies within biofilms after various treatments.


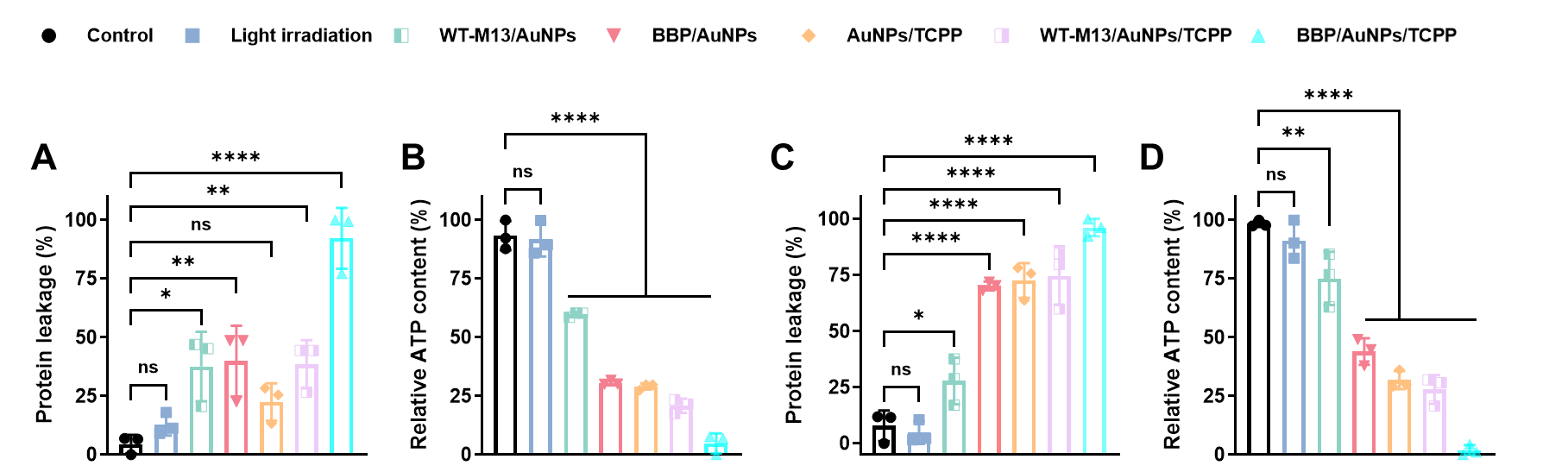


## Figure S18. The leakage of bacterial proteins (A) and the level of ATP (B) after different treatments in vitro. The leakage of bacterial proteins (C) and the level of ATP (D) after different treatments in vivo.


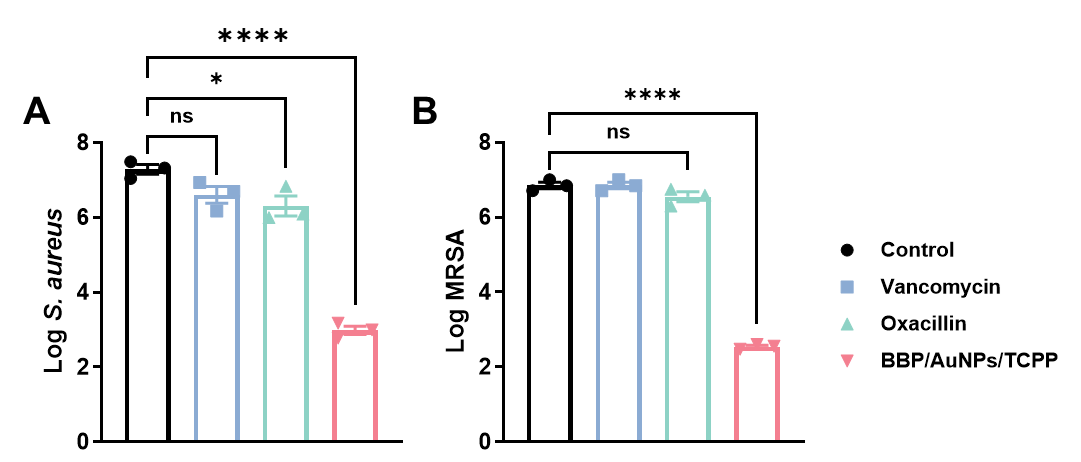


## Figure S19. (A) Numbers of viable *S. aureus* within the *S. aureus* biofilms after various treatments. (B) Numbers of viable MRSA within the MRSA biofilms after various treatments.

**
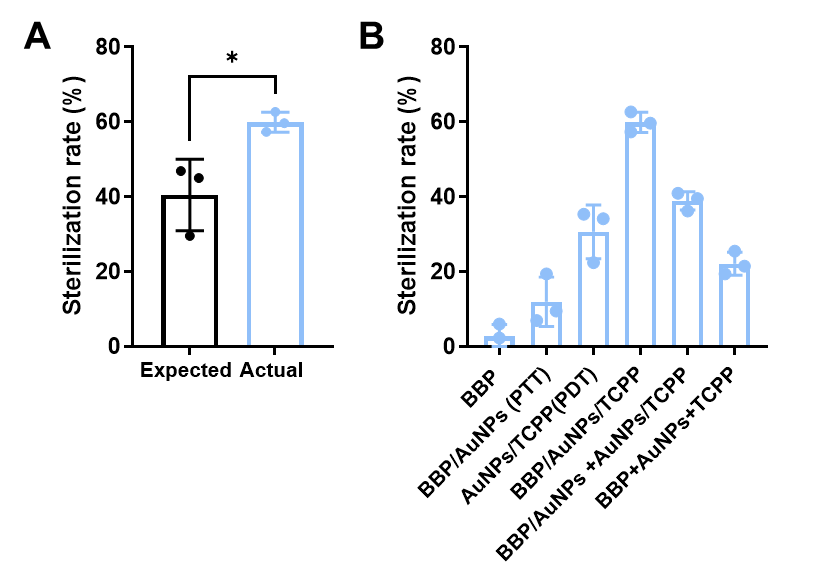
**

## Figure S20. (A) Bliss independence analysis of the synergistic bactericidal effect of the BBP/AuNPs/TCPP combination. (B) The sterilization rate of *S. aureus* biofilms after various treatments.


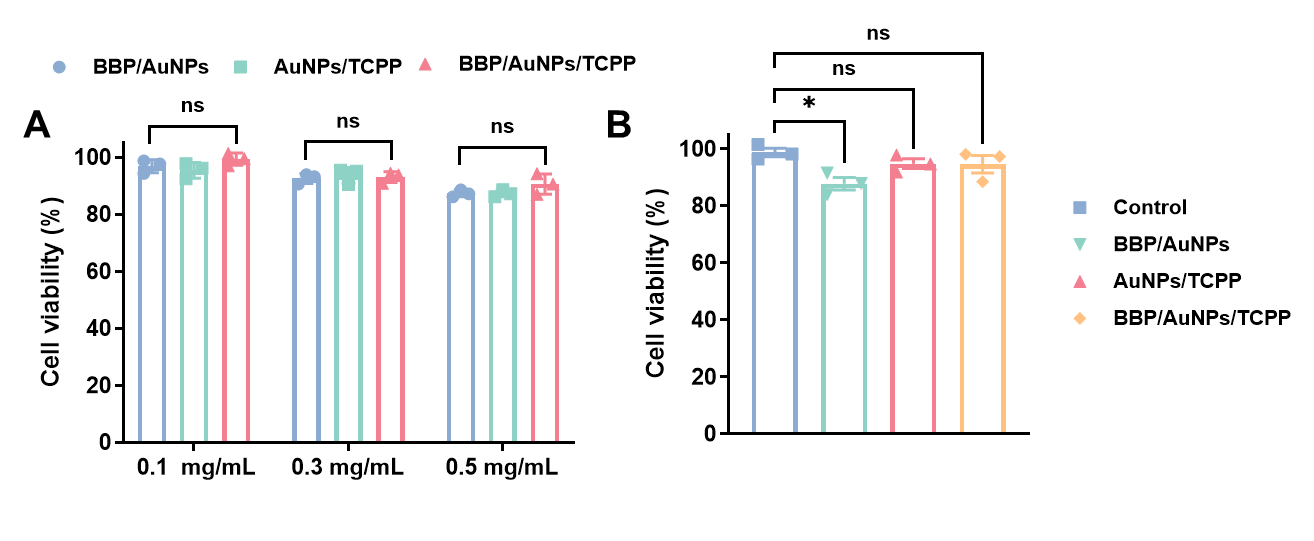


## Figure S21. (A) Viability (%) of RAW264.7 cells after incubated with different concentrations of BBP/AuNPs, AuNPs/TCPP and BBP/AuNPs/TCPP. (B) Viability (%) of RAW264.7 cells after incubated with 0.5 mg mL^-1^ BBP/AuNPs, AuNPs/TCPP and BBP/AuNPs/TCPP under light irradiation (808 nm + 650 nm).


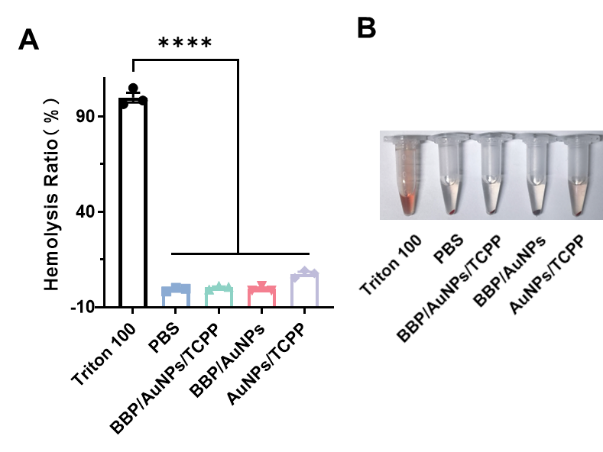


## Figure S22. (A) The hemolysis ratio of blood cells with different treatments and (B) the corresponding hemolysis photographs.


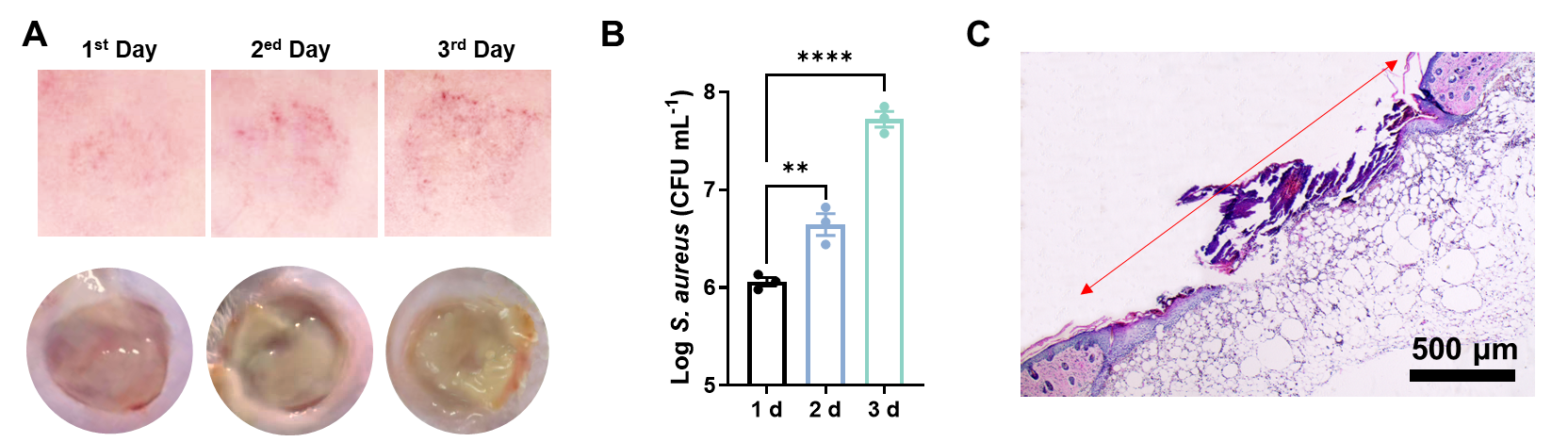


## Figure S23. (A) Biofilm detection by using wound blotting method. (B) Statistical studies of bacterial colonies obtained from the wound tissues. (C) H&E staining of the wound tissue.


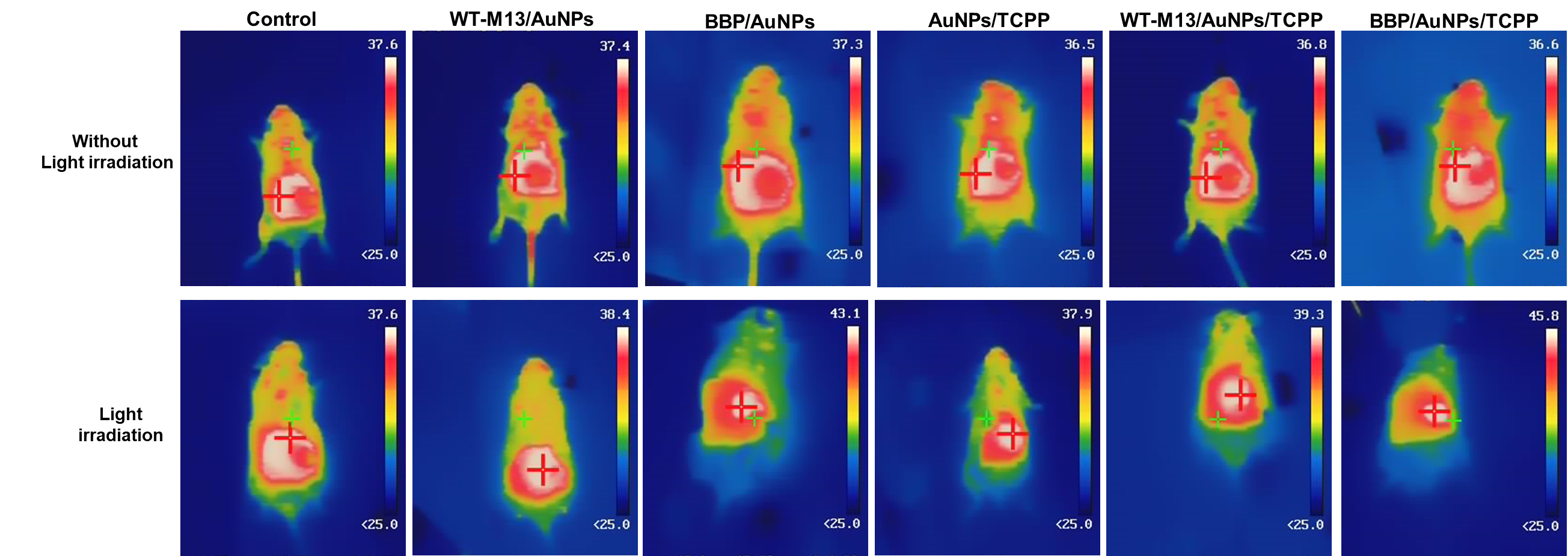


## Figure S24. Thermal images of control, BBP/AuNPs, AuNPs/TCPP and BBP/AuNPs/TCPP treatment of infected wounds before and after 808 nm irradiation.

**
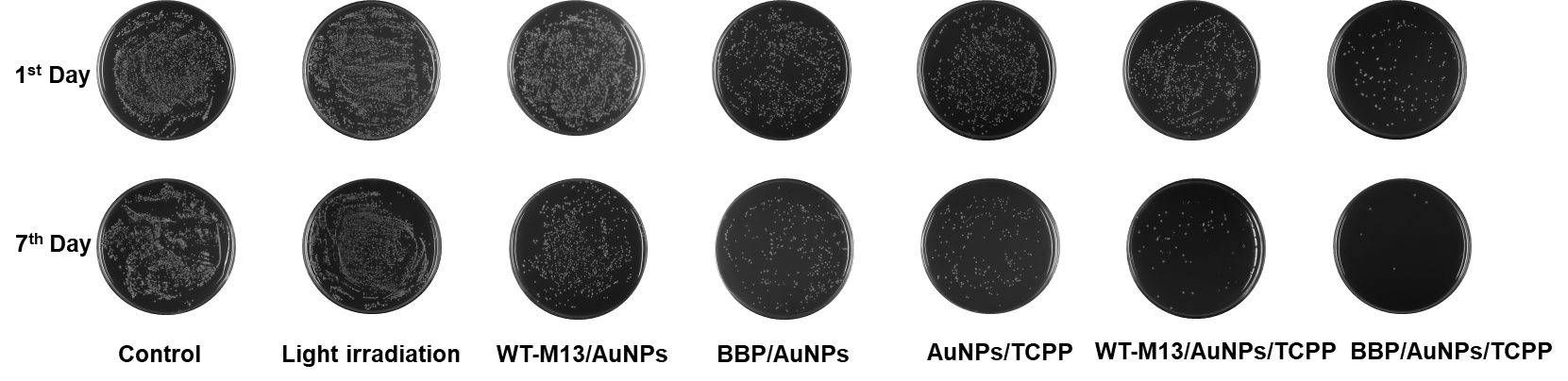
**

## Figure S25. Photographs of colonies within wounds biofilms after various treatments.


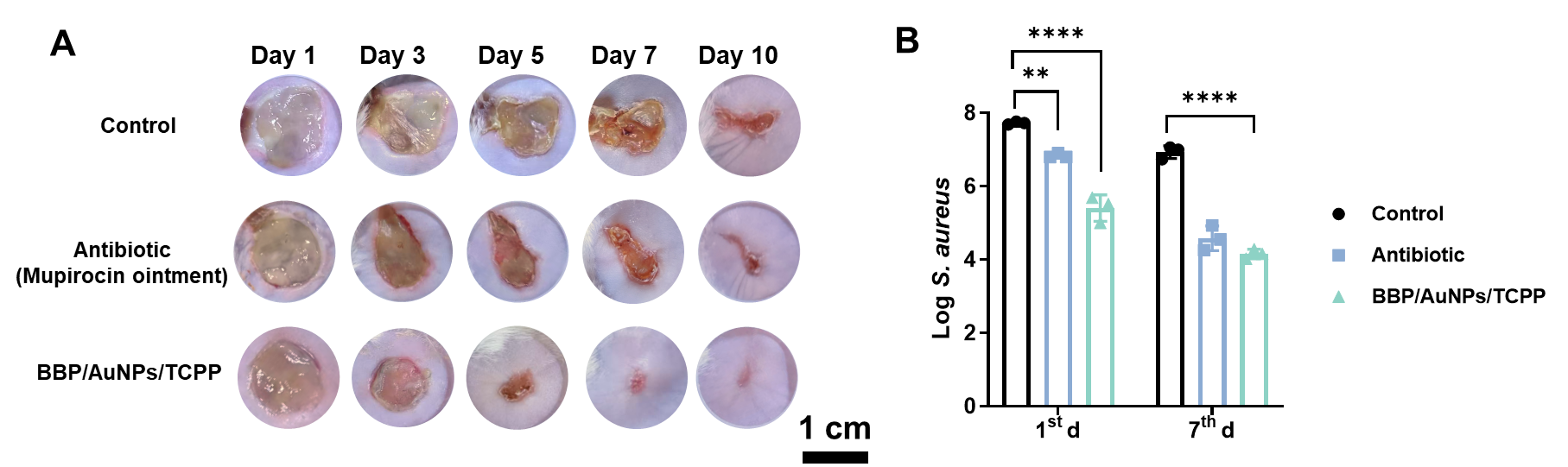


## Figure S26. (A) Representative photographs of the wounds treated with BBP/AuNPs/TCPP and antibiotic drugs (The images of the Control and BBP/AuNPs/TCPP groups are derived from Figure 5B). (B) Statistical studies of bacterial colonies obtained from the wound tissues after BBP/AuNPs/TCPP and antibiotic treatments.


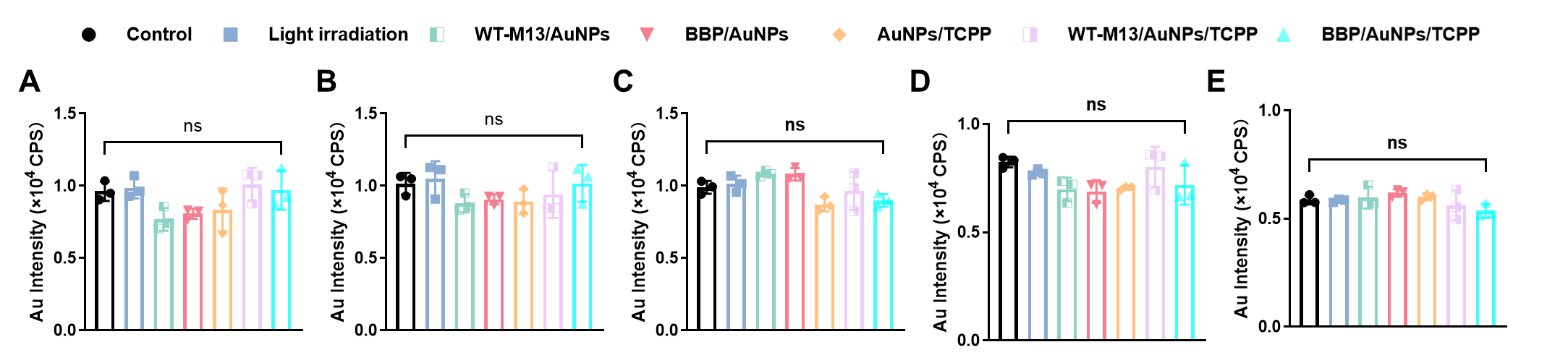


## Figure S27. ICP-MS counts of Au in mouse organs (A-E: heart, liver, spleen, lung and kidney) after treatment.

**
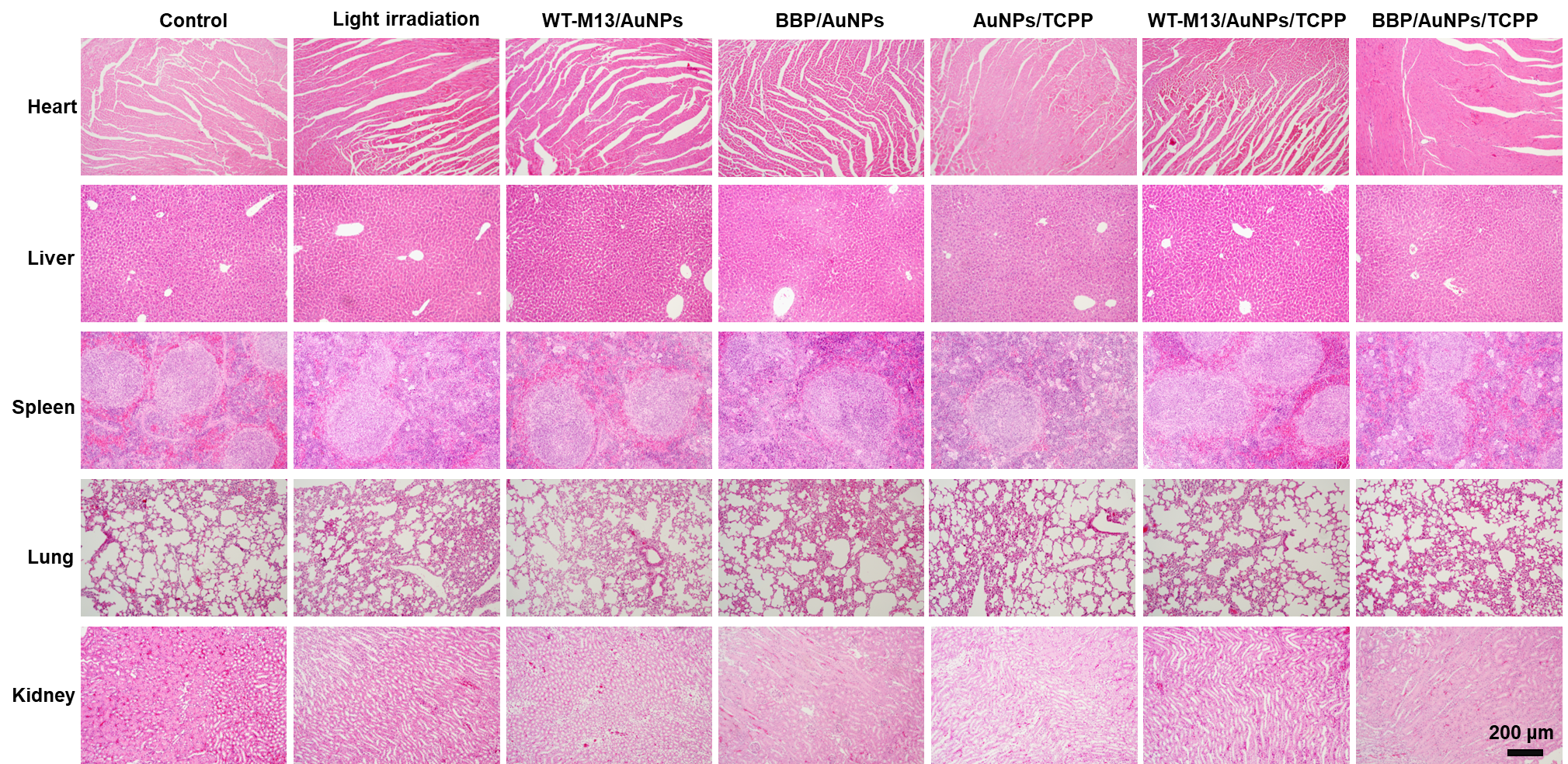
**

## Figure S28. Representative H&E staining images of major organs in mice infected with *S. aureus* biofilm, collected on Day-14 after different treatments.

**
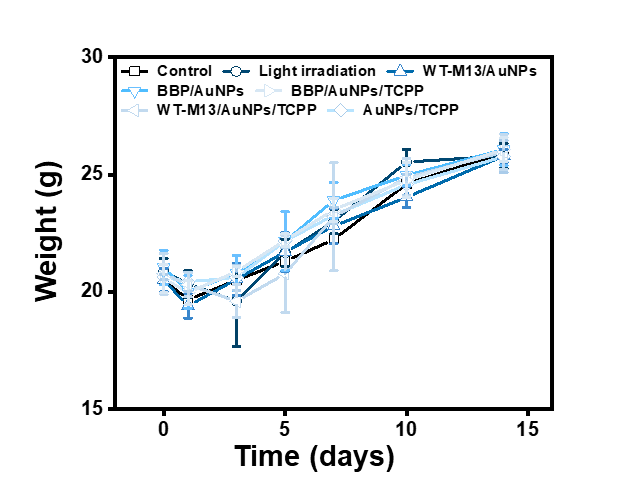
**

## Figure S29. The weight of mice infected with *S. aureus* biofilm in different treatment groups.


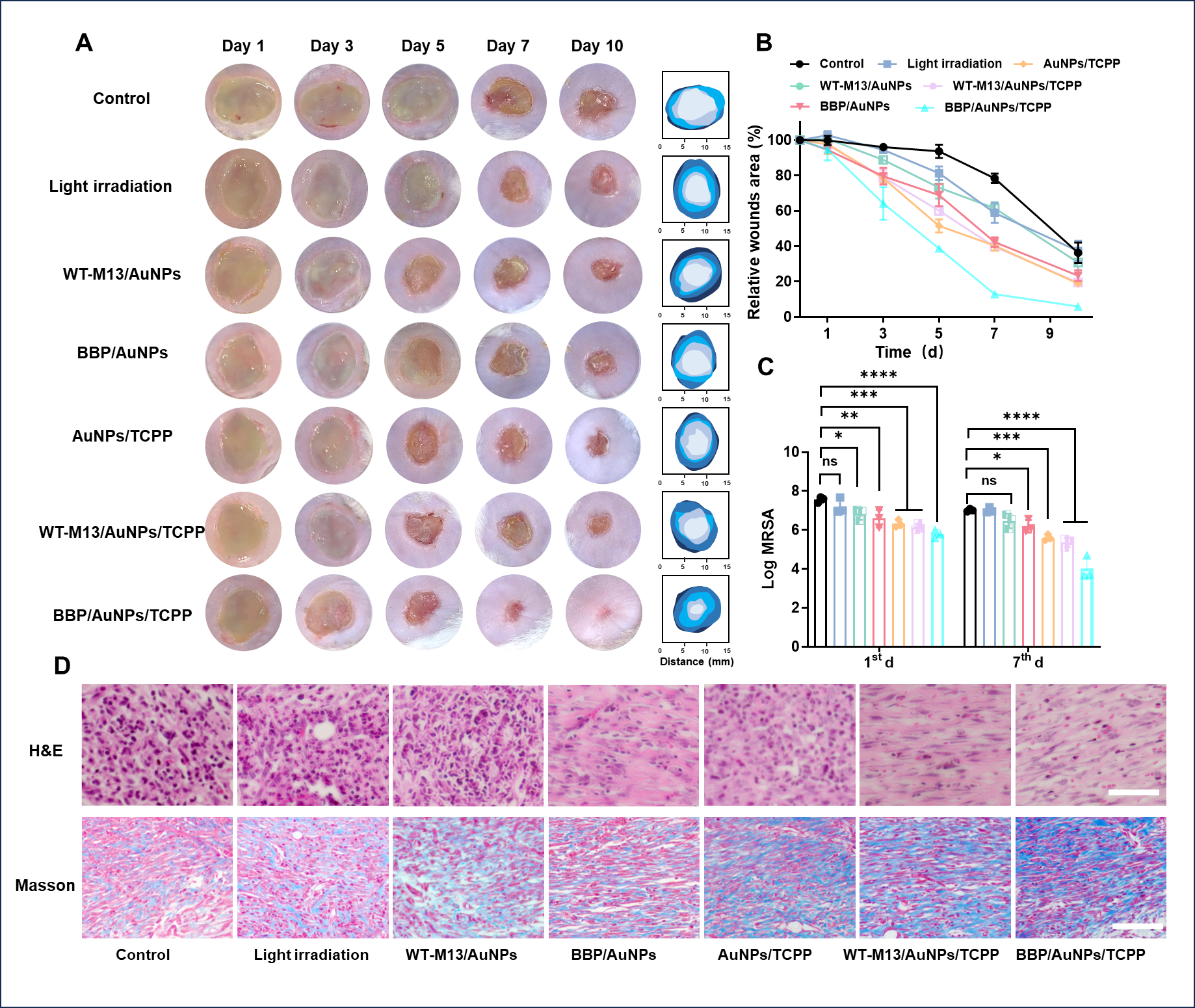


**Figure S30.** The therapeutic effects of BBP/AuNPs/TCPP to achieve the in vivo treatment of the MRSA biofilms. (A) Photographs of wound healing condition during 10-day treatment by different ways, along with the overlaid images. (B) Corresponding quantitative evaluation of relative wounds area over time. (C) Statistical analysis of bacterial colonies obtained from the wound tissues after various treatments. (D) H&E and Masson staining of the peripheral tissue after various treatments at the 14th day. Scale bar is 50 μm.

**Figure S31.** Statistical analysis of bacterial colonies obtained from the wound tissues at day 10 post-treatment.


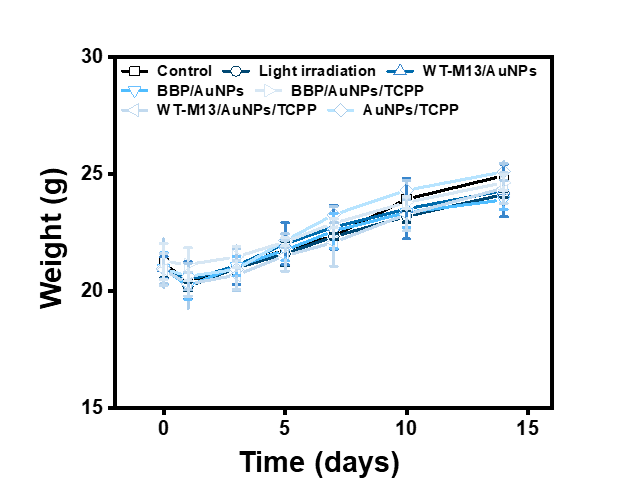


**Figure S32.** The weight of mice infected with MRSA biofilms in different treatment groups.


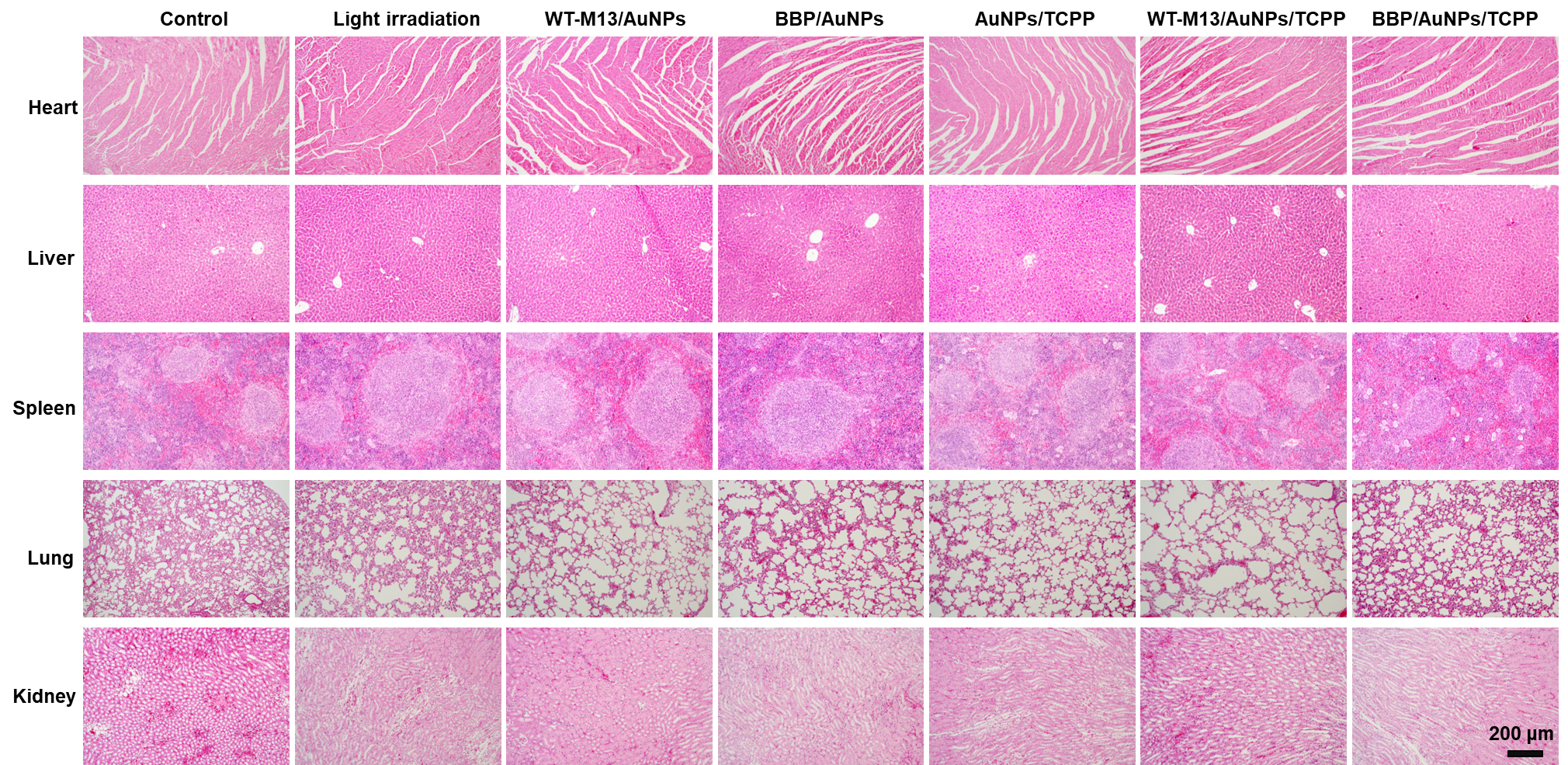


## Figure S33. Representative H&E staining images of major organs in mice infected with MRSA biofilms, collected on Day-14 after different treatments.


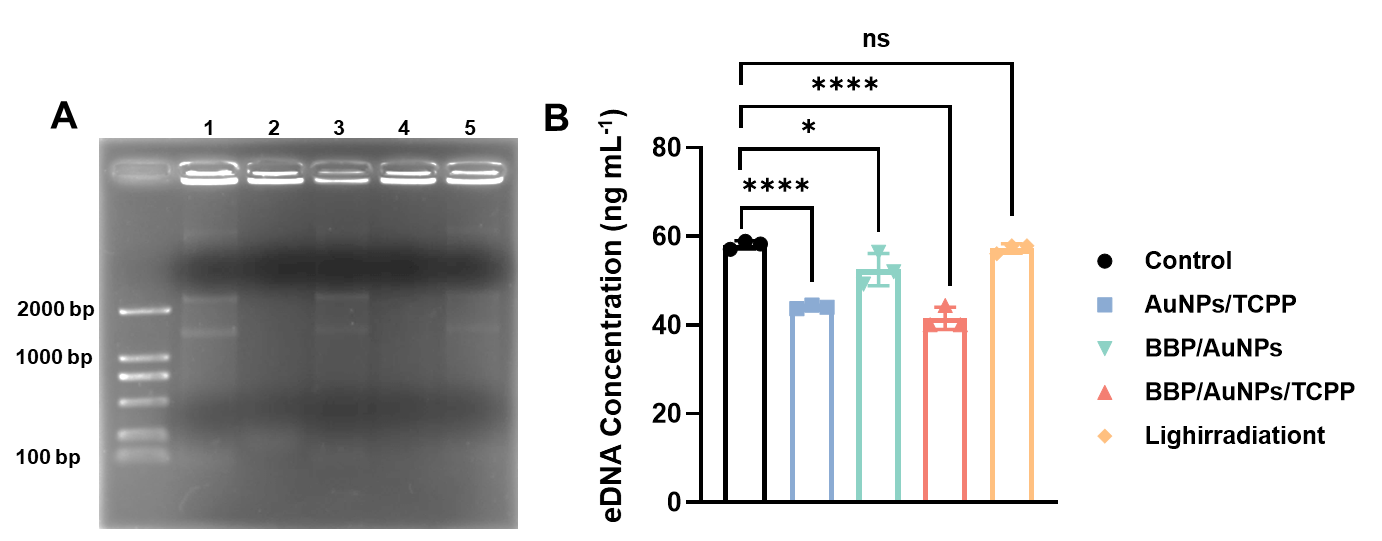


## Figure S34. (A) The AGE characterization of the eDNA in biofilm with various treatment. Lane 1: Control, Lane 2: AuNPs /TCPP, Lane 3: BBP/AuNPs, Lane 4: BBP/AuNPs/TCPP, Lane 5: Light irradiation (808 nm+650 nm). (B) The relative amount of eDNA in biofilms after various treatments.


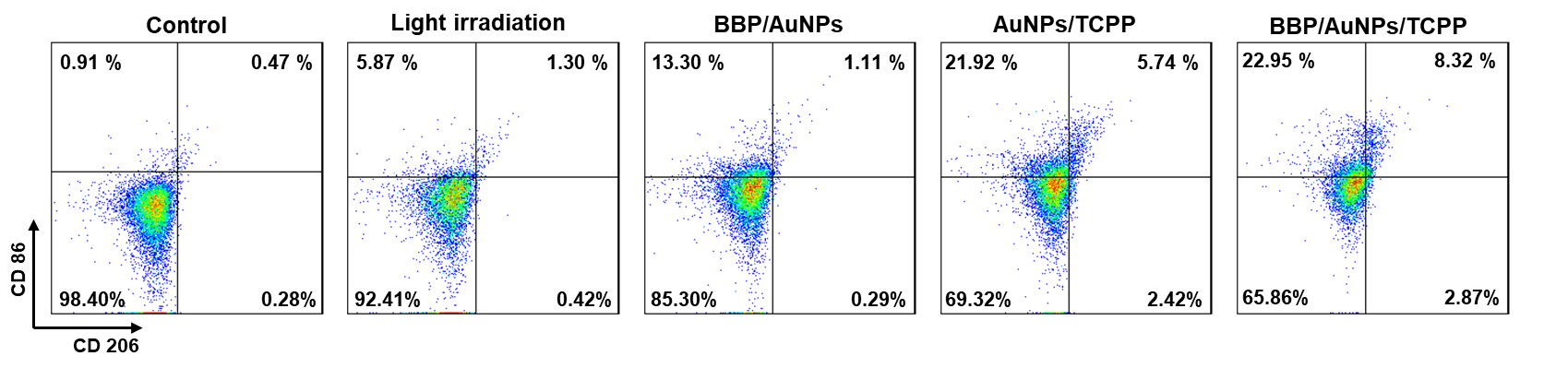


## Figure S35. Representative flow-cytometry dot plots of macrophage polarization markers CD86 (M1) and CD206 (M2) under the indicated treatments.

**
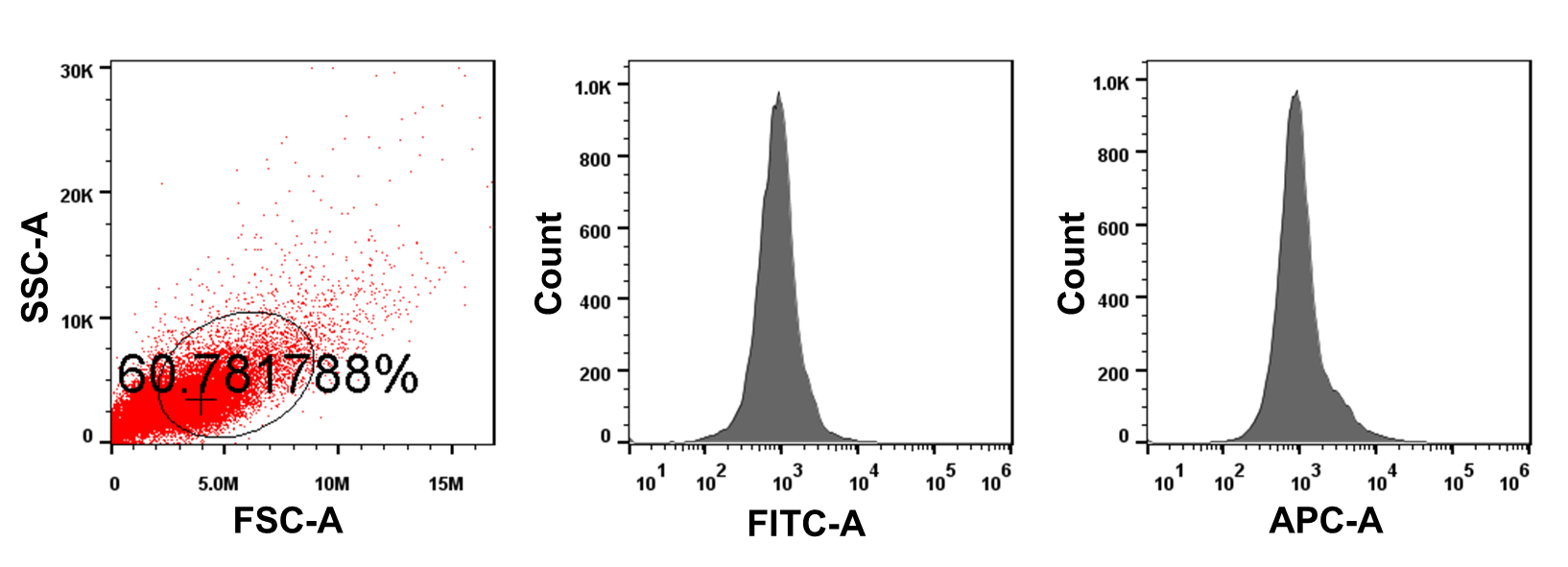
**

## Figure S36. The FCM gating strategy (the control group as a representative example).

**
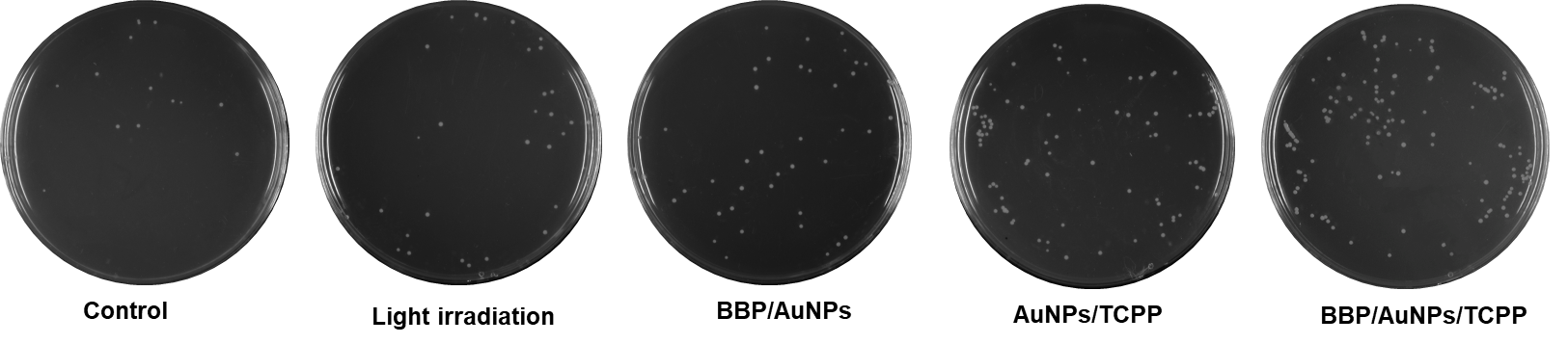
**

## Figure S37. Photographs of colonies within live bacteria within macrophages in different treatment.


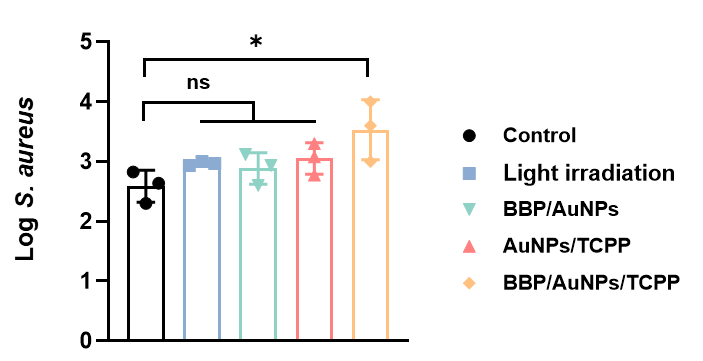


## Figure S38. Quantification of live bacteria within macrophage after different treatments.

## Table S1. Sequences of the peptides screened from Ph.D.-12 peptide library.

| **No.** | **Peptide Sequence** | **Ratio** |
| --- | --- | --- |
| B-1 | V H K H H F G H G P K P | 3/20 |
| B-2 | L P R H H H H Y F T S P | 2/20 |
| B-3 | W S P R H D H H R H S G | 2/20 |
| B-4 | H V R Q H D A L V G Y A | 1/20 |
| B-5 | T L H R G L V L E E M E | 1/20 |
| B-6 | G H K I H V H K H Q M F | 1/20 |
| B-7 | H M R H N H Q Q A H Q A | 1/20 |
| B-8 | W P H H S H R L P S Y N | 1/20 |
| B-9 | W Y R A P H Q P H N H A | 1/20 |
| B-10 | H S W R H H S V S E R W | 1/20 |
| B-11 | Y D I P S S T H C D P P | 1/20 |
| B-12 | H T T K H H H R P M W S | 1/20 |
| B-13 | K P F H T H R H L G V S | 1/20 |
| B-14 | T L H R G L V L E E M E | 1/20 |
| B-15 | F P S H H S H G K Y R H | 1/20 |
| B-16 | H S T H H S Q L H S M R | 1/20 |

## Table S2. The hydrogen bond sites and lengths between PIA and the peptide B-1.

| **No.** | **Binding** | **Distance (A°）** |
| --- | --- | --- |
| 1 | Val-1…THR-187 | 2.4 |
| 2 | Val-1…ASN-186 | 2.8 |
| 3 | Val-1…ASN-186 | 2.9 |
| 4 | LYS-3…ASN-186 | 2.2 |
| 5 | HIS-4…SER-287 | 2.6 |
| 6 | HIS-4…TYR-363 | 3.1 |
| 7 | PHE6…SER-287 | 3.3 |
| 8 | HIS-8…TRP290 | 3.1 |
| 9 | PRO-10…SER324 | 2.8 |
| 10 | PRO-10…SER324 | 2.9 |
| 11 | PRO-10…SER324 | 3.3 |
| 12 | LYS-11…THR-328 | 2.0 |
| 13 | LYS-11…THR-328 | 1.8 |

## Table S3. The sterilization rate after various treatments.

|  | **BBP** | **BBP/AuNPs**  **(PTT)** | **AuNPs/TCPP**  **(PDT)** | **BBP/AuNPs/**  **TCPP** | **BBP/AuNPs+**  **AuNPs/TCPP** | **BBP+ AuNPs+**  **TCPP** |
| --- | --- | --- | --- | --- | --- | --- |
| Sterilization rate (%) | 2.33 | 6.99 | 22.44 | 59.73 | 36.24 | 19.46 |
|  | 6.04 | 9.47 | 35.40 | 57.37 | 39.60 | 21.48 |
|  | -0.36 | 19.46 | 34.19 | 62.71 | 40.94 | 25.50 |

## Table S4. The Gene ontology (GO) enrichment analysis of differentially expressed genes.^[1,2]^

| **Term** | **Ontology** | **Signi symbol** | **Core function** |
| --- | --- | --- | --- |
| Large ribosomal subunit | Cellular component | rplK, rplA, rplM, rplQ, rplO, rpmD, rplR, rplF, rplE, rplX, rplN, rpmC, rplP, rplV, rplB, rplW, rplD | The structural integrity of ribosomes; translational function |
| Cytosolic large ribosomal subunit | Cellular component | rplK, rplA, rplM, rplQ, rplO, rpmD, rplR, rplF, rplE, rplX, rplN, rpmC, rplP, rplV, rplB, rplW, rplD | The structural integrity of ribosomes; translational function |
| Ribosome | Cellular component | rpsF, rplK, rplA, rpsL, rpsG, rpsU, rplM, rplQ, rpsK, rpsM, rplO, rpmD, rpsE, rplR, rplF, rpsH, rplE, rplX, rplN, rpsQ, rpmC, rplP, rpsC, rplV, rpsS, rplB, rplW, rplD, rpsJ | Core components of protein synthesis machines |
| Cytosolic ribosome | Cellular component | rpsF, rplK, rplA, rpsG, rplM, rplQ, rpsK, rplO, rpmD, rpsE, rplR, rplF, rpsH, rplE, rplX, rplN, rpsQ, rpmC, rplP, rpsC, rplV, rpsS, rplB, rplW, rplD | Core components of protein synthesis machines |
| mRNA binding | Molecular function | rpsG, pnp, acnA, rplM, rpsK | Post-transcriptional regulation of mRNA stability, degradation, and translation efficiency |
| Fibronectin binding | Molecular function | eno, clfA, fnbB, fnbA | Bacterial adhesion |
| ATP-dependent activity | Molecular function | gyrB, dnaB, ftsH, secA, addB, ABQ280_RS05380, ftsA, priA, parE, ABQ280_RS07635, ABQ280_RS10485, kdpA, atpE, secA2 | DNA repair; secretory system functions, |
| Structural constituent of ribosome | Molecular function | rpsF, rplK, rpsL, rpsG, rplM, rplQ, rpsK, rpsM, rplO, rpsE, rplF, rpsH, rplE, rplN, rpsQ, rplP, rpsC, rplV, rpsS, rplB, rplW, rplD, rpsJ | Core components of protein synthesis machines |
| rRNA processing | Biological process | rnmV, rsmA, ABQ280_RS02555, rlmB, rplA, ABQ280_RS05090, rsmB, rlmN, yhaM, rpsK, rplC, rsmG | Ribosome assembly |
| Carbohydrate phosphorylation | Biological process | pfkB, ABQ280_RS07580, pfkA, lacC | Sugar phosphorylation activates energy metabolism |
| Ribosome biogenesis | Biological process | rnmV, rsmA, ABQ280_RS02555, rlmB, rplK, rplA, rpsG, ABQ280_RS05090, rsmB, rlmN, rsfS, yqeH, yhaM, rpsK, rpsM, rplE, rpsS, rplW, rplC, rsmG | Ribosome assembly |
| Purine ribonucleotide biosynthetic process | Biological process | guaB, guaA, ABQ280_RS02315, ABQ280_RS03840, tpiA, gpmI, eno, purQ, purL, coaE, pfkA, ABQ280_RS10620, atpE | DNA/RNA synthesis, bacterial division |
| Translation | Biological process | metG, cysS, rplK, rplA, rpsL, rpsG, pheS, pheT, rsfS, alaS, ABQ280_RS08120, leuS, rplM, rpsK, rpsM, rpsE, rplF, rplE, rplX, rpsQ, rpsC, rplB, rplW, rplD, rpsJ | The protein synthesis machinery |
| ATP metabolic process | Biological process | ABQ280_RS03840, tpiA, gpmI, eno, qoxC, qoxB, pfkA, ABQ280_RS10620, atpE | Energy metabolism |

## Table S5. The Kyoto Encyclopedia of Genes and Genomes (KEGG) functional enrichment analysis of differentially expressed genes.^[3-5]^

| **Metabolic pathway** | **Signi symbol** | **Core function** |
| --- | --- | --- |
| Lipoic acid metabolism | ABQ280_RS05135, ABQ280_RS05130, pdhA, lpdA, ABQ280_RS06885, sucB, lipA, lpdA, gcvPB, gcvPA, ABQ280_RS07425, ABQ280_RS07420, gcvT, ABQ280_RS01480 | Energy production |
| Mismatch repair | ABQ280_RS05995, ssb, ligA, ABQ280_RS08325, pcrA, ABQ280_RS09555, ABQ280_RS05380, xseA, dnaX, ABQ280_RS07450, ssb | DNA damage |
| Fatty acid biosynthesis | accC, fabF, fabD, fabG, ABQ280_RS04540, accD, ABQ280_RS07860, ABQ280_RS07865 | Membrane/wall synthesis |
| Ribosome | rplB, rpsE, rpsU, rplD, rpsG, rpsL, rplV, rplP, rpsC, rpsS, rplC, rplK, rplN, rplE, rpsJ, rpsK, rplR, rpsM, rplX, rpmD, rpmC, rpsF, ABQ280_RS11315, rplO, rpsH, rplW, rplA, rplF, rpsQ, ABQ280_RS02625, rpmG, rplM, rpmJ, rplQ | Protein synthesis, translation regulation |
| Starch and sucrose metabolism | treP, ABQ280_RS10260, ABQ280_RS10265, ABQ280_RS01140, ABQ280_RS07580 | Biofilm matrix integrity |
| Quorum sensing | secA, secY, secA2, ribD, ftsY, ABQ280_RS10510, ABQ280_RS04755, sspA, agrB, ABQ280_RS04565, ABQ280_RS04570, secE, ABQ280_RS04575, lacD, ABQ280_RS06660, splA, ABQ280_RS00820, secY2, agrD | Virulence factor synthesis |
| Purine metabolism | guaB, ABQ280_RS11275, deoB, guaA, ABQ280_RS08005, ABQ280_RS02315, deoD, ureC, purH, purF, purM, purN, purD, ABQ280_RS11585, ABQ280_RS11580, arcC, purC, purK, xpt, guaC | DNA damage |
| Citrate cycle (TCA cycle) | ABQ280_RS05135, ABQ280_RS05130, pdhA, lpdA, sdhA, ABQ280_RS06120, ABQ280_RS06885, sucB, lpdA, sucC, acnA | Energy production |

References

1. D. N. Wilson, “Ribosome-targeting antibiotics and mechanisms of bacterial resistance,” Nature Reviews Microbiology **12** (2014): 35-48. <https://doi.org/10.1038/nrmicro3155>
2. T. Foster, J. Geoghegan, V. Ganesh, and M. Höök, “Adhesion, invasion and evasion: the many functions of the surface proteins of Staphylococcus aureus,” Nature Reviews Microbiology **12** (2014): 49. https://doi.org/10.1038/nrmicro3161
3. J. E. Cronan, “Assembly of Lipoic Acid on Its Cognate Enzymes: an Extraordinary and Essential Biosynthetic Pathway,” Microbiology and Molecular Biology Reviews **80** (2016): 429. <https://doi.org/10.1128/mmbr.00073-15>
4. H. C. Flemming, E. D. van Hullebusch, T. R. Neu, Per H. Nielsen, T. Seviour, P. Stoodley, J. Wingender, and S. Wuertz, “The biofilm matrix: multitasking in a shared space,” Nature Reviews Microbiology **21** (2023): 70. https://doi.org/10.1038/s41579-022-00791-0
5. E. Wall, N. Majdalani, and S. Gottesman, “The Complex Rcs Regulatory Cascade,” Annual Review of Microbiology **72** (2018): 111. https://doi.org/10.1146/annurev-micro-090817-062640
