# Supplementary material for: Biofilm‐Binding Phages Enhance Biofilm Eradication by Synergistic Photothermal and Photodynamic Therapy
Source: Adv Sci (Weinh). 2026 Jul 8:e23904. Online ahead of print. doi: 10.1002/advs.202523904 (PMC13345313; doi:10.1002/advs.202523904)

Figure 4F

S. aureus:

Control

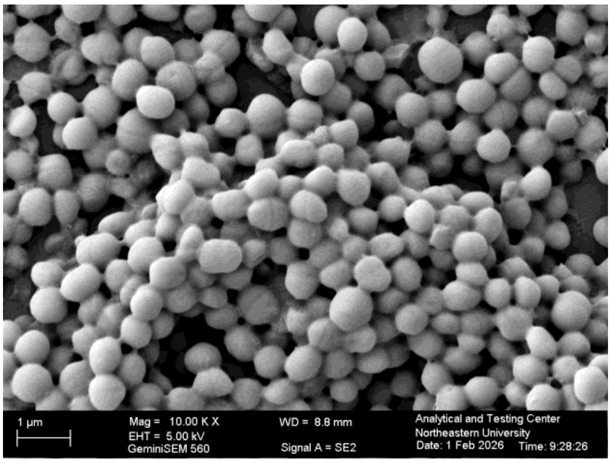

Light irradiation

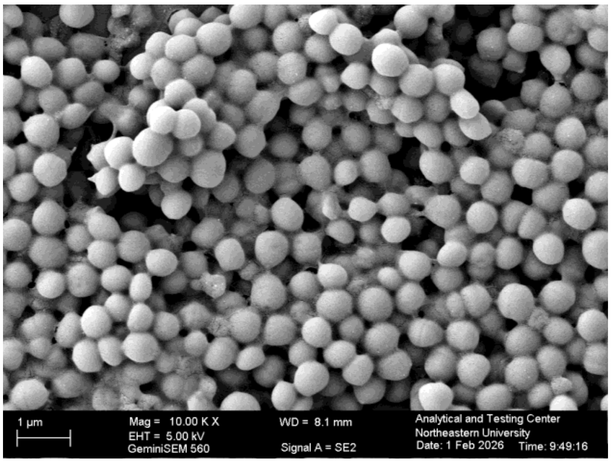

WT-M13/AuNPs

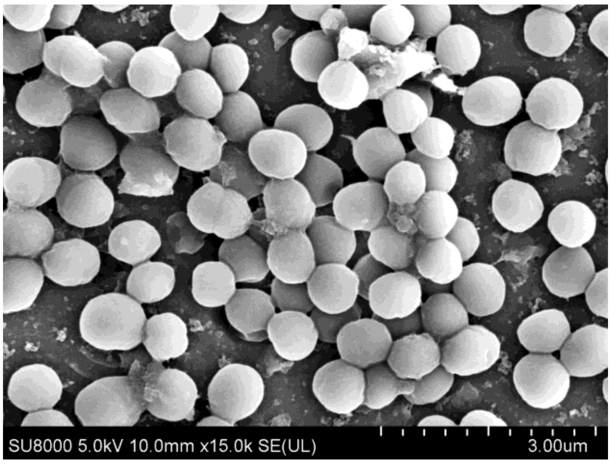

BBP/AuNPs

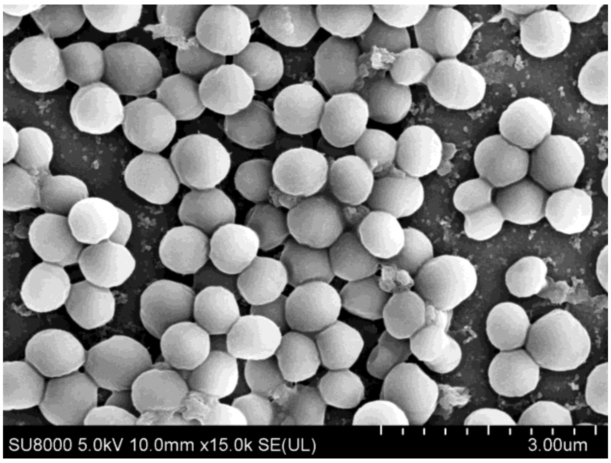

AuNPs/TCPP

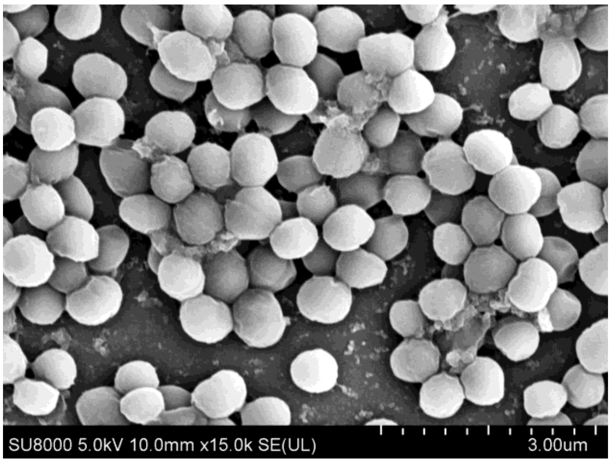

WT-M13/AuNPs/TCPP

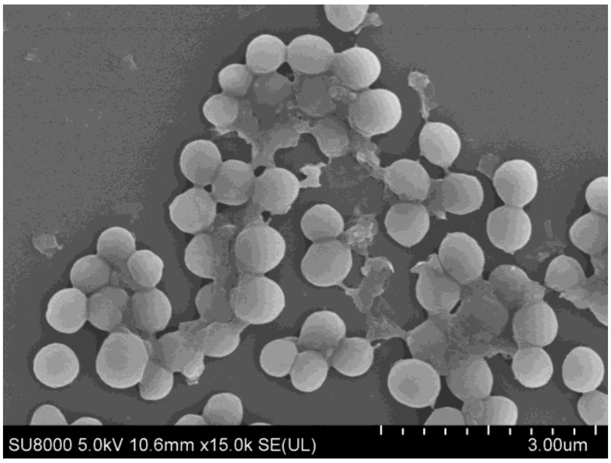

BBP/AuNPs/TCPP

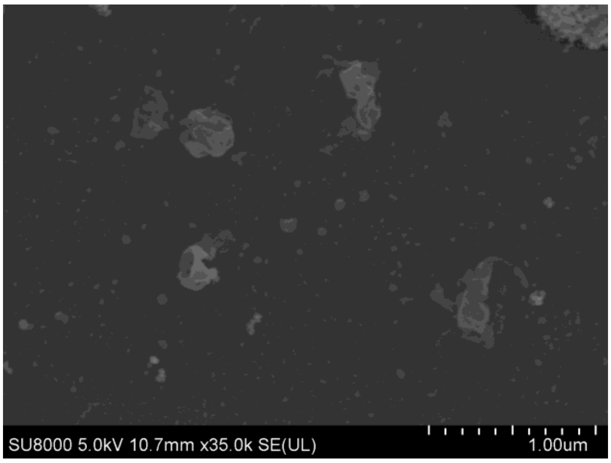

MRSA:

Control

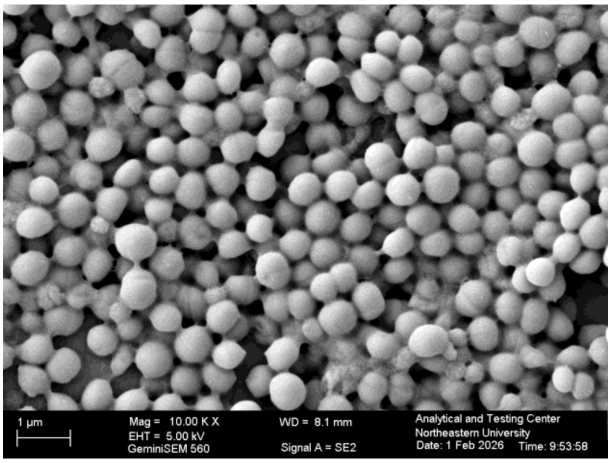

Light irradiation

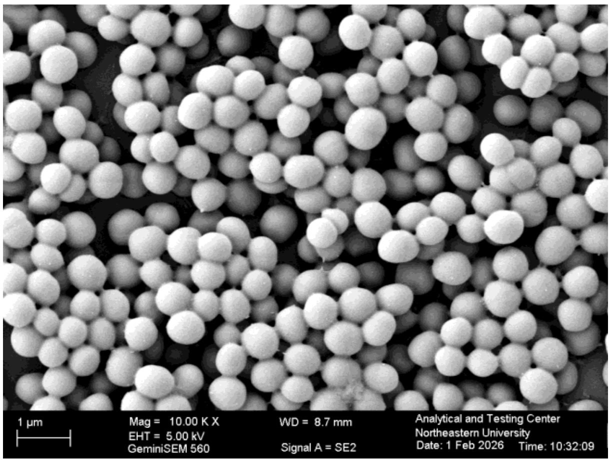

WT-M13/AuNPs

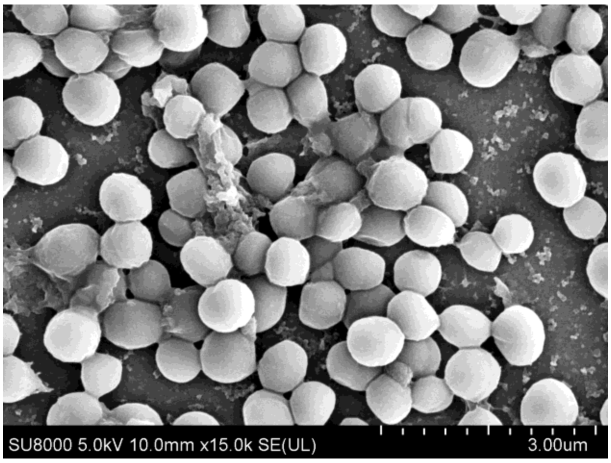

BBP/AuNPs

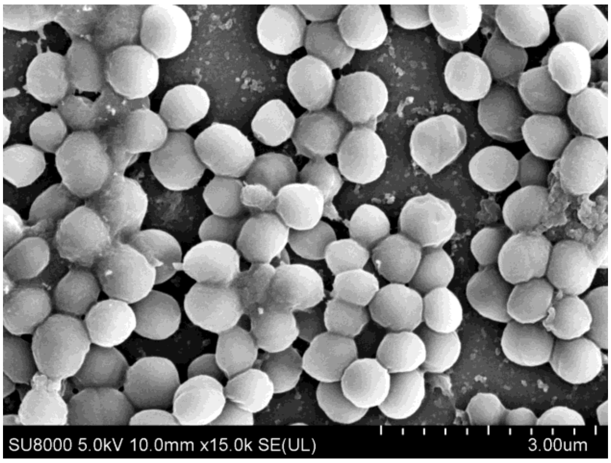

AuNPs/TCPP

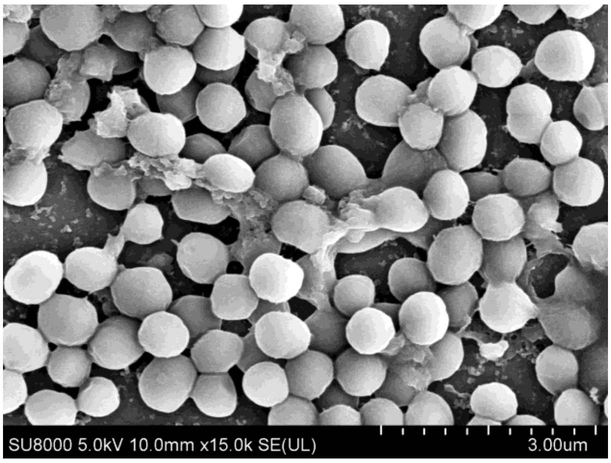

WT-M13/AuNPs/TCPP

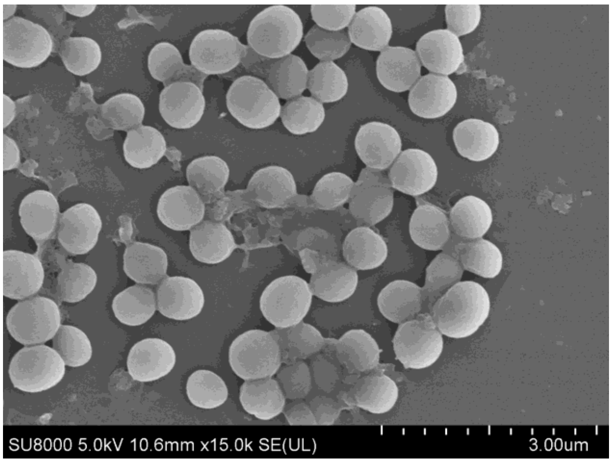

BBP/AuNPs/TCPP

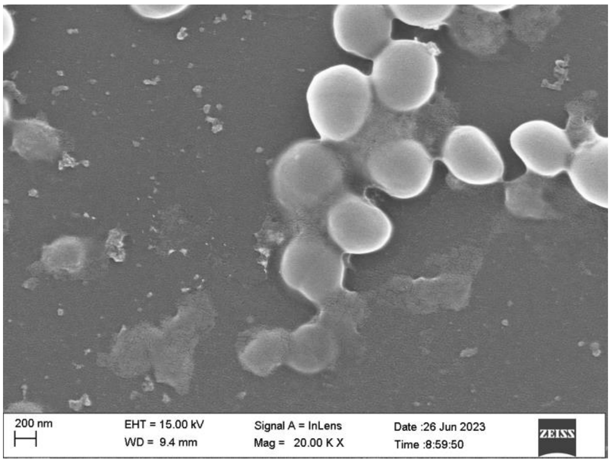

Figure S28

Control

Heart

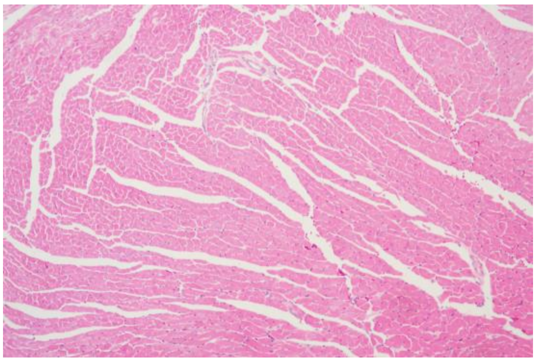

Liver

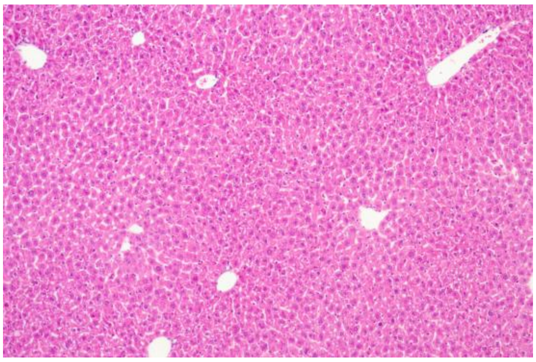

Spleen

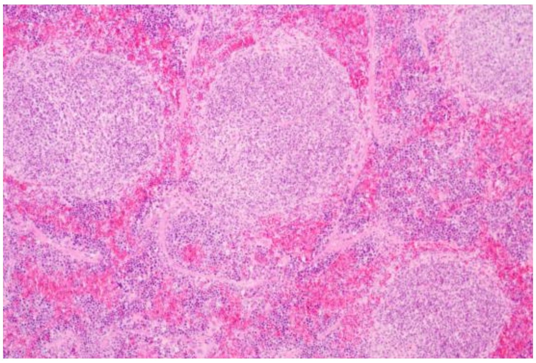

Lung

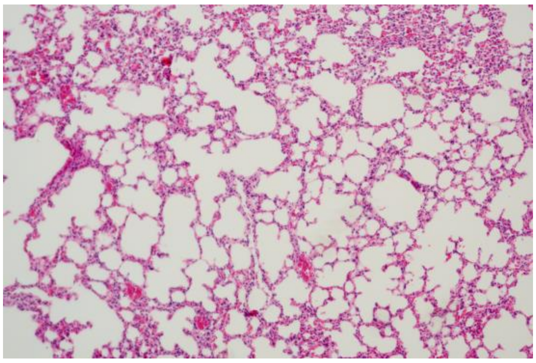

Kidney

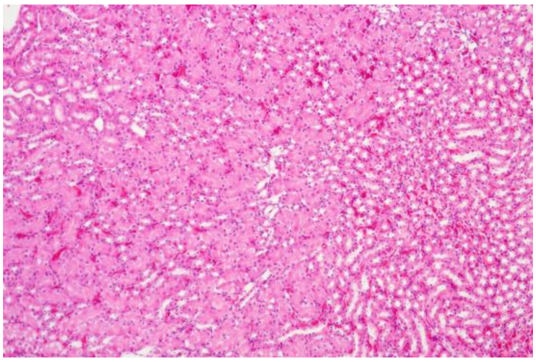

Light irradiation

Heart

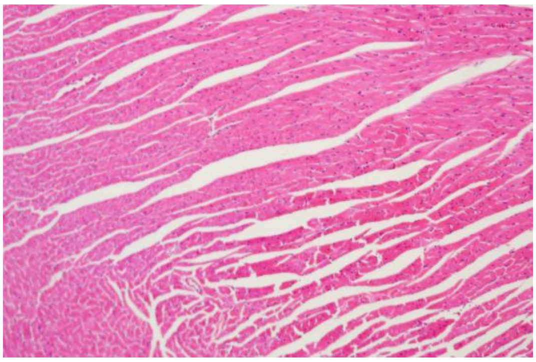

Liver

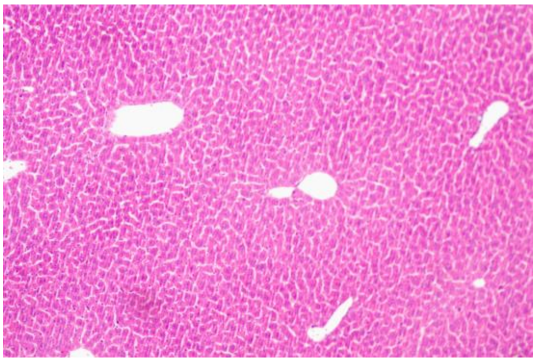

Spleen

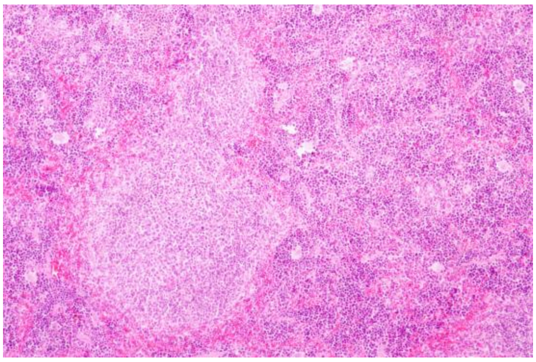

Lung

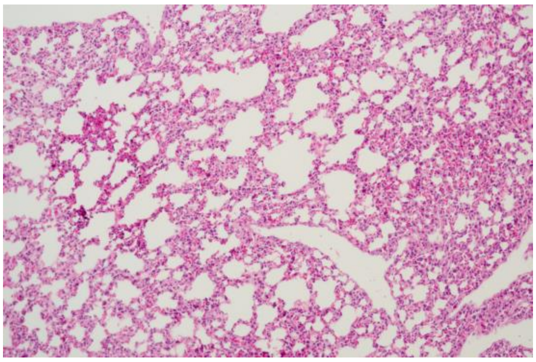

Kidney

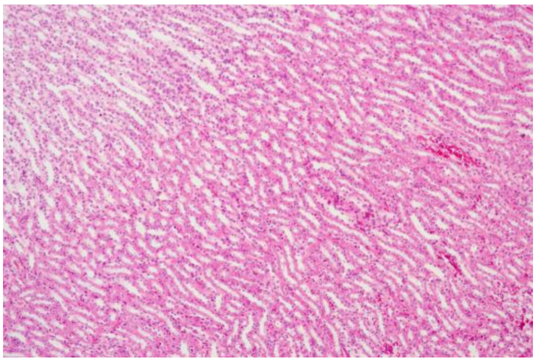

WT-M13/AuNPs

Heart

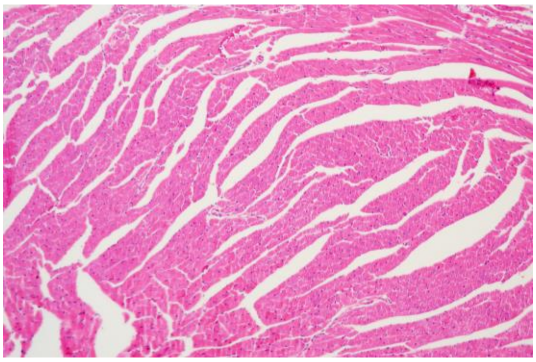

Liver

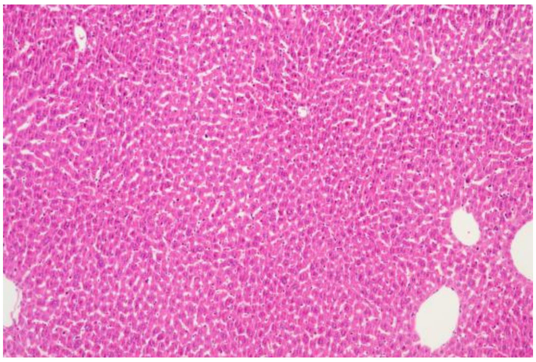

Spleen

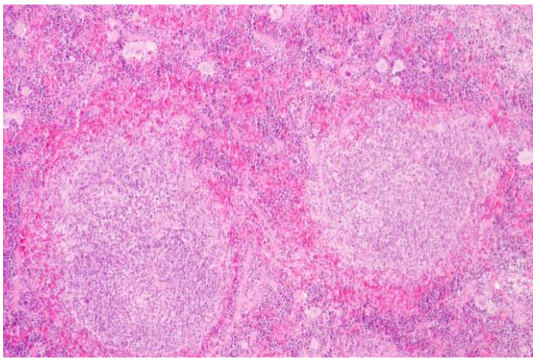

Lung

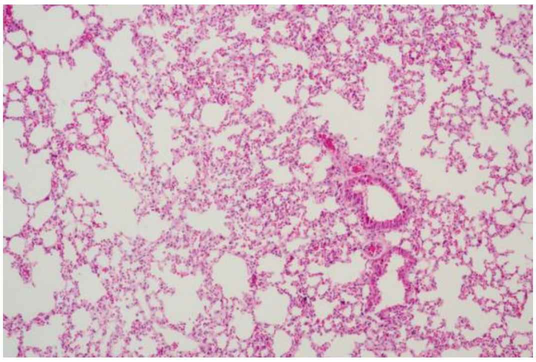

Kidney

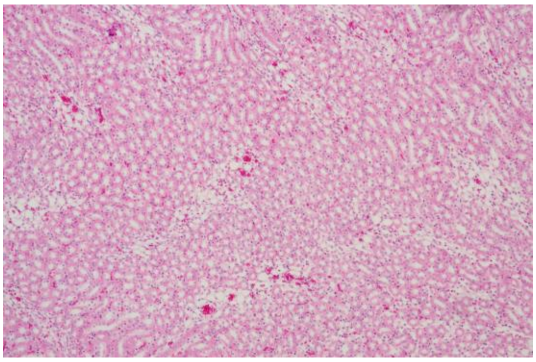

BBP/AuNPs

Heart

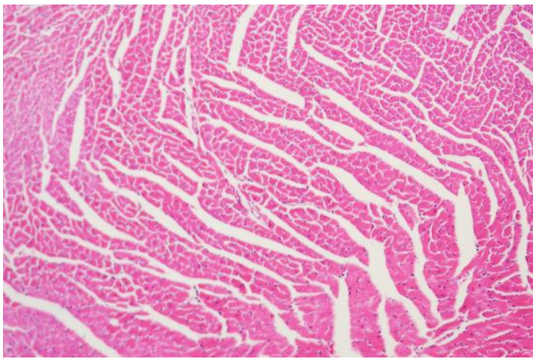

Liver

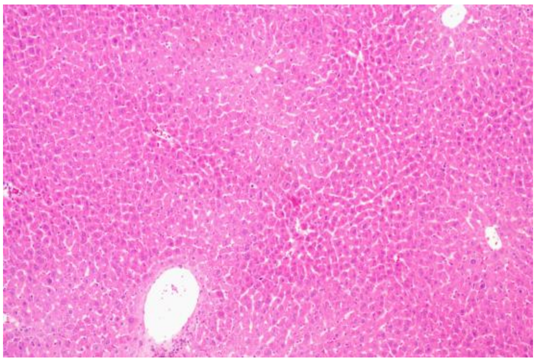

Spleen

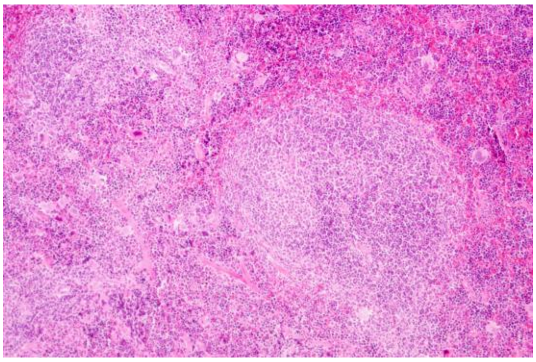

Lung

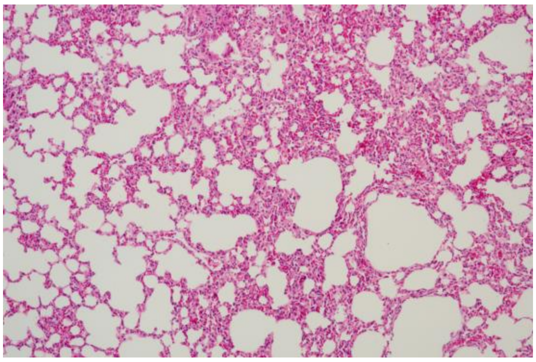

Kidney

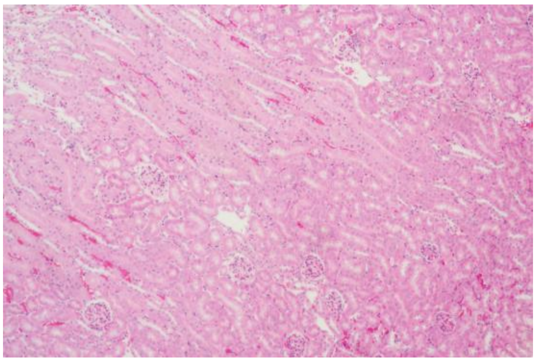

AuNPs/TCPP

Heart

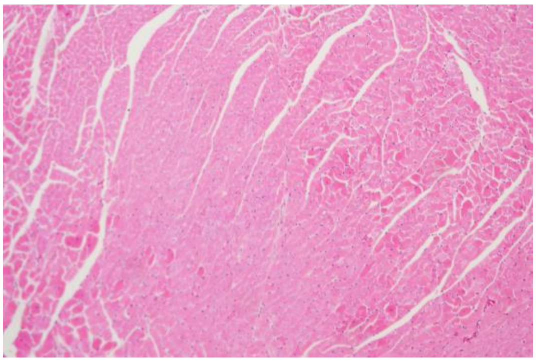

Liver

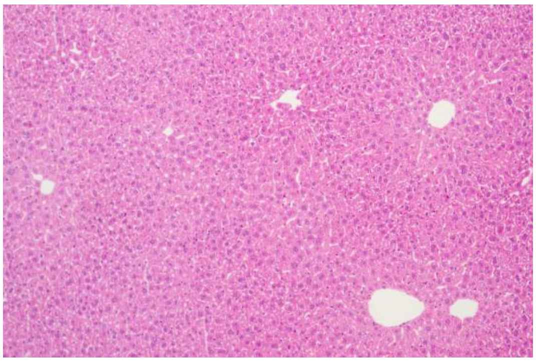

Spleen

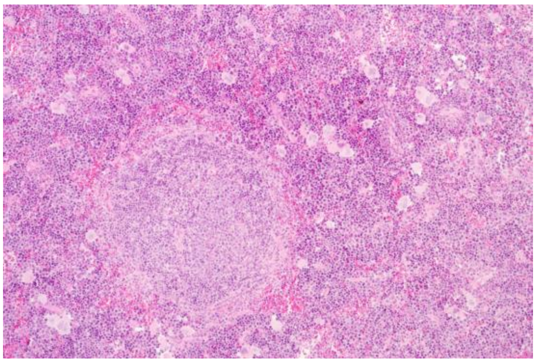

Lung

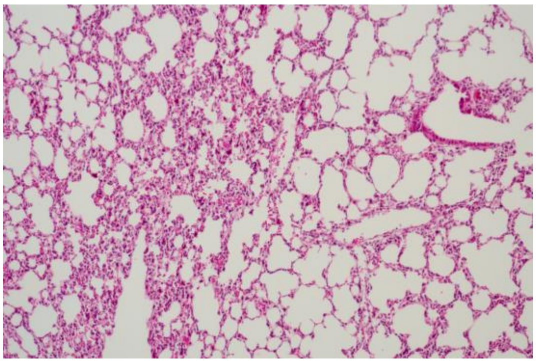

Kidney

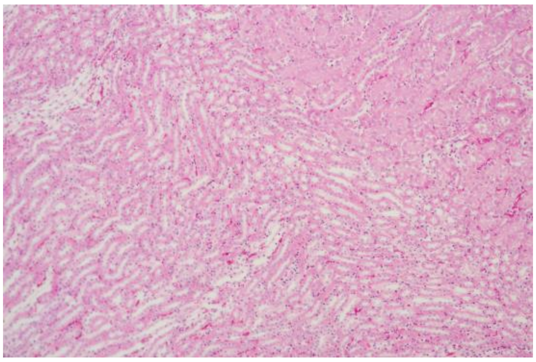

WT-M13/AuNPs/TCPP

Heart

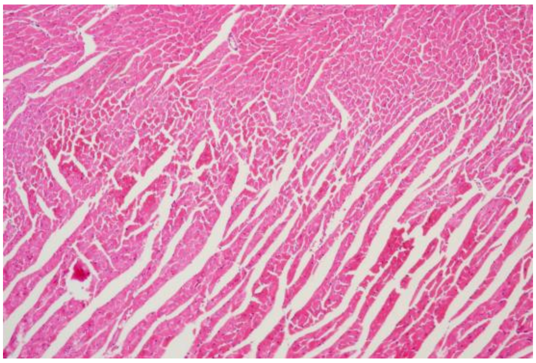

Liver

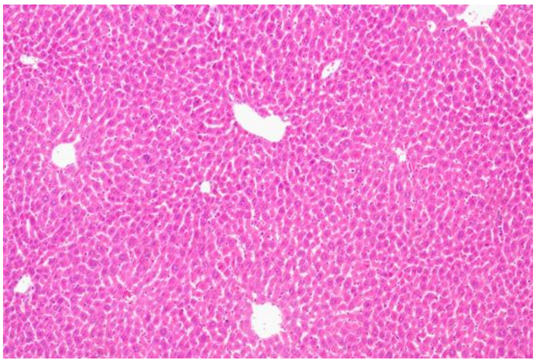

Spleen

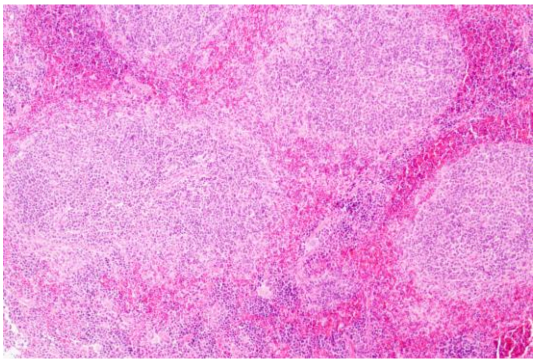

Lung

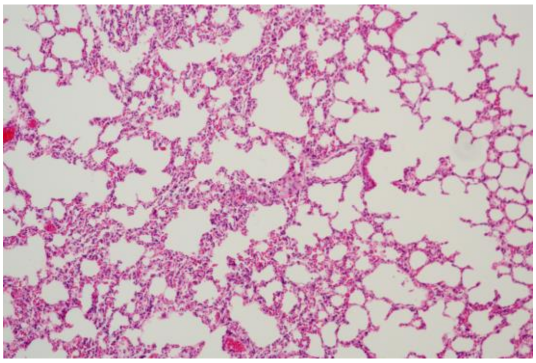

Kidney

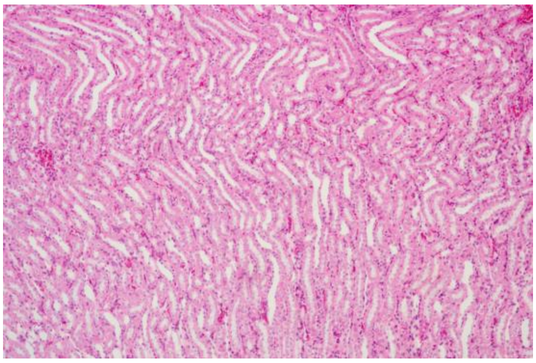

BBP/AuNPs/TCPP

Heart

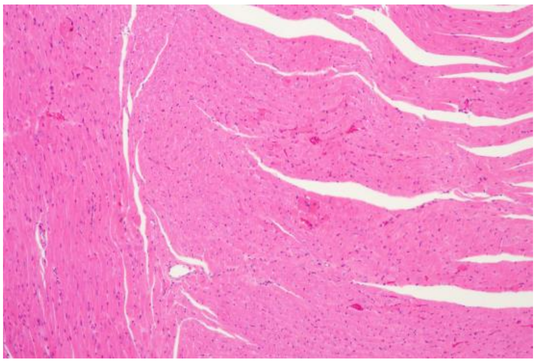

Liver

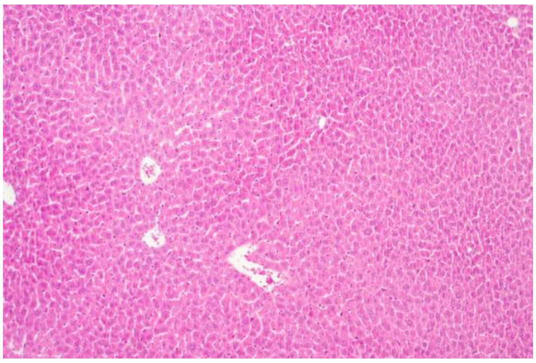

Spleen

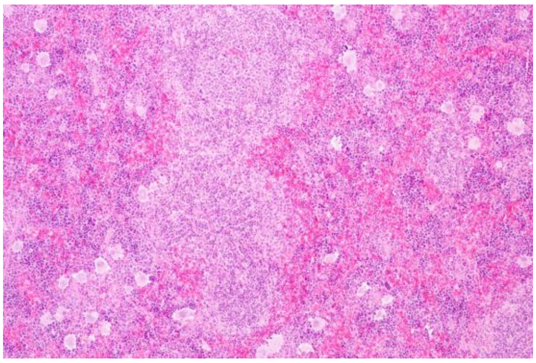

Lung

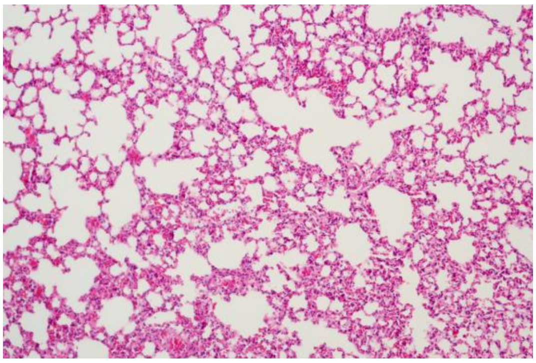

Kidney

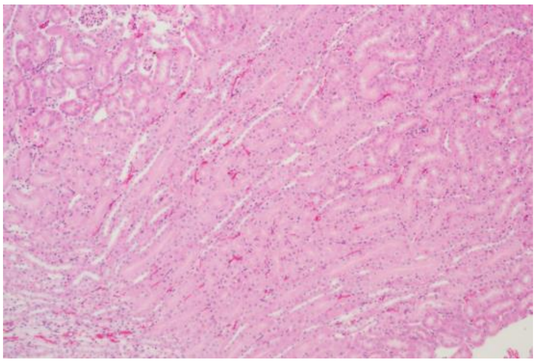

Figure S33

Control

Heart

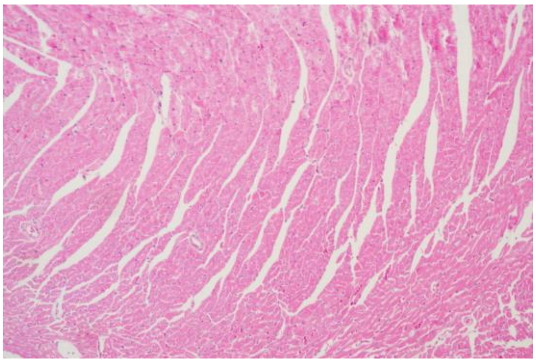

Liver

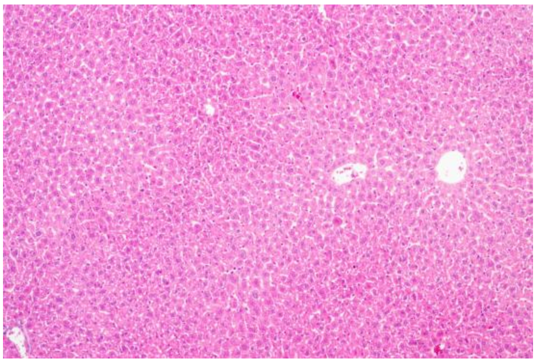

Spleen

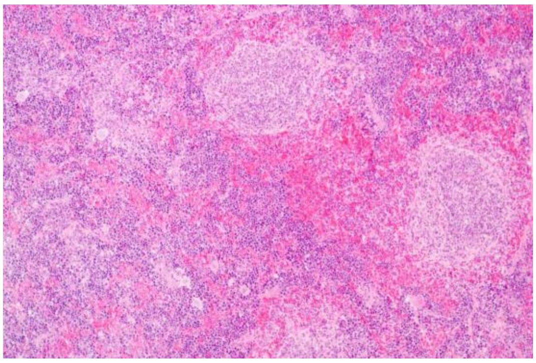

Lung

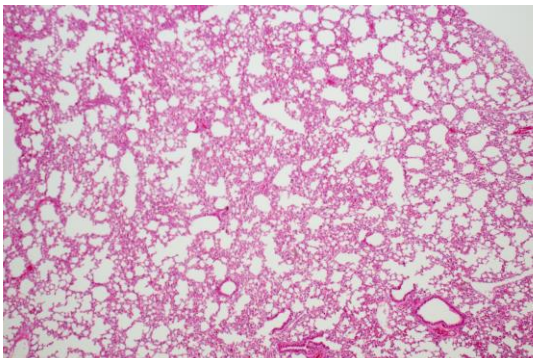

Kidney

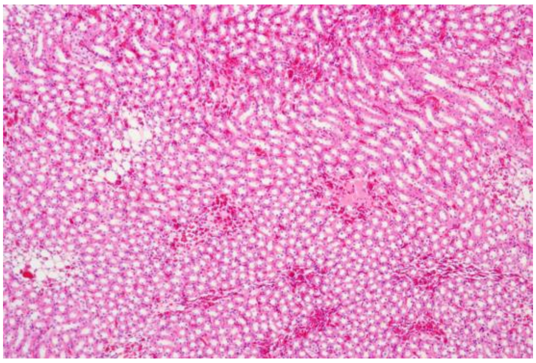

Light irradiation

Heart

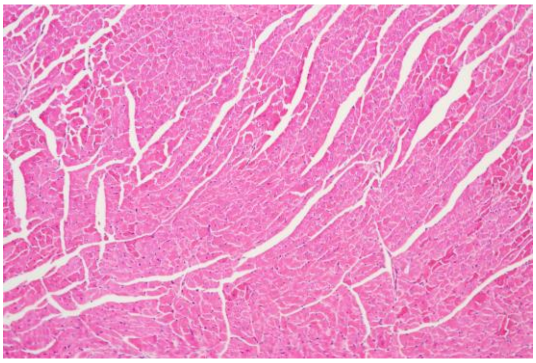

Liver

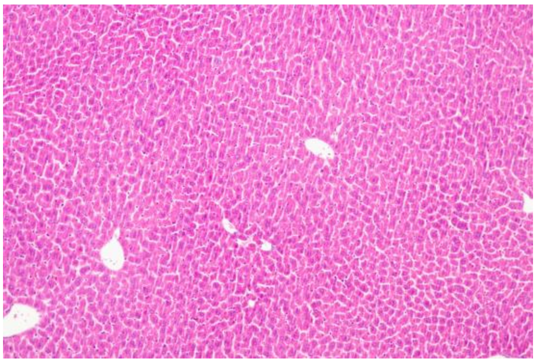

Spleen

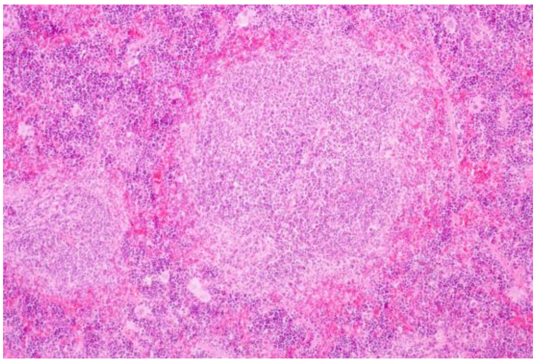

Lung

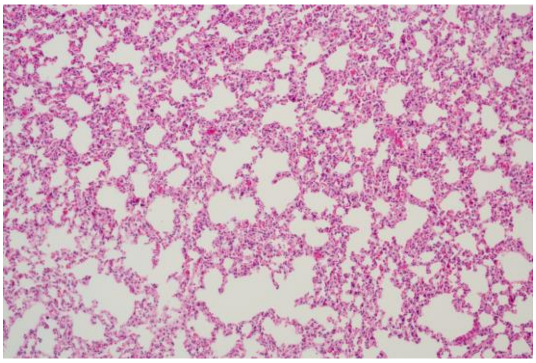

Kidney

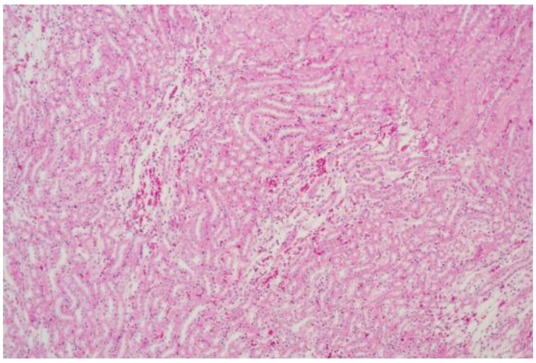

WT-M13/AuNPs

Heart

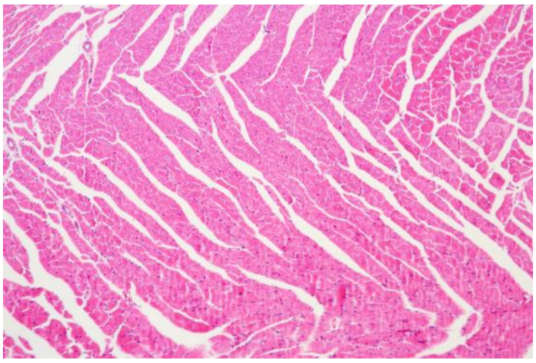

Liver

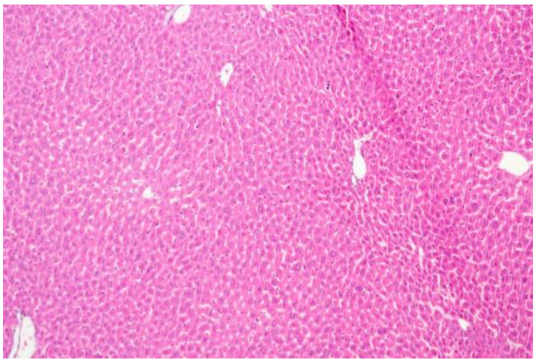

Spleen

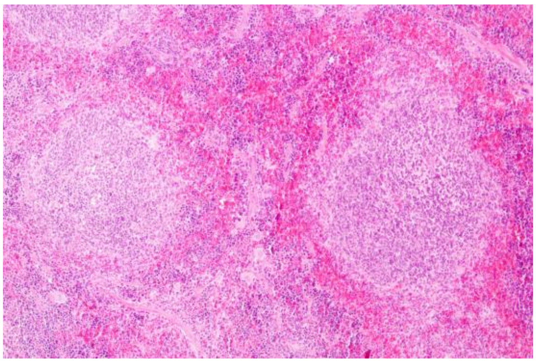

Lung

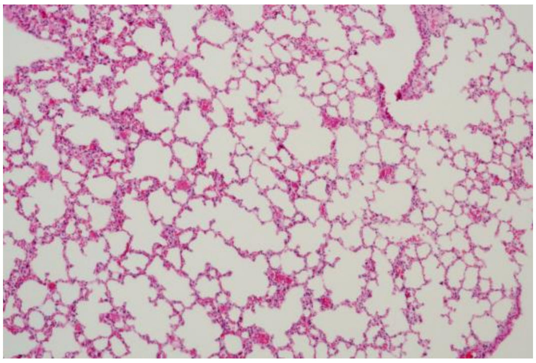

Kidney

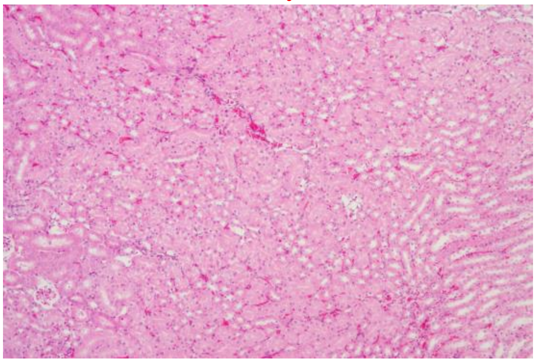

BBP/AuNPs

Heart

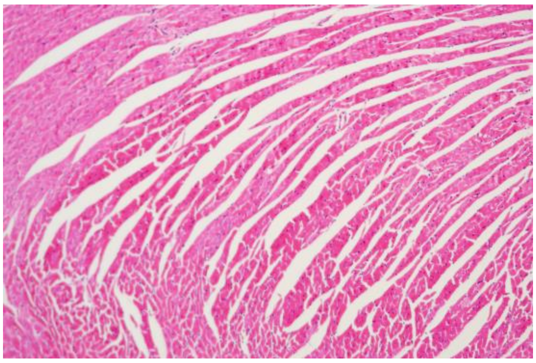

Liver

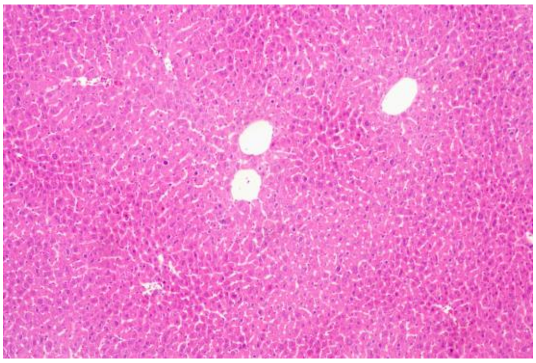

Spleen

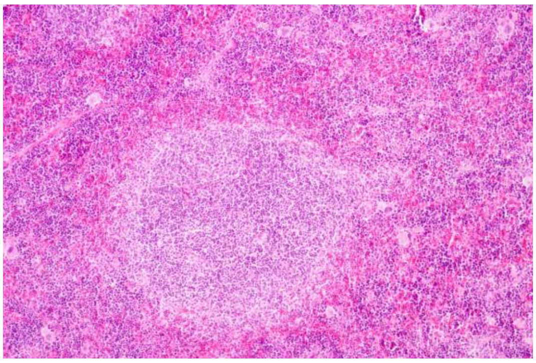

Lung

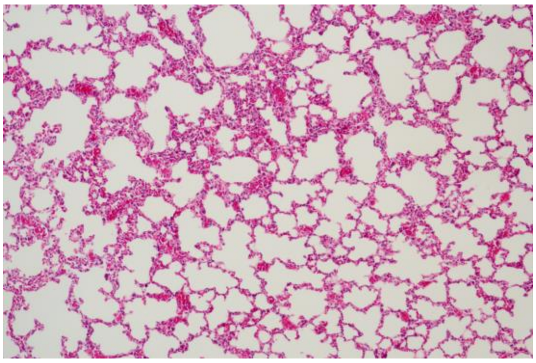

Kidney

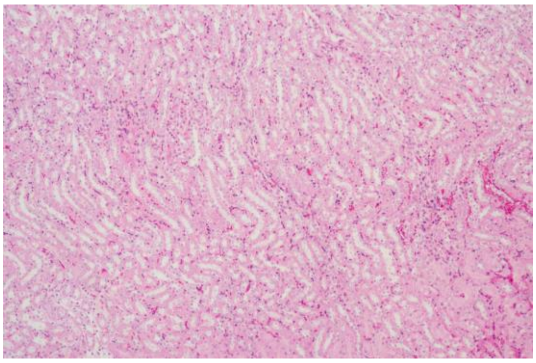

AuNPs/TCPP

Heart

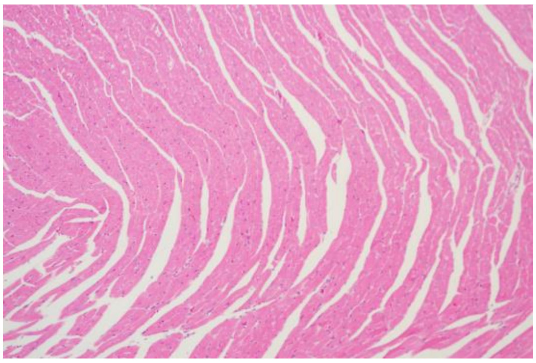

Liver

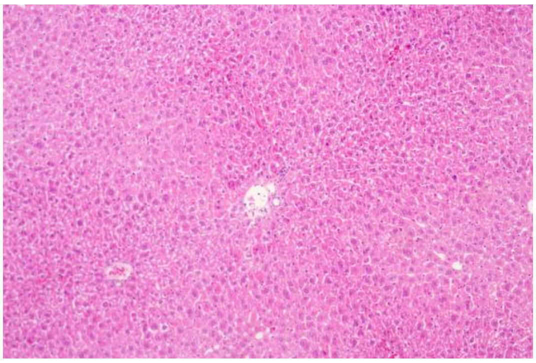

Spleen

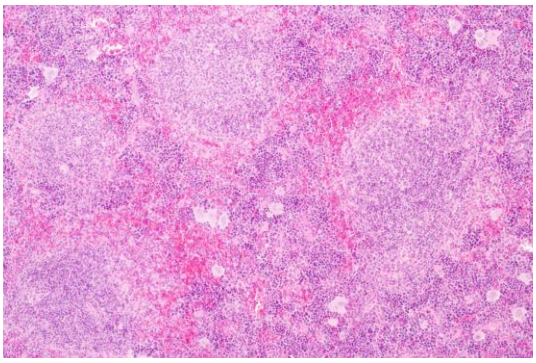

Lung

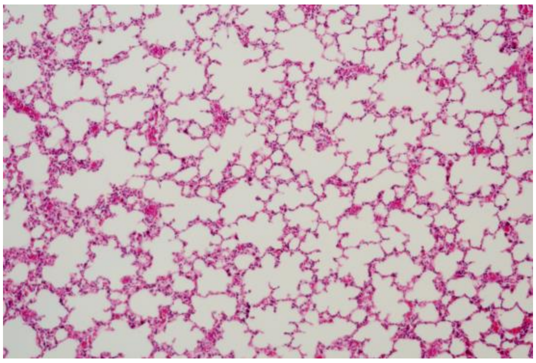

Kidney

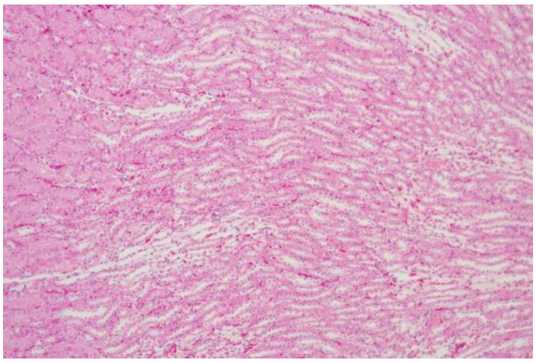

WT-M13/AuNPs/TCPP

Heart

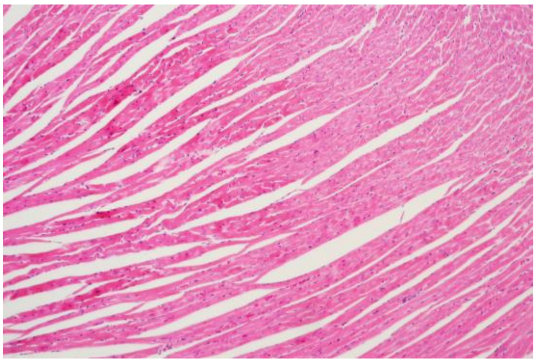

Liver

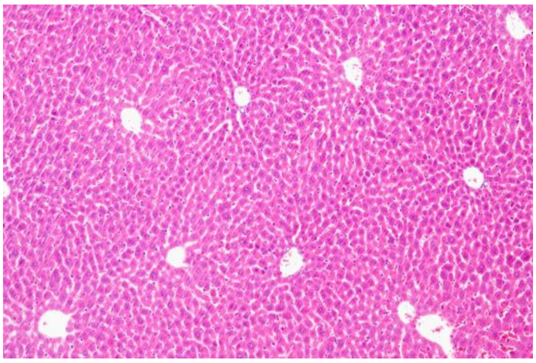

Spleen

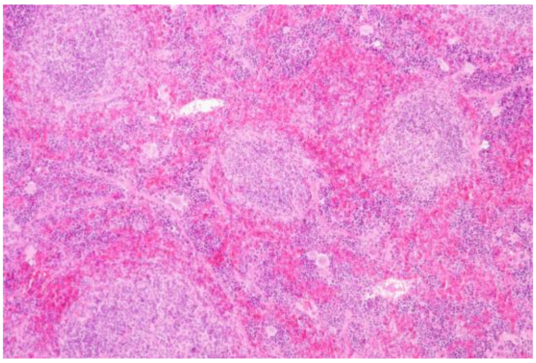

Lung

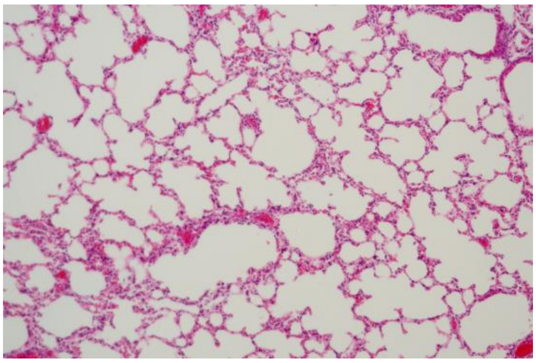

Kidney

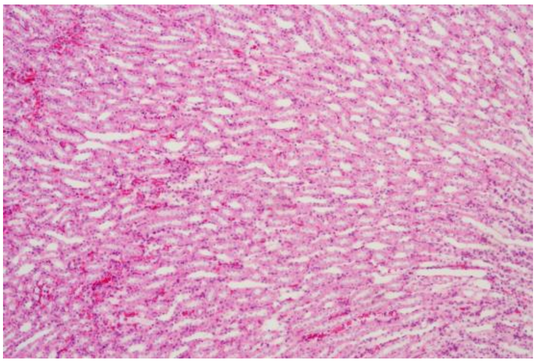

BBP/AuNPs/TCPP

Heart

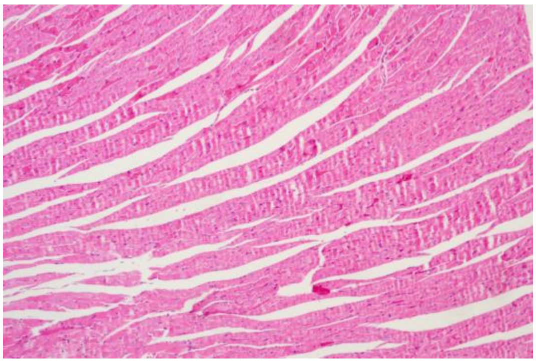

Liver

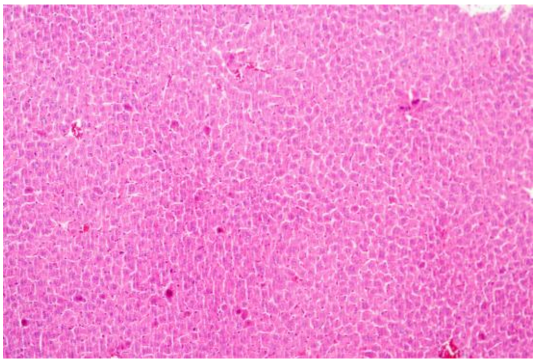

Spleen

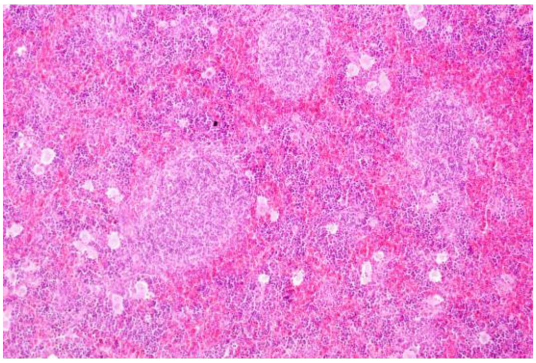

Lung

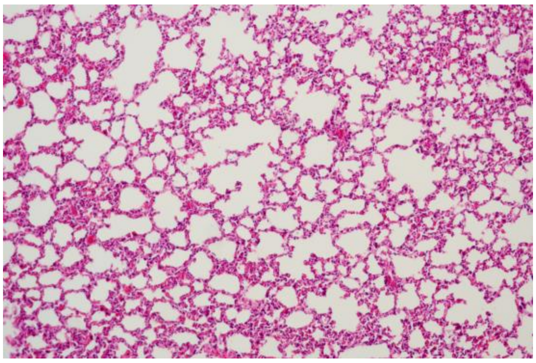

Kidney

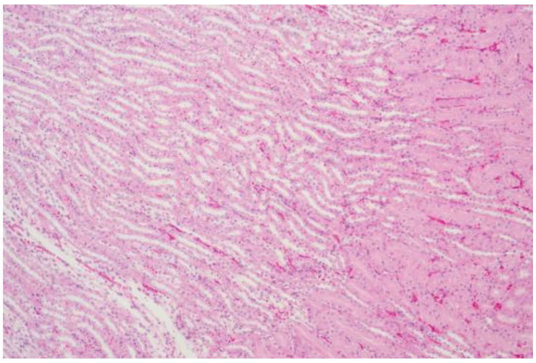

Supplement: Supplementary file 2 — Supporting File 2: advs76405‐sup‐0002‐DataFile.pdf. [file ADVS-9999-e23904-s002.pdf]
